# Therapeutic potential of targeting S100A11 in malignant pleural mesothelioma

**DOI:** 10.1038/s41389-017-0017-3

**Published:** 2018-01-24

**Authors:** Hiroki Sato, Masakiyo Sakaguchi, Hiromasa Yamamoto, Shuta Tomida, Keisuke Aoe, Kazuhiko Shien, Takahiro Yoshioka, Kei Namba, Hidejiro Torigoe, Junichi Soh, Kazunori Tsukuda, Hiroyuki Tao, Kazunori Okabe, Shinichiro Miyoshi, Harvey I. Pass, Shinichi Toyooka

**Affiliations:** 1 Department of General Thoracic Surgery and Breast and Endocrinological Surgery, Okayama University Graduate School of Medicine, Dentistry and Pharmaceutical Sciences, Okayama, Japan; 2 Department of Cell Biology, Okayama University Graduate School of Medicine, Dentistry and Pharmaceutical Sciences, Okayama, Japan; 3 Department of Bioinformatics, Okayama University Graduate School of Medicine, Dentistry and Pharmaceutical Sciences, Okayama, Japan; 4 Department of Medical Oncology, National Hospital Organization Yamaguchi-Ube Medical Center, Ube, Yamaguchi Japan; 5 Department of Clinical Research, National Hospital Organization Yamaguchi-Ube Medical Center, Ube, Yamaguchi Japan; 6 Department of Clinical Genomic Medicine, Okayama University Graduate School of Medicine, Dentistry and Pharmaceutical Sciences, Okayama, Japan; 7 Department of Thoracic Surgery, National Hospital Organization Yamaguchi-Ube Medical Center, Ube, Yamaguchi Japan; 8 Department of Cardiothoracic Surgery, New York University Langone Medical Center, New York, NY USA

## Abstract

Malignant pleural mesothelioma (MPM) is an aggressive tumor with an unfavorable prognosis. The standard therapeutic approaches are limited to surgery, chemotherapy, and radiotherapy. Because the consequent clinical outcome is often unsatisfactory, a different approach in MPM treatment is required. S100A11, a Ca^2+^-binding small protein with two EF-hands, is frequently upregulated in various human cancers. Interestingly, it has been found that intracellular and extracellular S100A11 have different functions in cell viability. In this study, we focused on the impact of extracellular S100A11 in MPM and explored the therapeutic potential of an S100A11-targeting strategy. We examined the secretion level of S100A11 in various kinds of cell lines by enzyme-linked immunosorbent assay. Among them, six out of seven MPM cell lines actively secreted S100A11, whereas normal mesothelial cell lines did not secrete it. To investigate the role of secreted S100A11 in MPM, we inhibited its function by neutralizing S100A11 with an anti-S100A11 antibody. Interestingly, the antibody significantly inhibited the proliferation of S100A11-secreting MPM cells in vitro and in vivo. Microarray analysis revealed that several pathways including genes involved in cell proliferation were negatively enriched in the antibody-treated cell lines. In addition, we examined the secretion level of S100A11 in various types of pleural effusions. We found that the secretion of S100A11 was significantly higher in MPM pleural effusions, compared to others, suggesting the possibility for the use of S100A11 as a biomarker. In conclusion, our results indicate that extracellular S100A11 plays important roles in MPM and may be a therapeutic target in S100A11-secreting MPM.

## Introduction

Malignant pleural mesothelioma (MPM) is a highly invasive and aggressive tumor that develops in the mesothelial lining of the pleura. The median survival of patients with MPM from the time of diagnosis is usually less than 1 year^1,2^. While surgical resection is the treatment of first choice for early-stage disease, recurrence of the disease often makes the prognosis poorer. In addition, most MPM cases are of advanced-stage disease, for which the benefits of a standard chemotherapeutic regimen with cisplatin and pemetrexed are very limited. These considerations demand the development of novel therapeutic strategies for MPM.

Proteins of the S100 family are small molecules (ranging from 9 to 14 kDa) with two EF-hands and in humans, the family is composed of 20 different members (S100A1–S100A16, S100β, S100G, S100P, and S100Z). This group of proteins modulates a variety of cellular processes, including cell proliferation, differentiation, and intracellular signaling by functioning both as intracellular Ca^2+^ sensors and as extracellular factors^3–5^.

S100A11, also called S100C or calgizzarin, was cloned from chicken gizzard in 1991^6^. We previously reported that S100A11 has two ambivalent functions in the cells. Namely, in the cytoplasmic compartment, S100A11 inhibits the growth of normal human keratinocytes in response to high Ca^2+^ or transforming growth factor β^7,8^. Contrarily, the binding of extracellular S100A11 to the receptor for advanced glycation end products (RAGE) enhances the production of epidermal growth factor family proteins, resulting in growth stimulation^5,9^. Based on these findings, we have studied the biological activity of S100A11 by focusing both on intracellular and extracellular S100A11. As for the function of intracellular S100A11, we have shown that the intracellular S100A11–ANXA2 complex helps plasma membrane repair, which was critical for survival and metastasis, in metastatic breast cancer cell line^10^. Additionally, it is reported that intracellular S100A11 promotes pseudopodial actin dynamics, which plays a critical role in tumor metastasis and the suppression of S100A11 results in inhibition of cell migration and invasion, and the reversion of Epithelial to mesenchymal transition (EMT) in various metastatic cell lines^11^. Regarding extracellular S100A11, we have recently reported that, in mesothelioma cells, S100A11 dimerizes in the peroxisome after transportation of monomeric S100A11 through the interaction with PEX14, an essential component of peroxisomal import machinery, and actively secreted^12^. However, despite advances in the understanding of the biological activity and mechanisms of this protein, little is known about its therapeutic or diagnostic potential. In this study, we investigated the relationship between extracellular S100A11 and MPM, and explored the possibility of an intervention in S100A11 function for MPM treatment and diagnosis.

## Results

### Secretion levels of S100A11 in malignant cell lines and overexpression of S100A11 in MPM

**Fig. 1:**
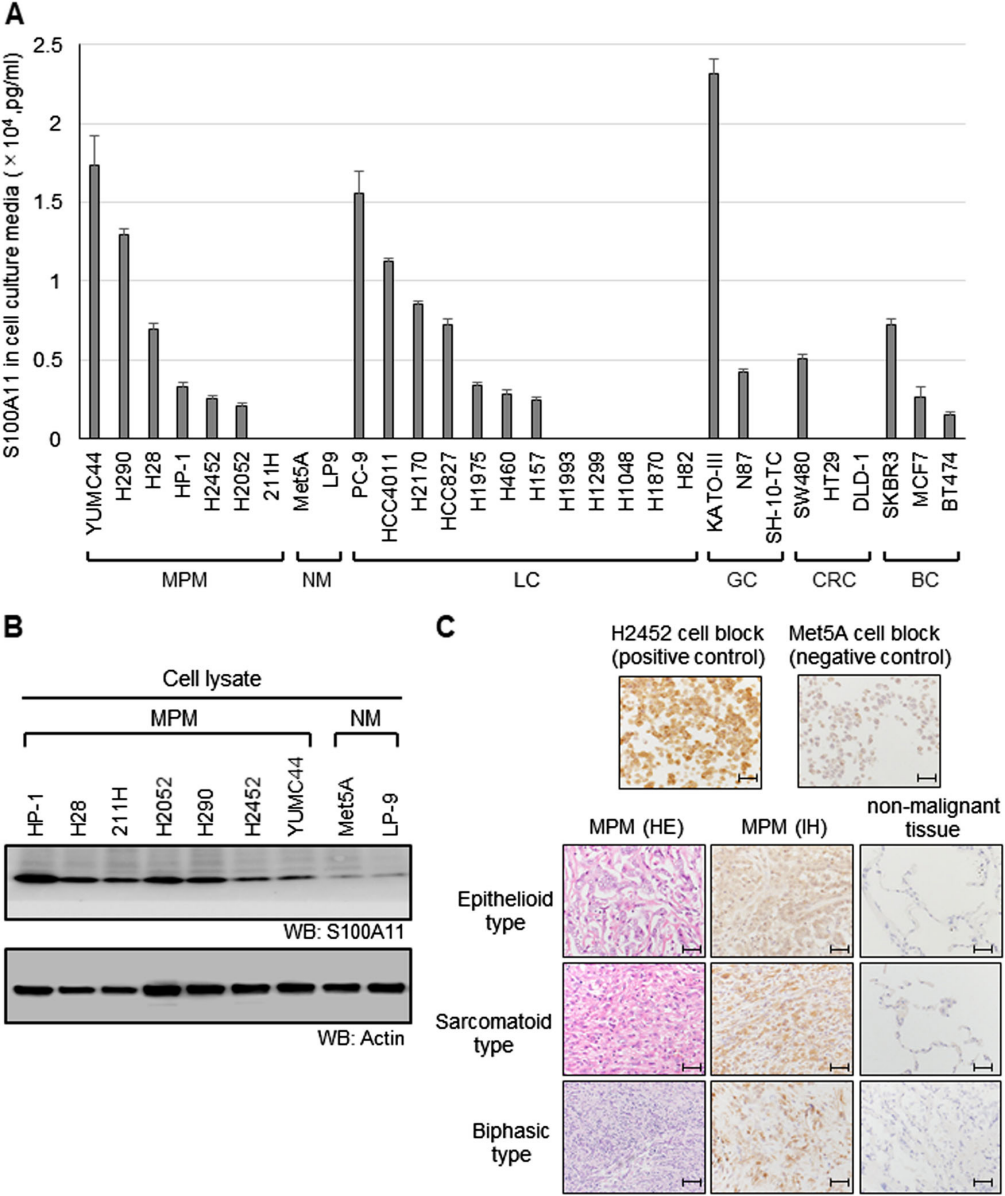
High secretion and overexpression of S100A11 in MPM. **a** Concentration of secreted S100A11 in the culture media of various cell lines, as determined by ELISA. The secretion level of S100A11 in all MPM cell lines, except for 211H, is higher than that of NM. 211H MSTO-211H, NM normal mesothelial cell, LC lung cancer, GC gastric cancer, CRC colorectal cancer, BC breast cancer. **b** Protein level of S100A11 was higher in MPM cell lines, compared to NM. NM normal mesothelial cell. **c** Immunohistochemical analysis of S100A11 in surgically resected tissues from patients with MPM. The representative images of three MPM subtypes are shown. The pictures on the left show hematoxylin–eosin (HE)-stained images corresponding to the S100A11-stained images in the middle. Scale bars, 50 µm (positive and negative control) and 100 µm (clinical samples)

We first examined the secretion level of S100A11 in the culture media of various cell lines by enzyme-linked immunosorbent assay (ELISA). Seven MPM, 2 normal mesothelial, 12 lung cancer, 3 gastric cancer, 3 colorectal cancer, and 3 breast cancer cell lines were used for this analysis, and the result is shown in Fig. 1a. We detected increased levels of S100A11 in cancer cells with various secretion levels. Of interest, there was the marked difference in S100A11 secretion between MPM cells and normal cells. All examined MPM cell lines except for MSTO-211H commonly secreted S100A11, whereas no secretion was observed in normal mesothelial cell lines. MPM cell lines were classified into three categories based on the secretion level of S100A11: High (YUMC44, H290, and H28), Low (HP-1, H2452, and H2052), and None (MSTO-211H). To investigate the correlation between S100A11 secretion and protein expression, protein expression levels of S100A11 in MPM and normal mesothelial cell lines were determined by western blot analysis. S100A11 was significantly overexpressed in MPM cell lines, compared to normal mesothelial cell lines (Fig. 1b). To confirm the same phenomenon in clinical MPM specimens, three histological subtypes of MPM tissues, epithelioid, sarcomatoid, and biphasic type, were obtained from the patients who underwent surgery at the Okayama University Hospital and then studied for S100A11 expression. We also prepared the paraffin blocks filled with H2452 and MeT-5A cells to use as a positive and negative control, respectively. Immunohistochemistry demonstrated that S100A11 was mainly localized in the cytoplasm or nucleus and was strongly positive in MPM cells, but not in surrounding normal lung cells. Representative images are shown in Fig. 1c. Taken together, these results suggest that S100A11 is aberrantly overexpressed in MPM at both cultured cells and clinical samples and secreted from MPM cells.

### Inhibition of the amount of extracellular S100A11 in MPM cells

**Fig. 2:**
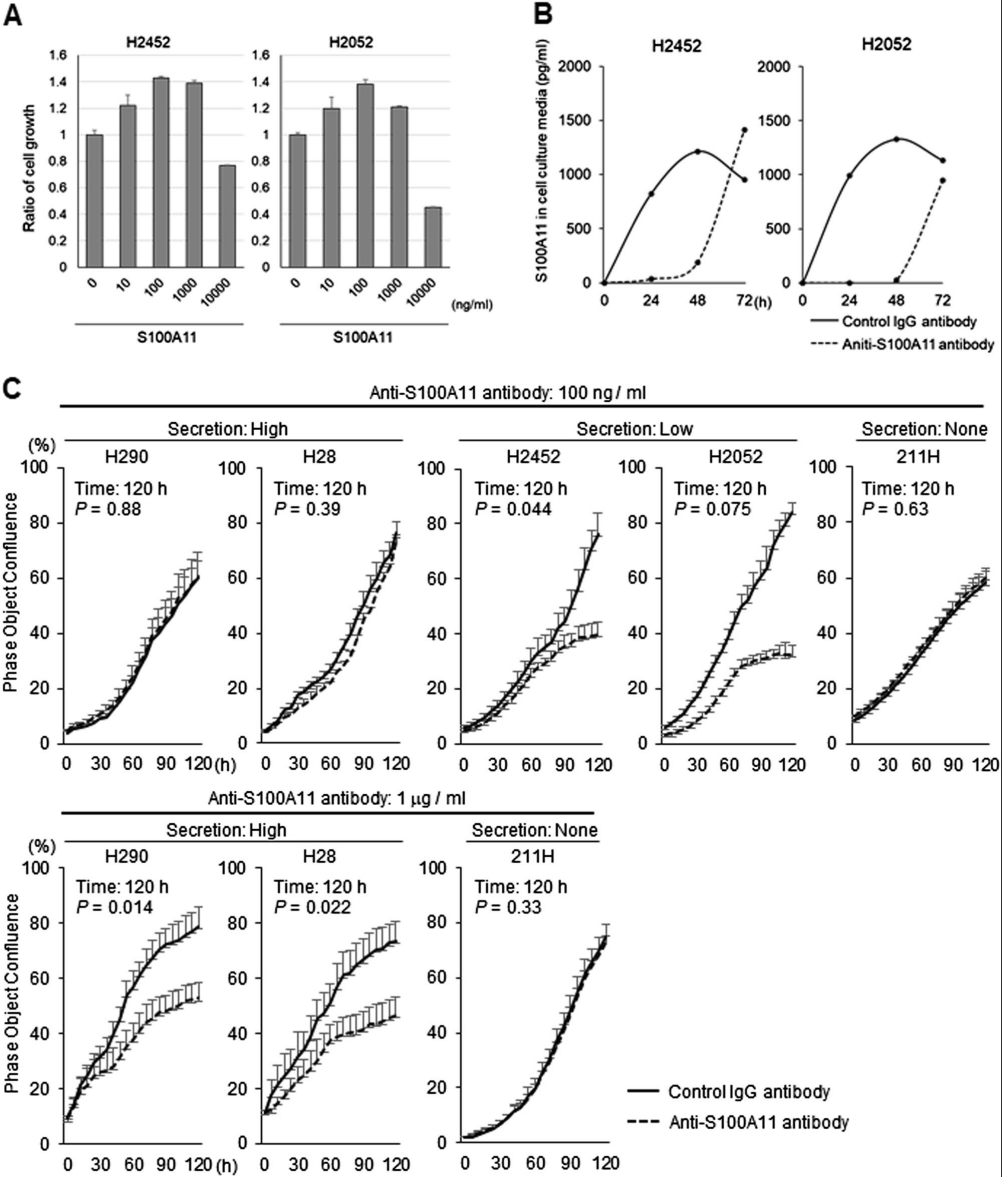
Suppression of MPM cell growth through neutralization of extracellular S100A11. **a** Effect of S100A11 recombinant protein on cell growth. Cells were treated or not treated with the foreign S100A11 protein at the indicated concentrations for 48 h. MTT assay was used for the following growth evaluation. **b** Effect of an anti-S100A11 antibody on the concentration of S100A11 in cell culture media. The antibody was added to the culture media of MPM cells (H2452 and H2052) at time 0. The concentration of S100A11 was significantly lower in the anti-S100A11 antibody-treated group, indicating that the specific antibody can neutralize secreted S100A11. **c** Effect of neutralization of extracellular S100A11 on cell growth in MPM cells. An anti-S100A11 antibody significantly inhibited cell growth in H2052, H2452, H290, and H28 cells (100 ng/ml antibody for H2052 and H2452 and 1 μg/ml for H290 and H28, respectively)

Next, we examined the involvement of extracellular S100A11 in the growth regulation of MPM cells. As shown in Fig. 2a, MTT assay revealed that administration of the purified S100A11 recombinant protein to the cultures promoted cellular proliferation of MPM cell lines (H2452 and H2052) in a dose-dependent manner up to the point of 100 ng/ml. The growth induction stimulated by extracellular foreign S100A11 at the concentration of 1000 ng/ml was almost equal in H2452 cells or tended to little bit decline in H2052 cells when compared to those at the concentration of 100 ng/ml. While the highest concentration (10,000 ng/ml) commonly worked as growth inhibition in the both cell lines. These results provided us an optimal concentration of extracellular S100A11 (100 ng/ml) in growth stimulation of MPM cells. To inhibit the function of secreted S100A11, we used an anti-S100A11 antibody (Proteintech Group), which has been previously reported as a neutralizing antibody for S100A11^13^. Similarly, we confirmed that the screened antibody decreased the quantity of S100A11 in the cell culture media (Fig. 2b). The effect was continued for about 48 h and then expired at later time point, probably due to the consumption of the antibody life. We also confirmed that moues control IgG had no effect on cell growth of MPM cell lines (H2052 and H2452, data not shown). Based on these results, the anti-S100A11 antibody was administered on day 1 and day 3 in culture system. We found that cellular growth of “Low” cells (H2452 and H2052) was both highly suppressed by an anti-S100A11 antibody with a final concentration of 100 ng/ml in culture media (Fig. 2c, top). On the other hand, in “High” cells (H290 and H28), the same concentration of the anti-S100A11 antibody did not show any appreciable effect on their cell growth. Facing to the issue, we increased the antibody dose up to 1 μg/ml. The higher dose gave suppression of cell growth even in these cell lines (Fig. 2c, bottom). As for “None” cells (MSTO-211H), the antibody did not affect the cell growth, regardless of dose (Fig. 2c). To further evaluate the effect of anti-S100A11 antibody on the migration and invasion of MPM, we performed a Boyden chamber assay. Microscopy images of the Boyden chamber assay are shown in Supplementary Fig. S1A and S1B. Migration and invasion were significantly suppressed in MPM cells treated with the antibody. To confirm the used antibody specificity, we tried to assess whether replenishing foreign S100A11 protein in the culture could cancel the antibody-mediated anticancer behaviors in vitro. As shown in Supplementary Fig. S1A–C, we found that this procedure effectively enfeebled cancer preventive benefits of the antibody in all the three experimental contexts, migration, invasion, and growth of MPM cell lines (H2452 and H2052).

There is a close relationship between cellular growth and cell cycle condition. This prompted us to examine whether cell cycle can be affected by the extracellular S100A11. Cyclins D and B were reliable good makers for cell cycle G1/S-phase and G2/M-phase, respectively, so that we investigated the expression levels of them after stimulation of the cells with 100 ng/ml of S100A11. By the western blot analysis approach, we found that Cyclin D but not Cyclin B was significantly induced with time dependency after the addition of S100A11 in MPM cultures (H2452 and H2052) (Fig. S1D). In addition, we found that anti-apoptotic Bcl-2 protein was significantly elevated with a similar manner to those of Cyclin D in both H2452 and H2052 cells (Fig. S1D). These results suggest that the secreted S100A11 positively regulates not only cell cycle, especially in a specific activation of the G1 phase and following G1/S transition through an induction of Cyclin D, but also survival via induction of Bcl-2.

### The alteration of downstream signaling affected by the anti-S100A11 antibody and the S100A11 recombinant protein

To gain insight into the intracellular signaling events induced by the neutralization of extracellular S100A11, we investigated the key molecules regarding to cancer progression. MPM cells were cultured with the anti-S100A11 antibody (1 μg/ml), S100A11 recombinant protein (100 ng/ml), or mouse control IgG (1 μg/ml). Lysates were extracted every 6 h and subjected to western blot analysis (Supplementary Fig. S2). As a result, we found that the treatment with the anti-S100A11 antibody significantly suppressed the constitutive phosphorylation of endogenous STAT3 in H2452 and H2052 cells. Unexpectedly, in cases of AKT and MAPK, both phosphorylation status were commonly upregulated temporary at once at 6 h and then tended to be gradually suppressed with a time-dependent manner (Supplementary Fig. S2A). This may be possibly explained by compensation of the STAT3 downregulation. Next as a converse experiment, we stimulated the cells with extra recombinant S100A11 under the absence of the antibody in culture. The addition of S100A11 recombinant protein in turn activated the downstream proteins, STAT3, AKT, and MAPK (Supplementary Fig. S2B). To further specify the central pathway related to the extracellular S100A11-induced proliferation of MPM cells, we tried to inhibit the intracellular activations of STAT3, MEK, and PI3K and assessed the following cellular proliferations. Unexpectedly, we found that either Stattic: STAT3 inhibitor, Trametinib: MEK inhibitor, or Taselisib: PI3K inhibitor, effectively suppressed the S100A11-stimulated cellular proliferation with a similar inhibitory degree, suggesting that these molecules play a crucial role in cellular growth and they cooperatively function toward same way regarding growth upregulation under the downstream of RAGE upon S100A11 binding (Supplementary Fig. S2D). Taken together, these results strongly suggest that the secreted S100A11 plays a critical role on MPM progression through upregulation of cell growth, motility, and invasion with an autocrine manner and the extracellular S100A11 is becoming a prominent molecule for therapeutic target of the MPM progression.

### An anti-S100A11 antibody inhibits the tumorigenic potential in a mouse xenograft model of MPM

**Fig. 3:**
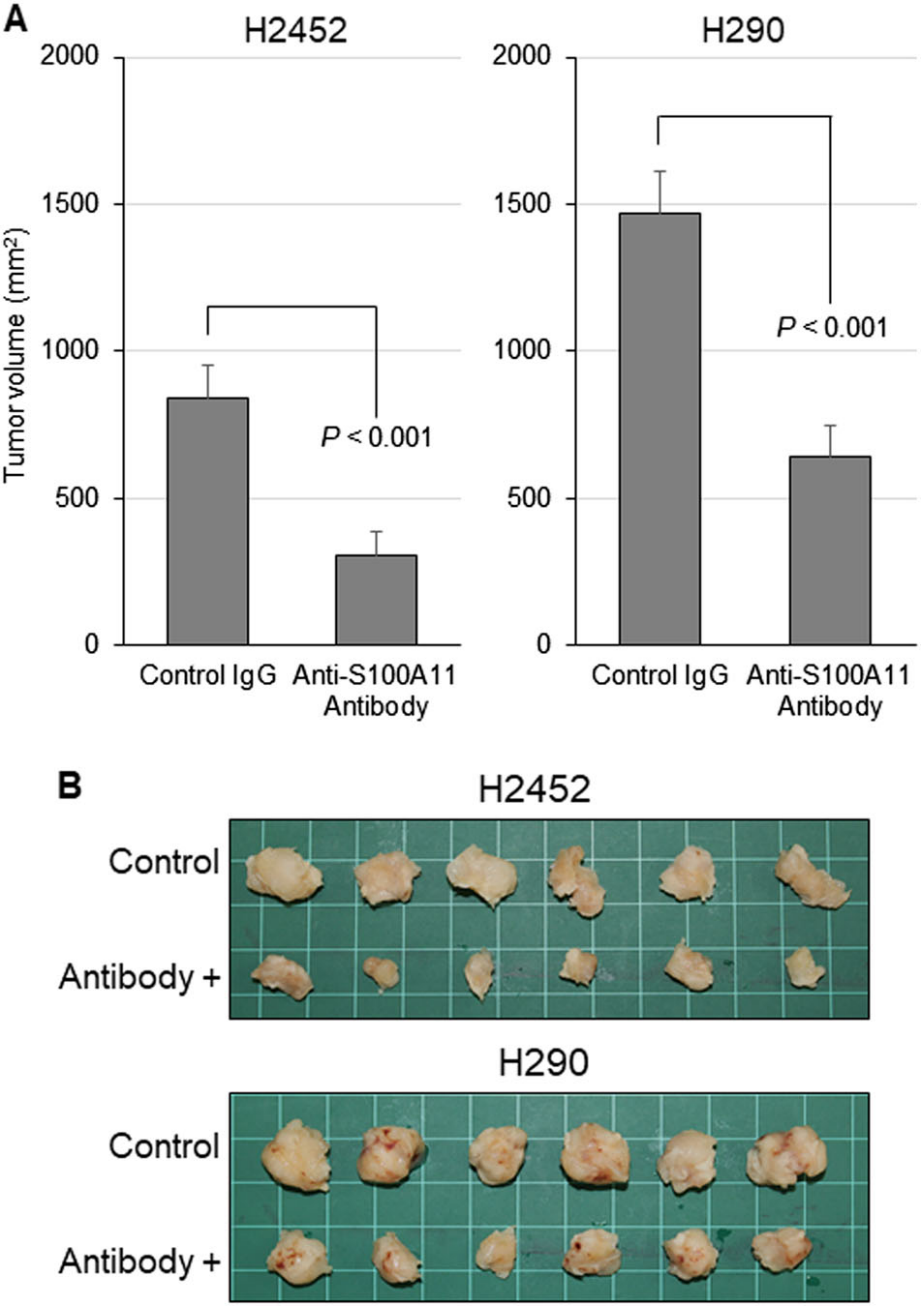
Negative effect of the treatment with an anti-S100A11 antibody on MPM tumor growth in vivo. **a** The mean volume of the subcutaneous xenograft tumors was calculated for six tumors in each group. The anti-S100A11 antibody significantly inhibits the tumorigenic potential in mouse xenografts of H2452 and H290 cells. **b** Appearance of the tumors at the time of sacrifice after treatment with control IgG or anti-S100A11 antibody. Tumors in the upper side of the photo were treated with mouse IgG control and tumors in the lower side were treated with an anti-S100A11 antibody

We investigated the antitumor effect of the validated anti-S100A11 neutralizing antibody on the MPM in a xenograft mouse model, using H2452 and H290 cells. We subcutaneously injected tumor cells and the anti-S100A11 antibody, and evaluated the effect of the antibody on the tumorigenic potential. As shown in Fig. 3a, b, the tumor growth in the antibody-administered group was significantly suppressed, compared to those of the control group.

### Microarray analysis of H2052 and H2452 cells treated with an S100A11 neutralizing antibody

**Fig. 4:**
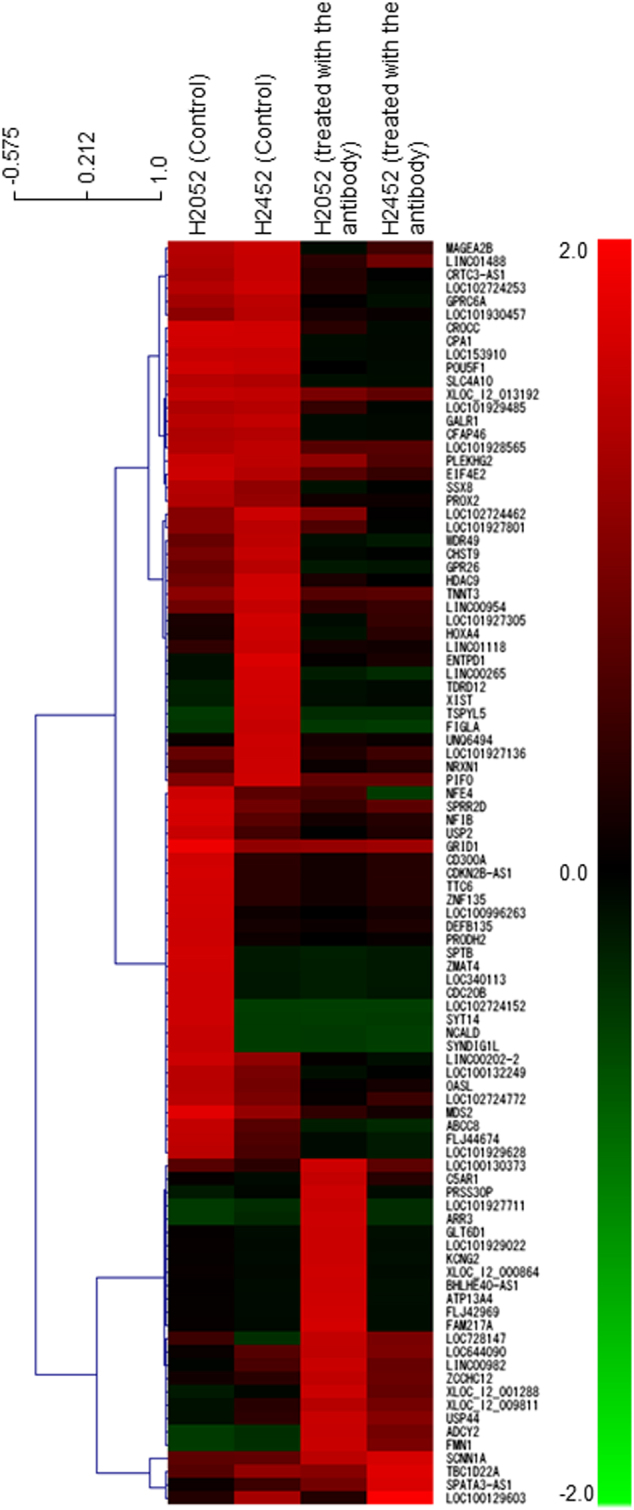
Global gene expression changes upon treatment with an anti-S100A11 antibody Clustering and heatmap of MPM cell lines are shown. Paired comparison analysis based on 50,739 probes indicated 2222 differentially expressed transcripts between antibody-treated MPM cell lines and parental MPM cell lines (H2052 and H2452). Clustering using Pearson correlation and average linkage was based on these transcripts. The color bar represents the expression values after log2 transformation

To reveal the mechanisms involved in the anticancer activity induced by neutralization of extracellular S100A11, we carried out gene expression microarray analysis. The details of the expression profile studies are reported in Supplementary Table S1. Clustering analysis based on the transcripts showed a clear distinction between the cell lines treated or untreated with the antibody (Fig. 4). Next, a gene set enrichment analysis (GSEA) was performed to clarify the groups of genes affected by anti-S100A11 antibody administration. We found that several pathways, including genes involved in cell proliferation (HALLMARK_MITOTIC_SPINDLE), (HALLMARK_P53_PATHWAY 1), (HALLMARK_PI3K_AKT_MTOR_SIGNALING), and (HALLMARK_KRAS_SIGNALING_DN), and a subgroup of genes related to protein secretion (HALLMARK_PROTEIN_SECRETION) were negatively enriched in the antibody-treated cell lines (Table 1). These results are consistent with our in vitro and in vivo data.

**Table 1:** Enriched pathways in the parental and antibody-treated cell lines

	ES	FDR *q*-val
GSEA pathway (parental cell line, *n* = 2)		
HALLMARK_PANCREAS_BETA_CELLS	0.83	0.274
HALLMARK_REACTIVE_OXIGEN_SPECIES_PATHWAY	0.6	0.274
HALLMARK_COAGULATION	0.52	1.000
HALLMARK_PEROXISOME	0.51	0.995
HALLMARK_MYC_TARGETS_V2	0.44	0.926
HALLMARK_GLYCOLYSIS	0.42	0.722
GSEA pathway (anti-S100A11 antibody-treated cell line, *n* = 2)		
HALLMARK_PI3K_AKT_MTOR_SIGNALING	−0.56	0.819
HALLMARK_PROTEIN_SECRETION	−0.47	0.47
HALLMARK_KRAS_SIGNALING_DN	−0.47	0.811
HALLMARK_MYOGENESIS	−0.47	0.87
HALLMARK_MITOTIC_SPINDLE	−0.33	0.887
HALLMARK_P53_PATHWAY	−0.35	0.409

*ES* enriched score, *FDR* false discovery rate

### Function of RAGE as a receptor for S100A11

**Fig. 5:**
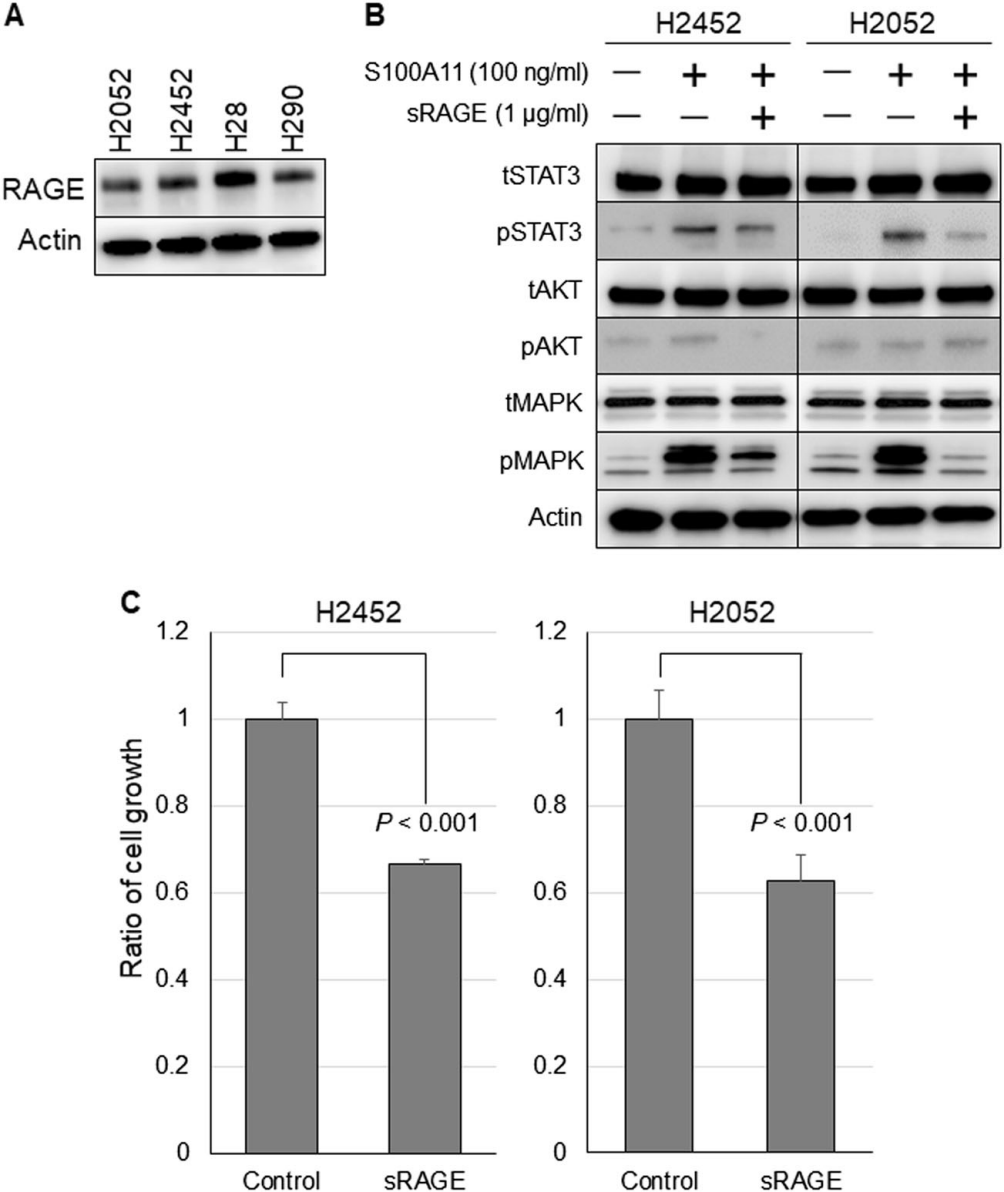
Secreted S100A11 exerts its important role in the proliferation of MPM cells via RAGE. **a** RAGE protein expression in MPM cells. **b** Inhibition of RAGE signaling through sRAGE significantly suppressed the phosphorylation of STAT3 and MAPK induced by S100A11 recombinant protein. **c** The effect of sRAGE on the growth of MPM cells examined by MTT assay. Cell proliferation was significantly inhibited by sRAGE

To explore the function of RAGE as a receptor for S100A11 in MPM, we first confirmed the positive expression of RAGE in MPM cell lines by western blot analysis (Fig. 5a). To prevent the intrinsic RAGE activation caused by ligand binding, we used sRAGE, which acts as decoy to compete with S100A11, the ligand for the cellular RAGE, and examined a series of effector molecules regarding to RAGE downstream signaling. MPM cells were treated or not treated with S100A11 recombinant protein (100 ng/ml) under the presence or absence of sRAGE (1 μg/ml). Twenty-four hours later, the treated-cell lysates were prepared and then subjected to western blot analysis. We found that blockage of RAGE signaling suppressed the phosphorylation of STAT3 and MAPK induced by S100A11 stimulation (Fig. 5b). Additionally, MTT assays revealed that sRAGE mitigated growth promotion in both H2452 and H2052 cells, indicating that S100A11–RAGE axis plays a pivotal role in MPM progression in response to the secreted extracellular S100A11 (Fig. 5c).

### Secretion levels of S100A11 in pleural effusions

**Fig. 6:**
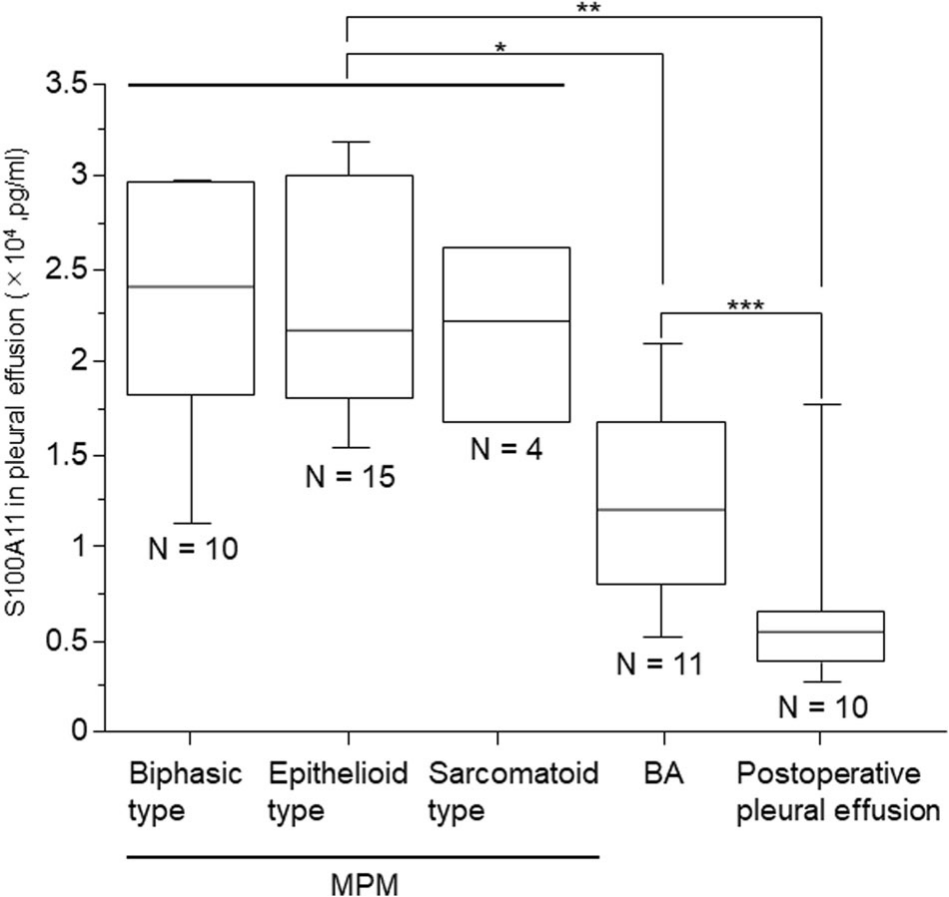
Secretion levels of S100A11 in pleural effusions, as determined by ELISA. Significant differences among the different types of pleural effusion were observed (*: MPM vs BA, *P* = 0.014, **: MPM vs postoperative pleural effusion, *P* < 0.001, ***: BA vs postoperative pleural effusion, *P* = 0.041)

To explore the possibility of using S100A11 as a useful marker for diagnosis of MPM, we examined the secretion levels of S100A11 in several types of pleural effusions. We obtained pleural effusions from 29 MPM patients (biphasic type: *n* = 10, epithelioid type: *n* = 15, sarcomatoid type: *n* = 4), 11 benign asbestosis (BA) patients, and 12 patients who underwent thoracic surgery at the Okayama University Hospital and the National Hospital Organization Yamaguchi-Ube Medical Center (Ube, Yamaguchi, Japan). Postoperative pleural effusions were obtained from lung cancer (*n* = 10) or pulmonary cyst (*n* = 2) patients after postoperative day 2. The concentration of S100A11 was measured by ELISA. As shown in Fig. 6, the level of S100A11 was remarkably elevated in pleural effusion obtained from MPM patients when compared to that obtained from BA patients as a benchmark (*P* = 0.014). Furthermore, we found that the secretion level of S100A11 was significantly higher in pleural effusion from BA patients than that from postoperative patients (*P* = 0.041).

## Discussion

In this study, we found that extracellular S100A11, via RAGE, has a critical role in tumor progression of MPM. The blockage of extracellular S100A11 with a neutralizing antibody inhibited the cell proliferation, migration, and invasion of MPM cells. Additionally, in a mouse xenograft model, tumorigenesis of MPM cells was markedly inhibited by an anti-S100A11 antibody. These results suggest that extracellular S100A11 is a potential therapeutic target for MPM. Interestingly, we found that protein expression levels of S100A11 are not correlated with secretion levels in various cells lines, suggesting that intracellular increasing in stock level of S100A11 protein is not essential factor for its secretory mechanism. In fact, MSTO-211H showed no secretion of S100A11 even though plenty of S100A11 protein was stored in cells. To clarify the reason of this discrepancy at the molecular level, further comprehensive investigation with focusing on secretory pathways of leaderless proteins is indispensable.

One of the interesting features of S100A11 is to possess different functions depending on its location and on different cell types, as we reported previously^9^. Accumulating evidence indicates that S100A11 expression is upregulated in various cancers and promote cancer development^14–17^. However, this is not common in all cancer species, for example, low expression of S100A11 in bladder cancers is associated with poor survival in the patients^18^. Therefore, S100A11 might act sometimes as an oncogene, and sometimes as a suppressor gene, and have a key role in the progression of malignant tumors based on the balance of its ambivalent effect, which may be different depending on cancer types. When considering the use of this protein for treatment, the targeting of intracellular S100A11 would require the unitarily control of its two different functions. Thus, it is more convenient to focus on extracellular S100A11 for developing new therapeutic strategies for MPM.

RAGE, which was first identified as a RAGE, is known to bind different ligands, such as amyloid-beta peptide, HMGB1 (amphoterin), and some members of the S100 family, including S100A11^19–21^. We found that RAGE-positive MPM cells constitutively express and secrete S100A11, the S100A11–RAGE axis is greatly involved in sustained phosphorylation of downstream effector molecules, STAT3, MAPK, and PI3K–AKT, and the blockage of S100A11–RAGE connection using either sRAGE or S100A11 neutralizing antibody effectively inhibits cell growth of MPM cells. For the S100A11-mediated growth upregulation, Cyclin D and Bcl-2 might be critically relevant. How are these molecules induced under the RAGE activation? One hint may come from the Fig. S2D. The S100A11-induced Cyclin D but not Bcl-2 was significantly downregulated by the STAT3, MEK and PI3K inhibitors, suggesting that the molecules regarding pathways coordinately regulate Cyclin D expression after the RAGE activation upon S100A11 binding. Through this study, we hence strongly recognized a significant role of secreted S100A11 in mesothelioma progression.

In our experiments, growth inhibitory effect of sRAGE was limited and insufficient in comparison with the that of anti-S100A11 antibody, indicating that an additional S100A11 receptor, such as CD36, might activate survival or proliferative pathways in the cells^9,22^. Moreover, RAGE has been shown to have many distinct biological functions. For instance, van Zoelen and colleagues^23^ demonstrated that RAGE signaling contributed to an effective antibacterial defense during *Escherichia coli* infection (inhibition of bacterial outgrowth and dissemination), and RAGE deficiency resulted in enhanced organ injuries due to liver necrosis. Thus, the adverse effects due to the inhibition of RAGE signaling are still unclear and as a therapeutic target for MPM, it seems more convenient to inhibit extracellular S100A11 than RAGE at present.

Although “High” cells (H290 and H28) displayed resistance to the S100A11 antibody at lower concentration (100 ng/ml), the effect of S100A11 antibody was shown when much higher concentration of it (1 μg/ml) was used. Thus, the secretion of S100A11 in MPM cells was prerequisite for the antibody approach and measurement of the secretion level of S100A11 was quite important index to predict an effectiveness of the S100A11 antibody. We hence examined the secretion level of S100A11 in pleural effusion. The concentration of S100A11 in MPM pleural effusions was significantly higher than that in pleural effusions of BA and postoperative patients, though the amount of secreted S100A11 is high in pleural effusions from both patients with MPM and BA. Generally, BA is not regarded as a precancerous lesion of mesothelioma. Taking it into account, we consider that MPM cells actively secrete S100A11 in pleural effusion. These results suggest that S100A11 can be not only a therapeutic target but also an early diagnosis marker. In addition, it is of note mentioning that the concentration of S100A11 in the pleural effusions of patients with BA was significantly higher than that of postoperative patients. The association between high expression of proteins of the S100 protein family and inflammatory diseases has been reported^24–27^; this along with our results suggest that S100A11 may be involved in chronic inflammation.

In conclusion, we showed that extracellular S100A11 plays an important role in MPM progression and neutralization of extracellular S100A11 directly linked to marked suppression of MPM developments in vitro and in vivo. Our results suggest that extracellular S100A11 can be a novel, selective and effective therapeutic target in S100A11-secreting MPM, since normal cells displayed no secretion phenotype of S100A11.

## Materials and methods

### Cell lines and reagents

We used seven human MPM cell lines [NCI-H28 (H28), NCI-H290 (H290), NCI-H2052 (H2052), NCI-H2452 (H2452), HP-1, YUMC44, and MSTO-211H], two human normal mesothelial cell lines (MeT-5A and LP-9), 10 human lung cancer cell lines [HCC827, PC-9, HCC4011, NCI-H1975 (H1975), NCI-H1993 (H1993), NCI-H1299 (H1299), NCI-H460 (H460), NCI-H2170 (H2170), NCI-H157 (H157), and NCI-H82 (H82)], two human extrapulmonary small-cell cancer cell lines [NCI-H1048 (H1048) and NCI-H1870 (H1870)], three gastric cancer cell lines [KATO III, NCI-N87 (N87), and SH-10-TC], three colorectal cancer cell lines (SW480, HT29, and DLD-1), and three breast cancer cell lines (MCF-7, BT474, and SK-BR-3). Two MPM cell lines (HP-1 and H2452) were established by one of the authors (H.I.P.)^28^. Three MPM cell lines (H28, H290, and H2052 obtained in 2002), two lung cancer cell lines (HCC4011 and H82 obtained in 2008), and two extrapulmonary small-cell cancer cell lines (H1048 and H1870 obtained in 2008) were kindly gifted by Dr. Adi F. Gazdar (Hamon Center for Therapeutic Oncology Research and Department of Pathology, University of Texas Southwestern Medical Center at Dallas, Dallas, TX, USA)^29^. YUMC44 cell line was established by one of the authors (H.Y.)^30^. MSTO-211H, MeT-5A, N87 cell lines, seven lung cancer cell lines (HCC827, H1975, H1993, H1299, H460, H2170, and H157), three breast cancer cell lines (MCF-7, BT474, and SK-BR-3), and three colorectal cancer cell lines (Sw480, HT29, and DLD-1) were purchased from the American Type Culture Collection (Manassas, VA, USA). Two gastric cancer cell lines (KATO III and SH-10-TC) were obtained from the Cell Resource Center for Biomedical Research Institute of Development Aging and Cancer, Tohoku University (Sendai, Miyagi, Japan). LP-9 cell line was purchased from the Coriell Cell Repository (Camden, NJ, USA). PC-9 cell line was purchased from Riken Cell Bank (Tsukuba, Ibaragi, Japan). The cell lines within 30 passages were used in this study and the cumulative culture length was less than 6 months. For cell lines with long-term preservation in liquid nitrogen, DNA fingerprinting analysis by short tandem repeat profiling (the PowerPlex 1.2 System, Promega) was performed for the cell authentication. All the cell lines, except for LP-9 and breast cancer cell lines, were maintained in RPMI-1640 medium (Sigma–Aldrich, St Louis, MO, USA, Product No. R8758) supplemented with 10% fetal bovine serum (FBS). The three breast cancer cell lines were cultured in Dulbecco’s modified Eagle’s medium (Sigma–Aldrich, Product No. D6429) with 10% FBS. LP-9 was cultured in Ham’s F12 medium (Sigma–Aldrich, Product No. N4888)/Medium 199 (Sigma–Aldrich, Product No. M7653) (1:1 mixture) with 10% FBS, 2 mM l-glutamine (Thermo Fisher Scientific, Waltham, MA, USA, catalog #25030-081), 1.7 nM epidermal growth factor (Sigma–Aldrich, Product No. E9644), and 1100 nM hydrocortisone (Sigma–Aldrich, Product No. H0888). All the cell lines were grown in a humidified incubator with 5% CO_2_ at 37°C and routinely tested for mycoplasma by Venor GeM OneStep kit (Minerva Biolabs, Berlin, Germany, Product No. 11-8050). Soluble receptor for advanced glycation end products (RAGE) Fc chimera (sRAGE) (R&D Systems, Minneapolis, MN, USA, accession #Q15109), static (Abcam, Cambridge, MA, USA, ab120952), trametinib (GSK-1120212) (LC Laboratories, Woburn, MA, USA, catalog #T-8132), Taselisib (GDC-0032) (Selleckchem, Houston, TX, USA, catalog #S7103), and the mouse IgG-isotype control (Abcam, ab37355) were obtained from the designated sources. S100A11 recombinant protein was prepared as described previously^5^.

### Determination of the concentration of S100A11 by ELISA

The concentration of S100A11 was measured using the CircuLex S100A11 ELISA Kit (Circulex, Nagano, Japan, Code No. CY-8063) according to the manufacturer’s protocol. The standard curve for the ELISA was obtained using various concentrations of recombinant human S100A11. Samples were diluted 5- to 10-fold. The absorbance was measured at the dual wavelengths of 450/540 nm using the Flex Station 3 microplate reader (Sunnyvale, CA, USA) and the concentration of S100A11 (in pg/ml) was calculated according to the standard curve.

### Western blot analysis

The detailed protocol has been described previously^31^. The primary antibodies used for western blot analyses were as follows: EGFR (catalog #4267), phospho- (p-)EGFR (Tyr1068) (#3777), Stat3 (#12640), p-Stat3 (Tyr705) (#9145), Akt (#9272), p-Akt (Ser473) (#4060), p44/p42 MAPK (#9102), p-p44 ⁄ p42 MAPK (#9101), Cyclin B1 (#4138), Cyclin D1 (#2922), Bcl-2 (#2872) (Cell Signaling Technology, Danvers, MA, USA), S100A11 (Medical & Biological Laboratories, Nagoya, Japan, catalog #CY-M1037), and β-actin (used as a loading control) (Merck Millipore, Billerica, MA, USA, catalog # MAB1501). The following secondary antibodies were used: goat anti-rabbit (catalog #sc-2030) or anti-mouse (catalog #sc-2031) immunoglobulin G (IgG)-conjugated horseradish peroxidase (Santa Cruz Biotechnology, Dallas, TX, USA). To detect specific signals, the membranes were examined using the ECL Prime Western Blotting Detection System (GE Healthcare, Amersham, UK, Code No. RPN2235) and LAS-3000 imager (Fujifilm, Tokyo, Japan).

### Immunohistochemical analysis of clinical samples

MPM tissues were obtained from patients who underwent surgery at the Okayama University Hospital, in Okayama City, Japan. The experimental protocol was approved by the Institutional Review Board/Ethical Committee of the Okayama University Graduate School of Medicine, Dentistry and Pharmaceutical Sciences and Okayama University Hospital (Permit Number: 1508-027) and informed consent, including publication of patient photos, was obtained from all the patients. Tissue samples were fixed in 10% formaldehyde and embedded in paraffin. The immunohistochemical (IHC) staining for S100A11 was performed using an S100A11 primary antibody (R&D Systems, accession #P31949). The detailed protocol for the IHC staining has been described previously^32^.

### Cell proliferation assays

To examine the effect of the anti-S100A11 antibody (Proteintech Group, Chicago, IL, USA, catalog #10237-1-AP) on cell proliferation, the IncuCyte Zoom Live Cell Imaging System (Essen Biosciences, Ann Arbor, Michigan, USA) was used. In brief, 1.0 × 10^4^ cells were seeded into each well of a 24-well plate. After 24 h of incubation, the plates were placed in the Incucyte Zoom system. Images were taken every 6 h for the indicated amount of time. The percent of confluence over time was calculated using the Incucyte Zoom software. For experiments testing the effect of sRAGE on cell proliferation, the thiazolyl blue tetrazolium bromide (MTT) (Sigma–Aldrich, catalog #M2128) dye reduction method was used, as described previously^33^.

### Cell migration and invasion assays

Cell migration and invasion were analyzed using a Boyden chamber assay. The cells were cultured with an antibody (100 ng/ml) for 24–48 h (for migration assays) or 48–72 h (for invasion assays). The detailed protocol has been described previously^34^.

### Xenograft model

The protocol was approved by the Animal Care and Use Committee of Okayama University (Permit Number: OKU-2016205). Six-week-old NOD⁄SCID female mice were purchased from Charles River Laboratories (Yokohama, Kanagawa, Japan). To evaluate the effect of the anti-S100A11 neutralizing antibody, mice were randomly divided into two groups: an antibody-administered group and a control group (*n* = 3 for each group). Each cell line (5 × 10^6^ cells) suspended in 200 µL RPMI-1640 media and Matrigel Basement Membrane Matrix (Corning, NY, USA, catalog #354234) was subcutaneously injected into the backs of the mice either with 1 µg/ml anti-S100A11 antibody (proteintech Group) or mouse IgG-isotype control (two tumors per mouse). The injection was repeated five times every 3 days. Four weeks after the first injection, the mice were sacrificed and the tumors were harvested, measured, and photographed. The tumor volume was calculated using the empirical formula *V* = 1/2 × [(shortest diameter)^2^ × (the longest diameter)]. No randomization and no blinding was performed. Sample size estimates were based on our previous experience.

### Microarray and GSEA

Before and after treatment of H2052 and H2452 cells with the anti-S100A11 antibody (Proteintech Group), total RNAs were extracted from cell lines using an RNeasy Mini Kit (Qiagen, Venlo, the Netherlands, catalog #74104). Purified total RNA samples were hybridized on the Human Whole Genome DNA Microarray system (SurePrint G3 Human 8x60K ver. 2.0, Agilent Technologies, Santa Clara, CA) to obtain the altered gene expression profile. The fold change in the expression of individual genes was calculated and genes with fold changes exceeding 2-fold or below 2-fold were considered up- and downregulated, respectively (Supplementary Table S1). The specific enrichment of gene sets was further analyzed using the GSEA software (GSEA ver. 2.0) downloaded from the GSEA Website (http://software.broadinstitute.org/gsea/index.jsp).

### Statistical analyses

All in vitro experiments were performed at least three times. Data are expressed as the mean±standard deviation. All data were analyzed using the JMP^®^ 9.0.0 software for Windows (SAS Institute, Inc., Cary, NC, USA). The Student’s *t*-tests was used to compare means of continuous scores between two independent groups. Otherwise, the Mann–Whitney *U*-test was used. An *F*-test to compare variances was performed, if necessary. All statistical tests were two-sided, and probability values less than 0.05 indicated statistically significant differences.

## Electronic supplementary material

Supplementary Figure S1

Supplementary Figure S2

Supplementary Figure legends

Supplementary table
