# Supplementary figures and images for: Therapeutic potential of targeting S100A11 in malignant pleural mesothelioma
Source: Oncogenesis. 2018 Jan 24;7(1):11. doi: 10.1038/s41389-017-0017-3 (PMC5833371; doi:10.1038/s41389-017-0017-3)

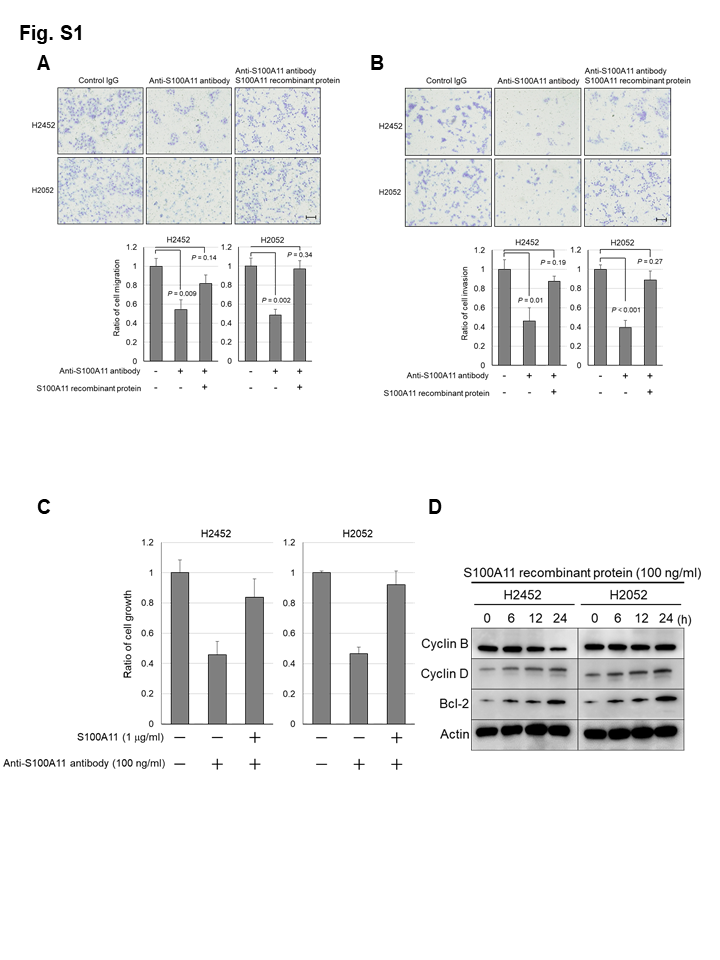

Supplement: Supplementary file 1 — Supplementary Figure S1 [file 41389_2017_17_MOESM1_ESM.tif]

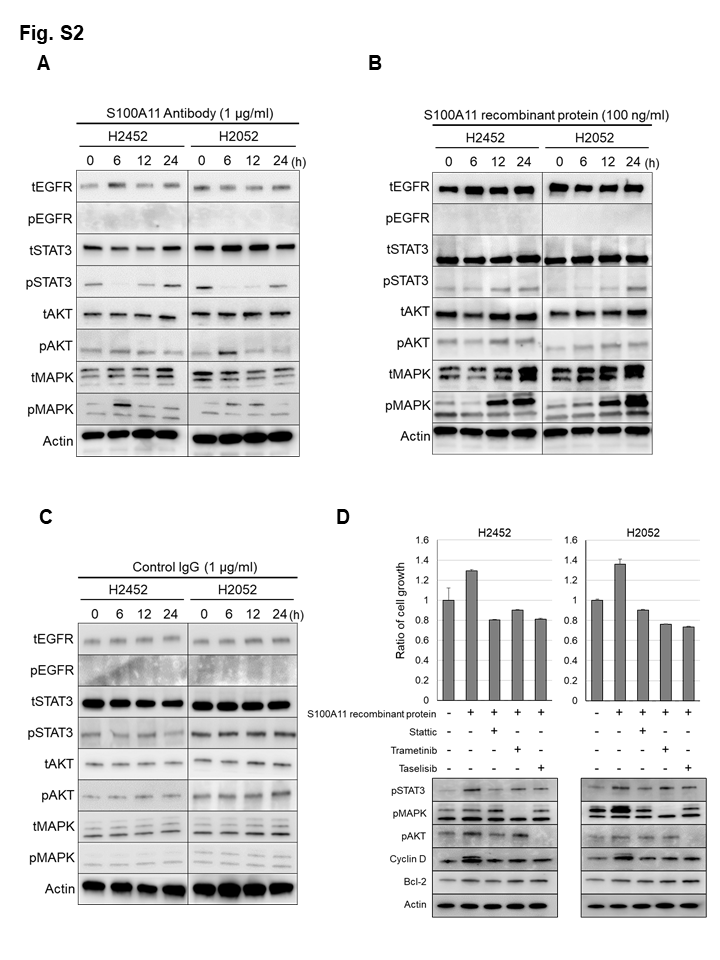

Supplement: Supplementary file 2 — Supplementary Figure S2 [file 41389_2017_17_MOESM2_ESM.tif]
