# Supplementary material for: Therapeutic potential of targeting S100A11 in malignant pleural mesothelioma
Source: Oncogenesis. 2018 Jan 24;7(1):11. doi: 10.1038/s41389-017-0017-3 (PMC5833371; doi:10.1038/s41389-017-0017-3)
Supplement: Supplementary file 3 — Supplementary Figure legends [file 41389_2017_17_MOESM3_ESM.docx]

**Supplementary figure legends**

**Fig. S1. Suppression of MPM cell migration and invasion by neutralization of extracellular S100A11**

(A and B) Effect of the anti-S100A11 antibody on MPM cell migration and invasion was tested using an antibody concentration of 100 ng/ml. At this time, we also assessed the antibody specificity by addition of the recombinant S100A11 (1 μg/ml) in the same culture including the antibody to block the antibody-mediated neutralization of cancer-secreted intrinsic S100A11. For the quantification of migration and invasion, the cells were counted under a microscope in five predetermined fields (magnification ×100). The anti-S100A11 antibody significantly inhibited cell migration (A) and invasion (B) of the MPM cell lines. These phenomena were canceled by co-existence with recombinant S100A11. Representative images are shown. Scale bars, 100 µm.

(C) Effect of the anti-S100A11 antibody (100 ng/ml) on cell growth was examined in the context of cell culture. Cells were treated with the antibody every 2 days and the cell growth was evaluated by MTT assay after 96 hours. The cellular proliferation was significantly mitigated by the presence of the antibody in culture medium and the suppression was canceled by the co-presence of the foreign S100A11 (1 μg/ml) with the antibody in culture medium.

(D) Expressions of cyclin B, D and Bcl-2 were examined by western blot analysis. Cells were treated or not treated with S100A11 recombinant protein at the concentration of 100 ng/ml for the indicated times.

**Fig. S2. The alteration of downstream signaling affected by the anti-S100A11 antibody and the S100A11 recombinant protein**

Extracellular S100A11 activated downstream proteins, whereas anti-S100A11 antibody inhibited them. (A) The alteration of downstream signaling caused by the anti-S100A11 antibody. (B) The alteration of downstream signaling caused by S100A11 recombinant protein. (C) The alteration of downstream signaling caused by mouse control IgG, which was used as a negative control.

(D) Effects of STAT3, MEK, and PI3K inhibitor on MPM cell proliferation were tested by MTT assay. Cells were treated with each specific inhibitor (1 μM) for 48 hours. Absorbance of the untreated cells was set at 1. The inhibitory functions of the used drugs were confirmed by western blot analysis to know the phosphorylation status of the indicated molecules. Expression levels of cyclin D and Bcl-2 were also examined by western blot analysis.
