# Supplementary material for: Therapeutic potential of targeting S100A11 in malignant pleural mesothelioma
Source: Oncogenesis. 2018 Jan 24;7(1):11. doi: 10.1038/s41389-017-0017-3 (PMC5833371; doi:10.1038/s41389-017-0017-3)
Supplement: Supplementary file 4 — Supplementary table [file 41389_2017_17_MOESM4_ESM.docx]

Table S1. Results of expression profiling analysis

(A) Up-regulated genes in H2052 treated by anti-S100A11 antibody compared to parental H2052.

| **ProbeName** | **GeneSymbol** | **Fold change** |
| --- | --- | --- |
| A_24_P344976 | ARR3 | 5.009 |
| A_33_P3379881 | FMN1 | 4.868 |
| A_21_P0001439 | LOC101927711 | 4.818 |
| A_32_P405973 | ADCY2 | 4.686 |
| A_21_P0000889 | DACT3-AS1 | 4.584 |
| A_23_P350719 | PRSS30P | 4.510 |
| A_33_P3367037 | lnc-SDR42E1-1 | 4.505 |
| A_33_P3766913 | DEFA10P | 4.492 |
| A_21_P0002151 | LOC101930516 | 4.488 |
| A_23_P47340 | DSCAML1 | 4.475 |
| A_21_P0010667 | XLOC_l2_001288 | 4.465 |
| A_33_P3357182 | FAM217A | 4.408 |
| A_21_P0008422 | lnc-MBIP-1 | 4.387 |
| A_21_P0008104 | lnc-GPC6-1 | 4.302 |
| A_23_P412508 | PDZD9 | 4.291 |
| A_23_P93727 | SDK1 | 4.258 |
| A_32_P169114 | GRIN2A | 4.240 |
| A_23_P143694 | SOX10 | 4.235 |
| A_33_P3328450 | HMX1 | 4.200 |
| A_33_P3534905 | FLJ42969 | 4.187 |
| A_21_P0005779 | lnc-RHPN1-4 | 4.183 |
| A_33_P3877992 | LOC286190 | 4.166 |
| A_33_P3283147 | OR5M11 | 4.164 |
| A_21_P0003688 | lnc-RP11-389E17.1.1-3 | 4.150 |
| A_23_P430948 | ATP13A4 | 4.150 |
| A_23_P72770 | USP44 | 4.140 |
| A_21_P0000913 | LOC101926944 | 4.138 |
| A_21_P0000706 | BHLHE40-AS1 | 4.115 |
| A_33_P3366376 | KRTAP27-1 | 4.112 |
| A_19_P00802137 | XLOC_l2_000864 | 4.109 |
| A_21_P0004668 | lnc-GJA10-1 | 4.101 |
| A_21_P0001091 | LINC00982 | 4.098 |
| A_33_P3303309 | LINC01105 | 4.097 |
| A_21_P0001620 | lnc-BATF3-2 | 4.067 |
| A_33_P3230748 | lnc-CEACAM18-2 | 4.051 |
| A_24_P52887 | ENDOU | 4.040 |
| A_23_P258136 | MXRA5 | 4.017 |
| A_33_P3264193 | OR2D2 | 4.013 |
| A_32_P4985 | CAMTA1 | 4.007 |
| A_23_P61171 | KCNG2 | 3.986 |
| A_33_P3281577 | BTN1A1 | 3.977 |
| A_21_P0005563 | LOC101929022 | 3.964 |
| A_33_P3320817 | GLT6D1 | 3.956 |
| A_23_P139099 | OR4A15 | 3.912 |
| A_33_P3374165 | lnc-FBXW11-3 | 3.902 |
| A_21_P0002246 | lnc-CTNNA2-2 | 3.897 |
| A_21_P0014339 | lnc-AC009113.1-1 | 3.877 |
| A_21_P0005203 | LOC100507642 | 3.872 |
| A_33_P3369944 | ADAM21 | 3.861 |
| A_21_P0004353 | LOC100506858 | 3.848 |
| A_21_P0007968 | LINC00354 | 3.835 |
| A_24_P634768 | FLJ22763 | 3.830 |
| A_33_P3411328 | LINC01136 | 3.828 |
| A_21_P0001924 | lnc-ACVR2A-1 | 3.828 |
| A_21_P0000932 | lnc-ALG14-5 | 3.821 |
| A_21_P0001643 | lnc-IRF2BP2-2 | 3.814 |
| A_24_P265832 | SUCNR1 | 3.798 |
| A_33_P3235177 | TET3 | 3.797 |
| A_24_P940275 | FRMPD4 | 3.788 |
| A_21_P0005921 | lnc-DLGAP2-2 | 3.786 |
| A_21_P0008006 | lnc-RBM26-3 | 3.747 |
| A_21_P0004428 | lnc-NUDT12-3 | 3.738 |
| A_23_P153562 | C5AR1 | 3.723 |
| A_21_P0007111 | lnc-ATAD1-1 | 3.698 |
| A_33_P3230663 | LOC100130232 | 3.671 |
| A_32_P358115 | LOC284648 | 3.670 |
| A_21_P0010020 | lnc-THBD-1 | 3.668 |
| A_23_P45592 | TTTY8 | 3.651 |
| A_33_P3259929 | LINC01561 | 3.650 |
| A_23_P256581 | PRDM13 | 3.643 |
| A_23_P170888 | DPP6 | 3.639 |
| A_21_P0000558 | BRE-AS1 | 3.622 |
| A_33_P3284734 | BEAN1 | 3.620 |
| A_21_P0004923 | lnc-RFPL4B-3 | 3.612 |
| A_33_P3212192 | CHRNG | 3.600 |
| A_23_P157593 | CHRNA6 | 3.591 |
| A_21_P0009975 | lnc-MOCS3-3 | 3.543 |
| A_21_P0005130 | lnc-TRMT11-1 | 3.539 |
| A_23_P155786 | SULT1E1 | 3.538 |
| A_33_P3345534 | KRT14 | 3.537 |
| A_23_P2283 | TAC3 | 3.534 |
| A_33_P3441031 | LINC00944 | 3.526 |
| A_21_P0004945 | lnc-TNFAIP3-6 | 3.508 |
| A_33_P3393200 | SRRM4 | 3.501 |
| A_21_P0008593 | lnc-C15orf2-5 | 3.499 |
| A_21_P0002194 | lnc-LCLAT1-2 | 3.492 |
| A_23_P36905 | MLNR | 3.490 |
| A_21_P0012388 | XLOC_l2_009811 | 3.485 |
| A_33_P3371305 | LOC644090 | 3.484 |
| A_21_P0000695 | LOC731424 | 3.480 |
| A_21_P0010330 | LOC100507657 | 3.468 |
| A_32_P133884 | TUSC1 | 3.461 |
| A_23_P428373 | REXO1L1P | 3.453 |
| A_33_P3397336 | ZNF461 | 3.436 |
| A_33_P3397768 | PPP6R2 | 3.434 |
| A_33_P3222429 | MEI4 | 3.410 |
| A_33_P3281990 | OR52E8 | 3.406 |
| A_21_P0002293 | LINC01120 | 3.393 |
| A_21_P0007551 | lnc-C12orf68-1 | 3.393 |
| A_23_P125809 | ZCCHC12 | 3.390 |
| A_21_P0004575 | LOC102546298 | 3.389 |
| A_33_P3424491 | OR51E1 | 3.383 |
| A_23_P70095 | CD74 | 3.374 |
| A_24_P307599 | NAG20 | 3.371 |
| A_33_P3232861 | lnc-TCTN3-1 | 3.368 |
| A_33_P3282454 | SLC12A1 | 3.363 |
| A_33_P3244007 | NEBL | 3.332 |
| A_21_P0011367 | XLOC_l2_004870 | 3.332 |
| A_21_P0009268 | lnc-MAP2K6-2 | 3.318 |
| A_21_P0009800 | lnc-AC008686.1-1 | 3.282 |
| A_33_P3379816 | SMYD1 | 3.278 |
| A_21_P0010975 | XLOC_l2_002311 | 3.273 |
| A_23_P250302 | CCR3 | 3.271 |
| A_21_P0010210 | lnc-WDR4-4 | 3.262 |
| A_21_P0004939 | lnc-LAMA2-1 | 3.260 |
| A_33_P3367301 | GJD3 | 3.249 |
| A_33_P3793707 | LINC00964 | 3.224 |
| A_21_P0008201 | LOC101927248 | 3.212 |
| A_21_P0006993 | lnc-GDF10-3 | 3.209 |
| A_33_P3317225 | OR2L5 | 3.207 |
| A_23_P386310 | HRH4 | 3.199 |
| A_33_P3768992 | DCAF4L2 | 3.197 |
| A_23_P52974 | GIF | 3.190 |
| A_21_P0004441 | lnc-C5orf63-1 | 3.163 |
| A_33_P3677298 | LOC152286 | 3.136 |
| A_33_P3303697 | CR2 | 3.131 |
| A_21_P0009516 | lnc-DLGAP1-1 | 3.105 |
| A_23_P319633 | ZDHHC15 | 3.097 |
| A_23_P18913 | OR2V2 | 3.081 |
| A_21_P0007058 | lnc-C10orf136-1 | 3.070 |
| A_33_P3334679 | SNCAIP | 3.068 |
| A_21_P0000454 | SNORD116-29 | 3.065 |
| A_33_P3317163 | TFAP2B | 3.063 |
| A_24_P199251 | RASSF9 | 3.055 |
| A_33_P3276585 | IFT74 | 3.048 |
| A_33_P3287690 | CNOT2 | 3.031 |
| A_32_P6015 | MNX1 | 3.011 |
| A_21_P0010945 | LOC101929287 | 3.007 |
| A_21_P0013361 | XLOC_l2_014077 | 2.996 |
| A_21_P0012864 | LINC01411 | 2.991 |
| A_23_P346327 | FLJ30679 | 2.987 |
| A_23_P129225 | TRPM1 | 2.986 |
| A_24_P306892 | B4GALNT3 | 2.984 |
| A_21_P0003653 | lnc-PCDH10-8 | 2.983 |
| A_32_P888644 | MGC32805 | 2.980 |
| A_21_P0012494 | XLOC_l2_009790 | 2.977 |
| A_33_P3313710 | LOC102724724 | 2.972 |
| A_33_P3275600 | PLA2G4E | 2.964 |
| A_24_P182947 | GCSAM | 2.948 |
| A_33_P3507562 | SNHG1 | 2.946 |
| A_23_P344194 | CCDC184 | 2.944 |
| A_21_P0013448 | LOC101928036 | 2.942 |
| A_21_P0001055 | lnc-TRAF5-4 | 2.941 |
| A_33_P3408152 | NEFH | 2.927 |
| A_33_P3212232 | MPEG1 | 2.924 |
| A_21_P0009535 | lnc-OSBPL1A-7 | 2.913 |
| A_21_P0009506 | lnc-GALR1-1 | 2.912 |
| A_33_P3394744 | DMRT2 | 2.907 |
| A_33_P3333272 | TMEM235 | 2.907 |
| A_21_P0007389 | lnc-PDGFD-5 | 2.907 |
| A_23_P217946 | CDH23 | 2.906 |
| A_23_P312300 | SCGB2A1 | 2.893 |
| A_21_P0008242 | lnc-GPC5-2 | 2.891 |
| A_21_P0008085 | lnc-SCEL-2 | 2.890 |
| A_21_P0012221 | ANKRD20A11P | 2.889 |
| A_33_P3247589 | LOC102723456 | 2.884 |
| A_24_P376379 | LOC647323 | 2.870 |
| A_33_P3352632 | LOC728147 | 2.865 |
| A_23_P153301 | CEACAM5 | 2.863 |
| A_33_P3210880 | ATP7A | 2.860 |
| A_21_P0002779 | TM4SF1-AS1 | 2.858 |
| A_33_P3392867 | PPEF2 | 2.858 |
| A_23_P132760 | TRH | 2.855 |
| A_32_P194312 | SDK2 | 2.844 |
| A_23_P162386 | BIN2 | 2.841 |
| A_33_P3377187 | LINC00634 | 2.837 |
| A_23_P23296 | PKP1 | 2.836 |
| A_33_P3395916 | XLOC_l2_005020 | 2.831 |
| A_33_P3266823 | VWA5B2 | 2.830 |
| A_21_P0004549 | lnc-SLC12A7-1 | 2.825 |
| A_33_P3249037 | PRRG3 | 2.823 |
| A_23_P34424 | KCNQ4 | 2.817 |
| A_23_P56913 | CIB4 | 2.817 |
| A_33_P3281299 | PCDHA10 | 2.812 |
| A_21_P0000217 | SNORD36B | 2.812 |
| A_23_P389897 | NGFR | 2.806 |
| A_21_P0004858 | lnc-BTN3A2-1 | 2.801 |
| A_23_P129367 | DRC7 | 2.801 |
| A_24_P86246 | PRDM9 | 2.799 |
| A_21_P0001187 | ILDR2 | 2.798 |
| A_21_P0008798 | lnc-RP11-82I10.1.1-4 | 2.794 |
| A_21_P0004475 | lnc-PROP1-3 | 2.787 |
| A_21_P0000048 | DISC1 | 2.785 |
| A_19_P00320727 | LOC101927237 | 2.767 |
| A_21_P0011917 | XLOC_l2_008014 | 2.763 |
| A_21_P0008171 | lnc-DIAPH3-1 | 2.761 |
| A_21_P0003135 | lnc-VGLL3-3 | 2.756 |
| A_23_P387585 | FAM226A | 2.754 |
| A_21_P0000352 | SCARNA4 | 2.748 |
| A_21_P0003848 | LOC101929654 | 2.742 |
| A_21_P0008363 | lnc-FLRT2-2 | 2.738 |
| A_19_P00321571 | LINC01108 | 2.735 |
| A_33_P3343962 | LOC100130373 | 2.735 |
| A_21_P0013963 | LOC100507445 | 2.728 |
| A_33_P3409518 | TUBBP5 | 2.723 |
| A_21_P0000582 | LOC100134317 | 2.708 |
| A_21_P0011031 | LOC101928865 | 2.708 |
| A_21_P0011644 | LINC00483 | 2.705 |
| A_33_P3358005 | LOC102725407 | 2.704 |
| A_33_P3353737 | ADH1B | 2.687 |
| A_21_P0010038 | lnc-MAFB-1 | 2.687 |
| A_24_P354724 | TAGAP | 2.684 |
| A_21_P0008844 | lnc-FSIP1-6 | 2.680 |
| A_23_P171388 | TMSB4Y | 2.679 |
| A_33_P3228977 | UTY | 2.672 |
| A_21_P0011325 | XLOC_l2_004647 | 2.671 |
| A_33_P3370305 | TMEM106A | 2.671 |
| A_23_P71624 | PAX5 | 2.665 |
| A_21_P0008817 | lnc-SNURF-5 | 2.664 |
| A_21_P0007338 | APIP | 2.662 |
| A_21_P0000523 | LINC00486 | 2.660 |
| A_33_P3433388 | TSL | 2.656 |
| A_21_P0000923 | lnc-C1orf31-7 | 2.655 |
| A_33_P3276718 | HGF | 2.654 |
| A_21_P0002578 | lnc-AC010872.2.1-5 | 2.653 |
| A_21_P0011007 | TRIM49B | 2.650 |
| A_21_P0007050 | lnc-GPR158-2 | 2.647 |
| A_21_P0004937 | lnc-TRMT11-1 | 2.636 |
| A_21_P0000143 | DCTN5 | 2.634 |
| A_21_P0010613 | XLOC_l2_000902 | 2.624 |
| A_23_P130241 | KRTAP1-3 | 2.623 |
| A_23_P167585 | GDF9 | 2.620 |
| A_21_P0009987 | lnc-CDH4-2 | 2.620 |
| A_21_P0002437 | lnc-PIGF-3 | 2.620 |
| A_33_P3387493 | FTH1P18 | 2.619 |
| A_23_P51918 | SEC16B | 2.618 |
| A_23_P212655 | KLHL6 | 2.617 |
| A_23_P48561 | EFS | 2.612 |
| A_21_P0001621 | lnc-C1orf227-1 | 2.595 |
| A_21_P0008925 | lnc-CTD-2144E22.5.1-7 | 2.593 |
| A_23_P131789 | BPI | 2.592 |
| A_24_P314534 | KRTAP13-2 | 2.591 |
| A_21_P0011646 | LINC00511 | 2.588 |
| A_21_P0004533 | lnc-MAT2B-3 | 2.583 |
| A_21_P0005412 | lnc-PIK3CG-4 | 2.582 |
| A_33_P3364854 | TRPV6 | 2.579 |
| A_21_P0002990 | lnc-CADM2-3 | 2.577 |
| A_21_P0011756 | LOC101059948 | 2.576 |
| A_21_P0007681 | lnc-PARP11-3 | 2.570 |
| A_33_P3212684 | LOC100996890 | 2.569 |
| A_33_P3273623 | CCL21 | 2.566 |
| A_21_P0005497 | lnc-FIGNL1-1 | 2.564 |
| A_21_P0014152 | lnc-DOPEY1-1 | 2.561 |
| A_33_P3307297 | ARPP21 | 2.556 |
| A_21_P0005526 | lnc-WASL-1 | 2.555 |
| A_21_P0013158 | LOC101928277 | 2.542 |
| A_33_P3344861 | LOC389602 | 2.542 |
| A_23_P56328 | PLVAP | 2.541 |
| A_23_P163025 | RNASE3 | 2.540 |
| A_23_P63096 | VHLL | 2.530 |
| A_21_P0004207 | lnc-C5orf38-3 | 2.529 |
| A_23_P85265 | SRY | 2.528 |
| A_33_P3485976 | LOC283177 | 2.526 |
| A_19_P00323692 | XIST | 2.524 |
| A_21_P0005705 | lnc-CLVS1-3 | 2.518 |
| A_24_P225878 | KIF9 | 2.515 |
| A_21_P0005555 | LOC100506725 | 2.514 |
| A_21_P0010420 | lnc-RP1-32I10.10.1-1 | 2.505 |
| A_33_P3560878 | LINC01146 | 2.503 |
| A_33_P3222391 | FILIP1L | 2.502 |
| A_21_P0000005 | SMTNL1 | 2.502 |
| A_23_P305914 | WSCD2 | 2.498 |
| A_21_P0008012 | LINC00353 | 2.486 |
| A_21_P0006906 | lnc-SORCS3-6 | 2.486 |
| A_21_P0010416 | POLR2F | 2.484 |
| A_32_P129862 | KCNK10 | 2.482 |
| A_33_P3246774 | XLOC_l2_015848 | 2.481 |
| A_21_P0006493 | lnc-KDM6A-2 | 2.466 |
| A_21_P0001763 | LOC100506929 | 2.465 |
| A_33_P3406623 | TNFSF12 | 2.464 |
| A_21_P0014035 | MUC19 | 2.463 |
| A_33_P3279959 | PLXNA4 | 2.461 |
| A_21_P0012867 | LOC728554 | 2.449 |
| A_21_P0008313 | lnc-C14orf23-4 | 2.442 |
| A_33_P3328184 | lnc-CAST-4 | 2.438 |
| A_33_P3253578 | OR8B2 | 2.437 |
| A_33_P3353732 | LOC100131581 | 2.436 |
| A_21_P0006895 | lnc-PCGF5-1 | 2.430 |
| A_21_P0010895 | XLOC_l2_002049 | 2.428 |
| A_24_P391918 | L3MBTL4 | 2.424 |
| A_21_P0001986 | LOC727944 | 2.419 |
| A_21_P0009624 | lnc-AP002414.1.1-1 | 2.417 |
| A_23_P368805 | HHLA2 | 2.417 |
| A_23_P84596 | MZB1 | 2.416 |
| A_23_P143526 | S100B | 2.404 |
| A_24_P294821 | SYNJ2 | 2.400 |
| A_21_P0012218 | XLOC_l2_009136 | 2.399 |
| A_21_P0014304 | lnc-CHSY1-5 | 2.398 |
| A_21_P0009839 | lnc-FAM182A-2 | 2.397 |
| A_21_P0007413 | lnc-BLID-2 | 2.394 |
| A_21_P0009300 | lnc-PMP22-2 | 2.394 |
| A_23_P70102 | SLC36A2 | 2.392 |
| A_24_P380349 | HTR1E | 2.392 |
| A_21_P0002428 | COX7A2L | 2.390 |
| A_21_P0005435 | lnc-AC015987.1-3 | 2.388 |
| A_33_P3420816 | GDF1 | 2.386 |
| A_32_P117693 | FAM19A1 | 2.386 |
| A_33_P3315819 | lnc-C2orf54-2 | 2.386 |
| A_33_P3209326 | lnc-MAB21L2-1 | 2.379 |
| A_21_P0011155 | XLOC_l2_003820 | 2.379 |
| A_21_P0011899 | XLOC_l2_007876 | 2.371 |
| A_33_P3367541 | lnc-SEC61G-7 | 2.370 |
| A_21_P0005615 | LOC101929622 | 2.368 |
| A_32_P87697 | HLA-DRA | 2.367 |
| A_32_P33083 | VCX2 | 2.365 |
| A_21_P0008842 | lnc-POTEB-5 | 2.364 |
| A_21_P0010773 | FLJ43315 | 2.360 |
| A_21_P0002221 | lnc-VRK2-2 | 2.352 |
| A_21_P0012349 | ZDHHC8P1 | 2.346 |
| A_33_P3250555 | LOC102723882 | 2.344 |
| A_33_P3829391 | LOC641510 | 2.343 |
| A_23_P208334 | PDE4A | 2.342 |
| A_21_P0010110 | LOC102606466 | 2.341 |
| A_23_P211417 | RFPL1 | 2.341 |
| A_21_P0009363 | lnc-BTBD17-4 | 2.340 |
| A_32_P438767 | NEUROG2 | 2.339 |
| A_32_P890614 | LOC100268168 | 2.329 |
| A_21_P0001541 | LOC101927952 | 2.327 |
| A_33_P3402284 | LINC00284 | 2.323 |
| A_23_P395418 | PCDHGB3 | 2.323 |
| A_21_P0006557 | lnc-MBTPS2-1 | 2.319 |
| A_21_P0001082 | lnc-SCCPDH-1 | 2.316 |
| A_32_P195719 | CENPVP2 | 2.310 |
| A_24_P297888 | MTAP | 2.304 |
| A_21_P0012164 | FAM182B | 2.303 |
| A_33_P3224338 | LOC101927468 | 2.295 |
| A_23_P348253 | CDHR3 | 2.290 |
| A_24_P348989 | LILRA1 | 2.288 |
| A_21_P0014587 | LINC00845 | 2.288 |
| A_21_P0012038 | LOC101927641 | 2.286 |
| A_33_P3381097 | OR10V1 | 2.286 |
| A_21_P0010068 | lnc-HRH3-1 | 2.285 |
| A_24_P911906 | PCDH17 | 2.284 |
| A_23_P317756 | ACSM3 | 2.282 |
| A_21_P0006749 | LINC01264 | 2.280 |
| A_33_P3589033 | DIAPH2-AS1 | 2.279 |
| A_23_P374294 | CCDC7 | 2.275 |
| A_21_P0005883 | lnc-RP11-778D12.2.1-2 | 2.272 |
| A_21_P0003079 | lnc-PYDC2-1 | 2.270 |
| A_23_P365201 | OTOP3 | 2.268 |
| A_33_P3398251 | FOXP3 | 2.267 |
| A_21_P0006806 | LINC01167 | 2.267 |
| A_23_P93169 | LGSN | 2.266 |
| A_23_P351148 | SH2D1B | 2.266 |
| A_33_P3216568 | MUC5AC | 2.264 |
| A_33_P3350575 | OC90 | 2.261 |
| A_24_P406132 | MAPK13 | 2.259 |
| A_21_P0009077 | lnc-GINS2-3 | 2.258 |
| A_21_P0010696 | LINC00339 | 2.257 |
| A_33_P3268582 | OR4F29 | 2.257 |
| A_21_P0004981 | LOC102723922 | 2.255 |
| A_23_P363331 | GUCA1C | 2.253 |
| A_33_P3287939 | FAM221A | 2.250 |
| A_24_P305223 | CTAGE1 | 2.248 |
| A_23_P168165 | LINC00574 | 2.242 |
| A_21_P0014047 | C4A | 2.242 |
| A_21_P0010197 | lnc-CCT8-1 | 2.241 |
| A_23_P23292 | RXRG | 2.240 |
| A_23_P154050 | CDK15 | 2.239 |
| A_33_P3709525 | LOC100128233 | 2.235 |
| A_21_P0006865 | LINC00840 | 2.235 |
| A_21_P0002405 | lnc-NTSR2-5 | 2.233 |
| A_33_P3332492 | FANK1 | 2.230 |
| A_21_P0014786 | LOC100507663 | 2.227 |
| A_21_P0014515 | LOC101928461 | 2.225 |
| A_33_P3424204 | DLEU2L | 2.224 |
| A_33_P3217427 | DNAJC16 | 2.223 |
| A_23_P9496 | LHX3 | 2.222 |
| A_21_P0011192 | XLOC_l2_004072 | 2.220 |
| A_21_P0013314 | XLOC_l2_013859 | 2.220 |
| A_32_P183442 | XLOC_l2_011265 | 2.218 |
| A_33_P3379251 | LRRC30 | 2.217 |
| A_33_P3291279 | SOWAHB | 2.215 |
| A_21_P0013632 | XLOC_l2_015172 | 2.215 |
| A_21_P0014682 | TMC3-AS1 | 2.211 |
| A_24_P49106 | TCEAL7 | 2.210 |
| A_21_P0010612 | TRABD2B | 2.208 |
| A_23_P316085 | SPG20-AS1 | 2.206 |
| A_21_P0014666 | LOC100287329 | 2.202 |
| A_33_P3318642 | MC1R | 2.201 |
| A_33_P3240972 | GOLGA6L7P | 2.198 |
| A_33_P3353372 | LMBR1 | 2.197 |
| A_21_P0008401 | lnc-RP11-982M15.6.1-2 | 2.196 |
| A_21_P0007304 | lnc-GLB1L2-1 | 2.196 |
| A_33_P3333045 | RABL2A | 2.196 |
| A_19_P00322188 | LOC100507651 | 2.196 |
| A_21_P0007898 | lnc-DHX37-9 | 2.193 |
| A_33_P3396578 | GRK5 | 2.192 |
| A_19_P00316415 | lnc-ERP44-3 | 2.192 |
| A_32_P199901 | RIPPLY1 | 2.189 |
| A_33_P3312985 | LINC00528 | 2.187 |
| A_19_P00805108 | LINC01337 | 2.184 |
| A_23_P48740 | DIO2 | 2.182 |
| A_33_P3255929 | LOC100130502 | 2.178 |
| A_21_P0006757 | lnc-RBP3-1 | 2.177 |
| A_21_P0009312 | lnc-ACCN1-2 | 2.176 |
| A_21_P0008731 | lnc-OIP5-1 | 2.176 |
| A_21_P0014280 | LOC100128276 | 2.175 |
| A_23_P128323 | SCNN1A | 2.175 |
| A_33_P3359724 | C1orf194 | 2.174 |
| A_33_P3284838 | LINC01552 | 2.174 |
| A_21_P0002479 | lnc-TGFBRAP1-11 | 2.173 |
| A_21_P0002671 | LOC101927070 | 2.170 |
| A_33_P3217356 | TRABD2B | 2.168 |
| A_21_P0004890 | LOC102724327 | 2.167 |
| A_21_P0012132 | LOC102724529 | 2.167 |
| A_23_P215331 | CRHR2 | 2.167 |
| A_33_P3339481 | KCNQ5 | 2.166 |
| A_23_P133712 | CYP39A1 | 2.164 |
| A_33_P3677559 | LOC100129461 | 2.163 |
| A_19_P00802679 | CCDC7 | 2.162 |
| A_33_P3276678 | VSX1 | 2.161 |
| A_21_P0006844 | lnc-MYO3A-1 | 2.161 |
| A_21_P0009352 | lnc-ABCA5-1 | 2.160 |
| A_24_P271773 | PCDHB17P | 2.160 |
| A_33_P3329448 | MEIS1-AS3 | 2.159 |
| A_21_P0011229 | LINC00371 | 2.157 |
| A_21_P0008820 | lnc-GOLGA8J-1 | 2.154 |
| A_21_P0009124 | LOC100996345 | 2.153 |
| A_21_P0005410 | lnc-ZKSCAN1-1 | 2.152 |
| A_21_P0012549 | RPL32P3 | 2.150 |
| A_23_P170378 | PMCHL1 | 2.149 |
| A_23_P124619 | S100A14 | 2.149 |
| A_33_P3746734 | LINC00251 | 2.148 |
| A_33_P3299309 | RPL28 | 2.148 |
| A_21_P0008095 | lnc-NDFIP2-10 | 2.146 |
| A_23_P316582 | ZNF441 | 2.143 |
| A_23_P21316 | PRUNE | 2.142 |
| A_21_P0004208 | lnc-C5orf38-4 | 2.141 |
| A_21_P0001727 | lnc-SLC6A9-1 | 2.140 |
| A_24_P408704 | DOCK2 | 2.138 |
| A_23_P325155 | CD200R1 | 2.136 |
| A_21_P0010772 | LOC102725076 | 2.135 |
| A_33_P3259708 | CMA1 | 2.132 |
| A_21_P0008358 | lnc-C14orf118-1 | 2.131 |
| A_33_P3291445 | PIGK | 2.130 |
| A_21_P0003968 | LOC101929284 | 2.130 |
| A_33_P3418662 | HSD17B3 | 2.126 |
| A_33_P3421530 | HMCN2 | 2.125 |
| A_33_P3241244 | LOC100131822 | 2.124 |
| A_21_P0009869 | lnc-SLCO4A1-1 | 2.123 |
| A_33_P3373343 | ARSH | 2.123 |
| A_21_P0000281 | SNORD59A | 2.121 |
| A_23_P364414 | PCDHGA9 | 2.120 |
| A_33_P3348224 | SEC14L6 | 2.116 |
| A_23_P18362 | SLITRK3 | 2.115 |
| A_19_P00319578 | LINC01094 | 2.115 |
| A_33_P3865962 | C1orf143 | 2.112 |
| A_24_P6517 | PLEKHG1 | 2.111 |
| A_21_P0010692 | MST1P2 | 2.110 |
| A_33_P3379268 | SCIMP | 2.109 |
| A_21_P0006287 | lnc-CDK20-1 | 2.108 |
| A_33_P3361112 | ABCG8 | 2.108 |
| A_21_P0003283 | lnc-ANO10-1 | 2.108 |
| A_21_P0007440 | LOC102724209 | 2.106 |
| A_32_P202182 | SPDYE8P | 2.106 |
| A_23_P67932 | CXCR1 | 2.105 |
| A_21_P0005136 | lnc-TNFAIP3-1 | 2.105 |
| A_23_P92334 | TMEM156 | 2.102 |
| A_21_P0000823 | PAXBP1-AS1 | 2.099 |
| A_21_P0014665 | LOC102723630 | 2.099 |
| A_21_P0007787 | LINC00944 | 2.097 |
| A_33_P3304157 | COL19A1 | 2.096 |
| A_33_P3239620 | SPINT3 | 2.096 |
| A_33_P3265024 | ST5 | 2.094 |
| A_23_P98830 | UBQLN3 | 2.092 |
| A_21_P0003250 | DNAJB8-AS1 | 2.092 |
| A_24_P742352 | LINC00442 | 2.092 |
| A_21_P0001511 | lnc-C1orf168-2 | 2.091 |
| A_21_P0009261 | lnc-MSI2-2 | 2.088 |
| A_21_P0014328 | LOC102723466 | 2.088 |
| A_21_P0014177 | LOC101926963 | 2.086 |
| A_21_P0014298 | lnc-PRC1-1 | 2.086 |
| A_21_P0001966 | lnc-ATIC-6 | 2.086 |
| A_23_P369090 | DUSP15 | 2.084 |
| A_21_P0002009 | LINC00298 | 2.082 |
| A_33_P3400244 | ZNF496 | 2.082 |
| A_23_P38795 | FPR1 | 2.079 |
| A_21_P0007693 | lnc-IFLTD1-1 | 2.078 |
| A_21_P0004018 | lnc-AGGF1-1 | 2.078 |
| A_33_P3401058 | lnc-KIAA0825-1 | 2.077 |
| A_32_P129214 | LINC00320 | 2.076 |
| A_21_P0008847 | LOC102724553 | 2.076 |
| A_33_P3213665 | LOC728084 | 2.075 |
| A_23_P125435 | GABRB1 | 2.074 |
| A_33_P3875570 | LOC100628314 | 2.074 |
| A_21_P0001160 | lnc-DBT-1 | 2.073 |
| A_23_P168761 | PTPRZ1 | 2.073 |
| A_33_P3327046 | lnc-MRPL14-1 | 2.072 |
| A_21_P0012423 | XLOC_l2_010056 | 2.071 |
| A_33_P3252598 | DEFB136 | 2.071 |
| A_21_P0008056 | lnc-C13orf15-1 | 2.070 |
| A_33_P3395562 | C1orf141 | 2.069 |
| A_23_P152082 | SPTBN5 | 2.069 |
| A_21_P0012145 | lnc-SPTLC3-2 | 2.068 |
| A_21_P0003703 | lnc-TRIML1-5 | 2.066 |
| A_23_P4254 | CRYBA1 | 2.065 |
| A_21_P0003691 | lnc-RP11-701P16.2.1-2 | 2.065 |
| A_21_P0014399 | PLCG1-AS1 | 2.064 |
| A_21_P0004041 | LINC00492 | 2.063 |
| A_19_P00809747 | LOC100507537 | 2.063 |
| A_33_P3552465 | ZNF852 | 2.063 |
| A_21_P0010275 | lnc-TIAM1-1 | 2.062 |
| A_24_P209047 | IL5 | 2.061 |
| A_33_P3306843 | ZNF836 | 2.060 |
| A_24_P323997 | TPRX1 | 2.059 |
| A_21_P0013525 | lnc-C8orf83-2 | 2.059 |
| A_33_P3381398 | VAX1 | 2.057 |
| A_33_P3272568 | PRDX4 | 2.057 |
| A_23_P212508 | TF | 2.056 |
| A_21_P0002222 | lnc-VRK2-1 | 2.056 |
| A_21_P0014737 | ZFPM2 | 2.055 |
| A_21_P0007196 | lnc-API5-5 | 2.054 |
| A_21_P0005755 | lnc-UTP23-1 | 2.053 |
| A_19_P00316164 | LOC101928858 | 2.052 |
| A_19_P00326248 | lnc-CHIC1-2 | 2.052 |
| A_24_P111147 | PCDH15 | 2.050 |
| A_24_P41706 | OFCC1 | 2.049 |
| A_21_P0014887 | LOC100652768 | 2.048 |
| A_24_P186114 | TTTY21 | 2.045 |
| A_21_P0007331 | lnc-MPPED2-2 | 2.043 |
| A_21_P0004460 | lnc-EBF1-2 | 2.042 |
| A_33_P3214204 | TRIQK | 2.039 |
| A_33_P3413558 | CD226 | 2.039 |
| A_24_P101704 | ROR2 | 2.039 |
| A_32_P42537 | LOC100132304 | 2.038 |
| A_23_P425990 | MTUS2 | 2.038 |
| A_33_P3266564 | KRTAP5-6 | 2.037 |
| A_23_P301328 | PDXK | 2.037 |
| A_33_P3416022 | FAM99A | 2.034 |
| A_21_P0008930 | lnc-RP11-22D3.1.1-3 | 2.034 |
| A_21_P0003025 | lnc-CCDC37-4 | 2.033 |
| A_33_P3316052 | CA5B | 2.033 |
| A_33_P3350343 | LOC101929115 | 2.032 |
| A_21_P0013036 | XLOC_l2_012724 | 2.031 |
| A_33_P3352887 | LOC388692 | 2.031 |
| A_21_P0002414 | LOC102723389 | 2.031 |
| A_23_P145935 | EPHB6 | 2.031 |
| A_24_P356 | AAK1 | 2.030 |
| A_24_P237936 | TCF23 | 2.028 |
| A_33_P3419190 | AREG | 2.026 |
| A_23_P384329 | DENND4A | 2.026 |
| A_21_P0011166 | XLOC_l2_003877 | 2.025 |
| A_33_P3320563 | GLOD5 | 2.023 |
| A_21_P0009099 | CASC22 | 2.023 |
| A_24_P194337 | PRDM15 | 2.023 |
| A_23_P218937 | PCDHGC3 | 2.022 |
| A_23_P251881 | NCR3 | 2.022 |
| A_23_P23873 | PAPPA2 | 2.021 |
| A_21_P0008189 | lnc-SLITRK1-2 | 2.020 |
| A_21_P0005119 | lnc-SH3BGRL2-1 | 2.020 |
| A_19_P00318310 | lnc-TRIM39-1 | 2.017 |
| A_33_P3355508 | FOXL2 | 2.017 |
| A_24_P85537 | MAP3K13 | 2.016 |
| A_21_P0009085 | FENDRR | 2.016 |
| A_33_P3288659 | ACTL8 | 2.015 |
| A_33_P3330608 | PRAM1 | 2.015 |
| A_21_P0000856 | IL10RB-AS1 | 2.014 |
| A_21_P0009532 | lnc-RP11-863N1.2.1-5 | 2.013 |
| A_23_P364437 | CDH23 | 2.013 |
| A_33_P3395249 | LINC00705 | 2.012 |
| A_32_P741851 | GLB1L3 | 2.011 |
| A_21_P0011169 | LINC00417 | 2.010 |
| A_23_P16275 | TSKS | 2.010 |
| A_33_P3595369 | RGR | 2.010 |
| A_21_P0012144 | LINC01271 | 2.009 |
| A_21_P0009960 | lnc-NCOA3-2 | 2.008 |
| A_21_P0000396 | SNORD112 | 2.008 |
| A_21_P0005946 | lnc-FAM83A-1 | 2.008 |
| A_21_P0008042 | lnc-USPL1-2 | 2.008 |
| A_24_P309216 | TPD52L3 | 2.007 |
| A_21_P0006987 | lnc-CUL2-1 | 2.007 |
| A_33_P3389649 | PDE4D | 2.006 |
| A_21_P0006040 | lnc-ACTL7A-1 | 2.005 |
| A_23_P396115 | SSX7 | 2.003 |
| A_21_P0009260 | lnc-PCTP-1 | 2.003 |
| A_33_P3821313 | LINC01019 | 2.001 |
| A_33_P3401980 | SPRED3 | 2.000 |

*Fold change shows the expression level for H2052 treated by anti-S100A11 antibody relative to parental H2052.

(B) Down-regulated genes in H2052 treated by anti-S100A11 antibody compared to parental H2052.

| **ProbeName** | **GeneSymbol** | **Fold change** |
| --- | --- | --- |
| A_21_P0004558 | lnc-SGTB-4 | 0.18835 |
| A_21_P0004889 | lnc-OPN5-1 | 0.19414 |
| A_21_P0005082 | LOC102724152 | 0.19682 |
| A_21_P0002535 | lnc-KLF7-1 | 0.20192 |
| A_33_P3338634 | SYT14 | 0.20197 |
| A_32_P134968 | SPTB | 0.20288 |
| A_21_P0003282 | lnc-NGLY1-1 | 0.21005 |
| A_32_P164593 | ZMAT4 | 0.21102 |
| A_21_P0009349 | lnc-C17orf58-5 | 0.21168 |
| A_33_P3384695 | NCALD | 0.21317 |
| A_21_P0014044 | lnc-BMP7-1 | 0.21436 |
| A_21_P0012435 | LINC01267 | 0.21634 |
| A_23_P159952 | BEX1 | 0.21684 |
| A_24_P595237 | SYNDIG1L | 0.21714 |
| A_23_P168357 | CPA1 | 0.21719 |
| A_33_P3285779 | LOC340113 | 0.21839 |
| A_21_P0005881 | lnc-SLC25A32-3 | 0.21900 |
| A_23_P374281 | CDC20B | 0.21951 |
| A_33_P3274416 | LOC653712 | 0.21965 |
| A_21_P0005168 | lnc-SUPT3H-1 | 0.22098 |
| A_33_P3392740 | ATOH8 | 0.22430 |
| A_33_P3340782 | SPINK5 | 0.22539 |
| A_21_P0012818 | LOC100506272 | 0.22681 |
| A_23_P323196 | MDS2 | 0.22722 |
| A_33_P3282018 | MCF2L | 0.23013 |
| A_21_P0007691 | lnc-SLCO1A2-3 | 0.23194 |
| A_21_P0009244 | lnc-LRRC3C-1 | 0.23374 |
| A_24_P144601 | POU5F1 | 0.23400 |
| A_21_P0001167 | LOC100996263 | 0.23452 |
| A_33_P3240717 | CDKN2B-AS1 | 0.23653 |
| A_21_P0004936 | lnc-CLVS2-2 | 0.23697 |
| A_21_P0002329 | lnc-ZNF804A-1 | 0.23707 |
| A_23_P404575 | RBM46 | 0.23974 |
| A_23_P165201 | PRODH2 | 0.23991 |
| A_33_P3320077 | NFIB | 0.24050 |
| A_33_P3352382 | ARG1 | 0.24086 |
| A_21_P0002190 | lnc-BRE-1 | 0.24138 |
| A_21_P0004673 | OSTM1-AS1 | 0.24262 |
| A_21_P0010165 | lnc-PKNOX1-1 | 0.24340 |
| A_23_P24774 | ABCC8 | 0.24427 |
| A_21_P0005438 | lnc-RP11-1220K2.2.1-1 | 0.24445 |
| A_21_P0010411 | lnc-ISX-2 | 0.24455 |
| A_23_P212675 | NME9 | 0.24482 |
| A_21_P0000815 | LOC100506122 | 0.24638 |
| A_24_P159434 | CD300A | 0.24748 |
| A_33_P3539684 | LOC153910 | 0.24775 |
| A_21_P0011239 | TTC6 | 0.24810 |
| A_21_P0007052 | LINC00202-2 | 0.24920 |
| A_21_P0000046 | ZNF135 | 0.25008 |
| A_21_P0000634 | LOC100129046 | 0.25107 |
| A_21_P0007482 | FZD10-AS1 | 0.25233 |
| A_21_P0005585 | lnc-INSIG1-1 | 0.25242 |
| A_23_P44421 | HTRA4 | 0.25281 |
| A_21_P0002152 | lnc-ACP1-3 | 0.25378 |
| A_24_P107317 | USP2 | 0.25380 |
| A_33_P3873533 | CYP51A1-AS1 | 0.25380 |
| A_33_P3328327 | lnc-C8orf59-1 | 0.25497 |
| A_33_P3327687 | LOC727993 | 0.25548 |
| A_33_P3397545 | DEFB135 | 0.25624 |
| A_21_P0002183 | lnc-AC010872.2.1-2 | 0.25965 |
| A_21_P0010206 | LOC101928435 | 0.25998 |
| A_21_P0006262 | DNAJB5-AS1 | 0.26045 |
| A_21_P0011511 | XLOC_l2_005644 | 0.26112 |
| A_23_P50250 | CKM | 0.26123 |
| A_23_P406591 | GUSBP5 | 0.26140 |
| A_21_P0003260 | lnc-SEC62-1 | 0.26280 |
| A_23_P67381 | SULT2A1 | 0.26316 |
| A_33_P3256810 | TM4SF4 | 0.26317 |
| A_24_P921897 | HOOK1 | 0.26357 |
| A_21_P0006468 | lnc-IDS-5 | 0.26428 |
| A_32_P149546 | CROCC | 0.26491 |
| A_33_P3266192 | GAS6-AS1 | 0.26510 |
| A_24_P231829 | BHMT2 | 0.26540 |
| A_21_P0002994 | LINC00506 | 0.26542 |
| A_21_P0011218 | ANKRD20A19P | 0.26627 |
| A_21_P0005701 | lnc-SDCBP-1 | 0.26667 |
| A_21_P0010249 | lnc-MX1-1 | 0.26779 |
| A_33_P3311820 | FLJ44674 | 0.26793 |
| A_19_P00321067 | MIAT | 0.26830 |
| A_21_P0006445 | lnc-ACOT9-1 | 0.26986 |
| A_33_P3397530 | OR8I2 | 0.27000 |
| A_19_P00801416 | lnc-BBS12-1 | 0.27048 |
| A_32_P160045 | TCTEX1D1 | 0.27089 |
| A_23_P11644 | SPRR2D | 0.27242 |
| A_23_P115200 | FCRL4 | 0.27269 |
| A_33_P3853081 | ALDOAP2 | 0.27337 |
| A_21_P0002137 | LOC102724675 | 0.27358 |
| A_21_P0005127 | lnc-LACE1-2 | 0.27400 |
| A_32_P86578 | DPP10-AS1 | 0.27448 |
| A_33_P3298850 | SLC16A8 | 0.27548 |
| A_33_P3354703 | MYADML | 0.27696 |
| A_32_P47870 | lnc-PDZD8-1 | 0.27700 |
| A_33_P3384148 | C12orf40 | 0.27765 |
| A_21_P0005708 | LOC101929628 | 0.27777 |
| A_21_P0008671 | lnc-FANCI-3 | 0.27986 |
| A_21_P0012501 | LINC01210 | 0.28040 |
| A_21_P0008774 | lnc-DET1-2 | 0.28066 |
| A_21_P0011862 | XLOC_l2_007586 | 0.28072 |
| A_33_P3303900 | OR52B4 | 0.28082 |
| A_33_P3231727 | LOC100132249 | 0.28089 |
| A_21_P0007793 | lnc-SLC15A4-12 | 0.28120 |
| A_21_P0003739 | lnc-AC021860.1-4 | 0.28154 |
| A_21_P0007679 | lnc-PARP11-1 | 0.28267 |
| A_21_P0004494 | lnc-CDH6-2 | 0.28268 |
| A_21_P0005043 | lnc-GPR63-4 | 0.28284 |
| A_24_P289260 | DACT2 | 0.28318 |
| A_21_P0012733 | lnc-MMRN1-2 | 0.28349 |
| A_32_P453321 | C1orf228 | 0.28364 |
| A_21_P0011353 | XLOC_l2_004844 | 0.28383 |
| A_23_P430842 | HAPLN4 | 0.28476 |
| A_24_P930111 | SLC4A10 | 0.28594 |
| A_23_P80718 | SYNPR | 0.28595 |
| A_21_P0010039 | LOC101927098 | 0.28615 |
| A_21_P0008993 | lnc-JPH3-1 | 0.28617 |
| A_23_P153155 | GALR1 | 0.28618 |
| A_33_P3415247 | LOC115110 | 0.28652 |
| A_21_P0005499 | lnc-ZNF680-11 | 0.28749 |
| A_23_P56604 | IL1RL2 | 0.28760 |
| A_21_P0006880 | lnc-SGPL1-3 | 0.28812 |
| A_21_P0003238 | lnc-CADM2-3 | 0.28895 |
| A_33_P3419562 | OR5K2 | 0.28980 |
| A_21_P0009317 | YWHAEP7 | 0.28981 |
| A_23_P81926 | PSORS1C2 | 0.29101 |
| A_21_P0003852 | lnc-MGST2-1 | 0.29114 |
| A_21_P0002783 | LOC102724289 | 0.29158 |
| A_21_P0008962 | lnc-CDH3-3 | 0.29231 |
| A_23_P17481 | SIGLEC1 | 0.29375 |
| A_23_P431923 | SSX8 | 0.29393 |
| A_21_P0003419 | LOC100996286 | 0.29450 |
| A_33_P3320832 | NMS | 0.29564 |
| A_32_P901770 | ESPN | 0.29678 |
| A_21_P0012643 | XLOC_l2_010976 | 0.29679 |
| A_21_P0006405 | lnc-CD40LG-1 | 0.29840 |
| A_23_P148255 | MAGEA2B | 0.29847 |
| A_21_P0003890 | lnc-HS3ST1-1 | 0.29849 |
| A_33_P3215305 | ANKRD62 | 0.29861 |
| A_33_P3383071 | LOC100128437 | 0.29868 |
| A_24_P281439 | OR2T5 | 0.29899 |
| A_21_P0007904 | lnc-SLC15A4-2 | 0.29996 |
| A_21_P0007339 | lnc-APIP-1 | 0.30104 |
| A_21_P0010837 | XLOC_l2_001667 | 0.30209 |
| A_21_P0011304 | XLOC_l2_004596 | 0.30214 |
| A_21_P0005717 | LOC102724772 | 0.30249 |
| A_23_P334246 | APLF | 0.30249 |
| A_21_P0012538 | lnc-CX3CR1-1 | 0.30258 |
| A_33_P3301485 | GGA1 | 0.30306 |
| A_21_P0012272 | XLOC_l2_009439 | 0.30429 |
| A_33_P3223497 | FRY | 0.30430 |
| A_23_P138352 | WNT2B | 0.30501 |
| A_33_P3322484 | DDI1 | 0.30591 |
| A_21_P0004417 | RASGRF2-AS1 | 0.30635 |
| A_21_P0013799 | XLOC_l2_015641 | 0.30638 |
| A_33_P3405023 | BLACE | 0.30694 |
| A_21_P0013310 | XLOC_l2_013849 | 0.30789 |
| A_33_P3376493 | AGTR2 | 0.30793 |
| A_21_P0012441 | TRANK1 | 0.30798 |
| A_33_P3707547 | LOC541467 | 0.30842 |
| A_19_P00316602 | LOC441204 | 0.30845 |
| A_21_P0007313 | lnc-CDKN1C-1 | 0.30867 |
| A_23_P139786 | OASL | 0.30882 |
| A_33_P3248008 | LOC100652807 | 0.30887 |
| A_21_P0011995 | LOC100506274 | 0.30900 |
| A_23_P52430 | WNT8B | 0.30903 |
| A_21_P0002424 | lnc-DHX57-1 | 0.30977 |
| A_21_P0002642 | LINC01248 | 0.30978 |
| A_21_P0006150 | lnc-C9orf135-1 | 0.31009 |
| A_23_P127352 | MRGPRD | 0.31061 |
| A_33_P3289995 | USP45 | 0.31076 |
| A_21_P0000943 | lnc-AJAP1-2 | 0.31093 |
| A_23_P170541 | KCNH6 | 0.31131 |
| A_33_P3282683 | LOC100130027 | 0.31249 |
| A_21_P0005941 | NCRNA00249 | 0.31326 |
| A_21_P0005782 | lnc-ERICH1-2 | 0.31334 |
| A_21_P0010583 | XLOC_l2_000691 | 0.31362 |
| A_24_P489164 | LOC100131943 | 0.31392 |
| A_33_P3390924 | lnc-MRPL32-1 | 0.31510 |
| A_24_P331675 | C6orf10 | 0.31538 |
| A_21_P0004331 | lnc-NEURL1B-4 | 0.31634 |
| A_32_P123143 | HCN1 | 0.31673 |
| A_32_P2452 | TMTC1 | 0.31691 |
| A_24_P65851 | PPP4R1L | 0.31759 |
| A_24_P63537 | ERAP1 | 0.31765 |
| A_21_P0002996 | lnc-C3orf38-2 | 0.31829 |
| A_21_P0012553 | LINC01192 | 0.31966 |
| A_24_P199484 | ANGPTL1 | 0.32006 |
| A_21_P0010306 | MIR99AHG | 0.32078 |
| A_21_P0008418 | lnc-C14orf126-5 | 0.32086 |
| A_23_P75179 | CFAP46 | 0.32219 |
| A_21_P0008522 | lnc-RP11-1070N10.3.1-1 | 0.32221 |
| A_23_P372994 | H1FOO | 0.32278 |
| A_21_P0013081 | XLOC_l2_013000 | 0.32350 |
| A_33_P3310784 | TM6SF1 | 0.32379 |
| A_19_P00322837 | LINC01470 | 0.32504 |
| A_21_P0006972 | lnc-C10orf31-6 | 0.32596 |
| A_33_P3245952 | LOC648570 | 0.32649 |
| A_21_P0011237 | LINC01087 | 0.32664 |
| A_23_P96633 | CT55 | 0.32670 |
| A_21_P0003637 | lnc-ALPK1-1 | 0.32707 |
| A_21_P0014160 | LOC102725454 | 0.32738 |
| A_33_P3277298 | SYT6 | 0.32820 |
| A_21_P0004045 | LOC102467214 | 0.32823 |
| A_21_P0009564 | lnc-MC4R-1 | 0.32840 |
| A_33_P3305885 | WNK2 | 0.32849 |
| A_21_P0002938 | lnc-UBE2E2-1 | 0.32900 |
| A_21_P0003076 | lnc-DNAJB11-1 | 0.32904 |
| A_33_P3241056 | LOC154872 | 0.32911 |
| A_23_P6293 | UBASH3A | 0.32955 |
| A_33_P3417422 | lnc-VIM-1 | 0.32973 |
| A_24_P68088 | TCAM1P | 0.32998 |
| A_21_P0002016 | LOC100506474 | 0.33045 |
| A_21_P0004582 | lnc-BOD1-1 | 0.33047 |
| A_21_P0012106 | LINC00607 | 0.33056 |
| A_23_P52668 | NAALADL1 | 0.33056 |
| A_23_P43337 | FREM1 | 0.33132 |
| A_33_P3419567 | RAD21L1 | 0.33141 |
| A_21_P0010002 | lnc-HAO1-2 | 0.33230 |
| A_33_P3360814 | NFE4 | 0.33241 |
| A_33_P3365368 | LOC100130278 | 0.33285 |
| A_21_P0006529 | lnc-RPS6KA3-1 | 0.33413 |
| A_33_P3485760 | SNORA19 | 0.33417 |
| A_21_P0002617 | lnc-SLC4A10-4 | 0.33505 |
| A_33_P3355937 | LINC00908 | 0.33535 |
| A_33_P3296772 | FRMD6-AS1 | 0.33582 |
| A_32_P705474 | LOC100507530 | 0.33608 |
| A_21_P0004282 | lnc-MAN2A1-3 | 0.33657 |
| A_21_P0005513 | lnc-SGCE-1 | 0.33659 |
| A_21_P0005300 | lnc-PSMA2-1 | 0.33694 |
| A_21_P0004223 | lnc-ADCY2-1 | 0.33803 |
| A_23_P319232 | KIAA1045 | 0.33925 |
| A_21_P0008714 | lnc-GABRB3-2 | 0.33949 |
| A_21_P0002580 | DNAJC27-AS1 | 0.33983 |
| A_19_P00318363 | lnc-QPCT-2 | 0.34044 |
| A_21_P0007923 | LINC01046 | 0.34047 |
| A_32_P787109 | DTHD1 | 0.34085 |
| A_33_P3247489 | C11orf85 | 0.34090 |
| A_21_P0008945 | lnc-CHD9-4 | 0.34168 |
| A_33_P3243486 | IGFL4 | 0.34170 |
| A_21_P0005940 | LOC101926892 | 0.34175 |
| A_33_P3218178 | SLC25A53 | 0.34221 |
| A_21_P0006258 | lnc-ELAVL2-3 | 0.34229 |
| A_21_P0002133 | LOC101927619 | 0.34255 |
| A_21_P0006225 | lnc-GAPVD1-2 | 0.34302 |
| A_23_P80954 | SLC26A1 | 0.34359 |
| A_33_P3311974 | PROX2 | 0.34437 |
| A_21_P0011168 | CYP4F35P | 0.34465 |
| A_21_P0005063 | lnc-TXLNB-2 | 0.34590 |
| A_21_P0011687 | XLOC_l2_006670 | 0.34630 |
| A_23_P378450 | MBD3L2 | 0.34691 |
| A_21_P0008511 | lnc-CTAGE5-1 | 0.34709 |
| A_21_P0004385 | LOC642366 | 0.34750 |
| A_21_P0010427 | lnc-FAM19A5-1 | 0.34751 |
| A_33_P3282522 | GCNT7 | 0.34778 |
| A_33_P3250865 | CCDC175 | 0.34830 |
| A_21_P0007770 | VSIG10 | 0.34905 |
| A_33_P3372600 | LOC102723640 | 0.34946 |
| A_33_P3280385 | COL6A3 | 0.34983 |
| A_21_P0010240 | LOC102724630 | 0.34987 |
| A_23_P66854 | KRT20 | 0.35010 |
| A_33_P3372635 | CSN1S2AP | 0.35082 |
| A_21_P0000644 | LOC400655 | 0.35113 |
| A_21_P0001265 | lnc-C1orf196-1 | 0.35124 |
| A_24_P333635 | C10orf62 | 0.35231 |
| A_33_P3462975 | LINC01242 | 0.35393 |
| A_33_P3423450 | LOC100130548 | 0.35457 |
| A_23_P167121 | GABRA2 | 0.35502 |
| A_21_P0010277 | lnc-RUNX1-1 | 0.35508 |
| A_33_P3260667 | OR2T34 | 0.35509 |
| A_21_P0010518 | MST1P2 | 0.35513 |
| A_21_P0012168 | LOC102724188 | 0.35548 |
| A_21_P0003021 | LOC102723597 | 0.35548 |
| A_21_P0005710 | lnc-NKAIN3-6 | 0.35628 |
| A_21_P0014286 | LINC00456 | 0.35685 |
| A_21_P0004225 | lnc-MTRR-4 | 0.35705 |
| A_33_P3259712 | lnc-A2M-1 | 0.35708 |
| A_33_P3423506 | NXPE2 | 0.35871 |
| A_23_P420209 | GCNT3 | 0.35900 |
| A_21_P0008311 | lnc-C14orf23-2 | 0.35912 |
| A_21_P0009546 | lnc-CELF4-2 | 0.35948 |
| A_21_P0011420 | XLOC_l2_005150 | 0.35963 |
| A_21_P0003309 | THPO | 0.36025 |
| A_33_P3325665 | JPH4 | 0.36088 |
| A_24_P52293 | OR2A25 | 0.36092 |
| A_21_P0010090 | lnc-NCOA3-7 | 0.36120 |
| A_21_P0005908 | LINC00824 | 0.36146 |
| A_21_P0009822 | lnc-NOP56-1 | 0.36180 |
| A_21_P0001417 | lnc-ATF3-1 | 0.36297 |
| A_23_P350678 | CFAP43 | 0.36300 |
| A_23_P319005 | HMP19 | 0.36353 |
| A_33_P3308045 | EIF4E2 | 0.36450 |
| A_21_P0003007 | LOC102723430 | 0.36524 |
| A_21_P0007366 | lnc-ORAOV1-4 | 0.36553 |
| A_21_P0013031 | XLOC_l2_012661 | 0.36569 |
| A_21_P0008725 | LOC102724253 | 0.36622 |
| A_21_P0004253 | LINCR-0003 | 0.36718 |
| A_33_P3287399 | LOC100190940 | 0.36720 |
| A_24_P406939 | C11orf1 | 0.36738 |
| A_21_P0004387 | LOC102467080 | 0.36787 |
| A_33_P3326730 | CCDC183 | 0.36982 |
| A_24_P369656 | TRIM9 | 0.37081 |
| A_33_P3342300 | VSX1 | 0.37121 |
| A_33_P3395859 | C19orf84 | 0.37180 |
| A_21_P0008825 | lnc-MAPK6-10 | 0.37398 |
| A_32_P200608 | LINC00608 | 0.37433 |
| A_21_P0006761 | lnc-MBL2-5 | 0.37477 |
| A_33_P3396008 | AGER | 0.37488 |
| A_21_P0005217 | lnc-IL6-3 | 0.37534 |
| A_33_P3234949 | GRID1 | 0.37655 |
| A_33_P3238978 | TGIF2LY | 0.37666 |
| A_23_P356425 | AXDND1 | 0.37680 |
| A_23_P81683 | GPRC6A | 0.37813 |
| A_23_P64721 | HCAR3 | 0.37820 |
| A_21_P0003474 | lnc-TECRL-2 | 0.37834 |
| A_21_P0010564 | XLOC_l2_000471 | 0.37846 |
| A_33_P3416331 | SLC10A5 | 0.38044 |
| A_21_P0008384 | lnc-C14orf177-4 | 0.38049 |
| A_21_P0000486 | SNORD103A | 0.38134 |
| A_33_P3677050 | LINC01185 | 0.38201 |
| A_21_P0010193 | lnc-MRPL39-4 | 0.38251 |
| A_21_P0014556 | lnc-ABHD12B-5 | 0.38265 |
| A_23_P150648 | KCNQ1DN | 0.38276 |
| A_21_P0009907 | CSE1L-AS1 | 0.38328 |
| A_21_P0003228 | lnc-GNAI2-1 | 0.38346 |
| A_23_P29735 | BSN | 0.38367 |
| A_21_P0006941 | LOC102723337 | 0.38369 |
| A_24_P68415 | ZNF45 | 0.38404 |
| A_33_P3277898 | VSTM2A | 0.38436 |
| A_23_P374559 | ZNRF4 | 0.38536 |
| A_21_P0000771 | LOC100505715 | 0.38568 |
| A_21_P0005416 | lnc-C7orf53-1 | 0.38572 |
| A_21_P0009292 | lnc-AIPL1-1 | 0.38590 |
| A_23_P59825 | TAS2R16 | 0.38729 |
| A_33_P3268686 | FBXW12 | 0.38752 |
| A_19_P00804359 | LINC01488 | 0.38764 |
| A_21_P0005035 | lnc-HTR1B-1 | 0.38819 |
| A_21_P0008023 | lnc-RASA3-1 | 0.38923 |
| A_33_P3326713 | FAM188B | 0.38952 |
| A_23_P162607 | STAB2 | 0.38960 |
| A_21_P0014207 | LOC101927721 | 0.38991 |
| A_21_P0005769 | lnc-KHDRBS3-9 | 0.39108 |
| A_23_P82567 | PRSS58 | 0.39134 |
| A_21_P0000842 | DDX11-AS1 | 0.39173 |
| A_21_P0002006 | NRIR | 0.39187 |
| A_21_P0002788 | LINC01487 | 0.39231 |
| A_21_P0007175 | lnc-CTR9-4 | 0.39241 |
| A_21_P0009588 | lnc-ZNF516-5 | 0.39268 |
| A_21_P0012761 | LOC102723920 | 0.39293 |
| A_21_P0007806 | FZD10-AS1 | 0.39310 |
| A_33_P3226630 | LINC01140 | 0.39320 |
| A_23_P502336 | EMR2 | 0.39374 |
| A_21_P0006801 | LOC102723646 | 0.39531 |
| A_21_P0014353 | LOC100506882 | 0.39543 |
| A_33_P3251342 | CYP3A4 | 0.39568 |
| A_23_P49657 | MYH1 | 0.39713 |
| A_21_P0009010 | LOC101927311 | 0.39740 |
| A_21_P0004641 | MUC22 | 0.39745 |
| A_19_P00317127 | LOC101929683 | 0.39791 |
| A_21_P0009925 | lnc-SIRPA-1 | 0.39803 |
| A_21_P0009231 | lnc-WSB1-2 | 0.39813 |
| A_19_P00321839 | lnc-HSCB-2 | 0.39818 |
| A_21_P0010392 | lnc-AC007663.1-5 | 0.39869 |
| A_21_P0011489 | XLOC_l2_005553 | 0.40093 |
| A_24_P341674 | DIO2 | 0.40192 |
| A_33_P3312534 | TRAPPC10 | 0.40250 |
| A_23_P13479 | OR4S1 | 0.40358 |
| A_21_P0002537 | lnc-IKZF2-2 | 0.40385 |
| A_21_P0011932 | XLOC_l2_008133 | 0.40399 |
| A_23_P256084 | ARSE | 0.40432 |
| A_23_P108157 | TJP3 | 0.40521 |
| A_23_P39454 | ZNF556 | 0.40561 |
| A_23_P171397 | RBMY1B | 0.40564 |
| A_24_P93720 | LOC645984 | 0.40568 |
| A_24_P309521 | KCNJ5 | 0.40601 |
| A_33_P3722782 | TMEM72-AS1 | 0.40651 |
| A_23_P75063 | DYDC2 | 0.40660 |
| A_23_P8702 | PIP | 0.40668 |
| A_21_P0013337 | XLOC_l2_013931 | 0.40718 |
| A_21_P0008779 | CRTC3-AS1 | 0.40766 |
| A_33_P3315814 | lnc-C2orf54-2 | 0.40806 |
| A_21_P0004337 | lnc-BNIP1-1 | 0.40898 |
| A_23_P428999 | NCOA2 | 0.40957 |
| A_21_P0012860 | LOC285626 | 0.41099 |
| A_21_P0005443 | LINC00996 | 0.41157 |
| A_33_P3832680 | FAM25C | 0.41196 |
| A_21_P0004313 | lnc-PPARGC1B-1 | 0.41216 |
| A_33_P3362267 | DGCR10 | 0.41223 |
| A_33_P3247331 | KCNQ1 | 0.41223 |
| A_33_P3334305 | TFF3 | 0.41268 |
| A_21_P0013262 | XLOC_l2_013554 | 0.41368 |
| A_23_P252882 | CSN3 | 0.41402 |
| A_21_P0005143 | lnc-QKI-2 | 0.41407 |
| A_21_P0007181 | lnc-INSC-3 | 0.41460 |
| A_21_P0011507 | XLOC_l2_005602 | 0.41503 |
| A_33_P3358863 | LOC100287823 | 0.41582 |
| A_33_P3229437 | KC6 | 0.41768 |
| A_33_P3255135 | LOC101929485 | 0.41794 |
| A_33_P3235207 | SLC38A4 | 0.41866 |
| A_32_P379379 | ATG9B | 0.41880 |
| A_21_P0001079 | lnc-C1orf31-7 | 0.41909 |
| A_32_P480177 | TNN | 0.41924 |
| A_24_P246091 | FLJ37201 | 0.42022 |
| A_24_P178503 | ABCC9 | 0.42050 |
| A_24_P337239 | FGF5 | 0.42115 |
| A_19_P00318638 | LINC00607 | 0.42146 |
| A_21_P0013091 | XLOC_l2_013116 | 0.42181 |
| A_33_P3251640 | LINC00663 | 0.42208 |
| A_21_P0009441 | lnc-SMCHD1-1 | 0.42302 |
| A_33_P3412493 | KY | 0.42341 |
| A_21_P0001667 | lnc-CLIC4-2 | 0.42402 |
| A_21_P0005953 | lnc-KHDRBS3-5 | 0.42413 |
| A_21_P0006517 | lnc-FAM127A-1 | 0.42465 |
| A_33_P3373384 | TMEM231 | 0.42486 |
| A_21_P0006003 | lnc-ANKRD18B-2 | 0.42518 |
| A_33_P3247175 | C4orf47 | 0.42524 |
| A_23_P421054 | C6orf165 | 0.42605 |
| A_23_P408787 | GCSAML | 0.42606 |
| A_21_P0001514 | lnc-JUN-6 | 0.42610 |
| A_21_P0013313 | XLOC_l2_013857 | 0.42643 |
| A_21_P0002261 | lnc-IL1R2-1 | 0.42730 |
| A_21_P0004301 | lnc-SLC25A48-2 | 0.42786 |
| A_21_P0007521 | lnc-CLEC4D-1 | 0.42819 |
| A_33_P3412428 | ADAM32 | 0.42828 |
| A_33_P3272437 | lnc-RBMY1J-1 | 0.42888 |
| A_23_P11103 | BMP15 | 0.42896 |
| A_21_P0010846 | XLOC_l2_001774 | 0.42939 |
| A_23_P143348 | OVOL2 | 0.43041 |
| A_21_P0000930 | LOC101929757 | 0.43068 |
| A_21_P0006425 | lnc-HEPH-2 | 0.43103 |
| A_21_P0012785 | lnc-COL25A1-1 | 0.43126 |
| A_23_P140074 | PROZ | 0.43169 |
| A_21_P0002899 | lnc-RP11-432B6.3.1-1 | 0.43192 |
| A_23_P366098 | NUP210L | 0.43210 |
| A_23_P7827 | FAM26F | 0.43221 |
| A_23_P129085 | SPESP1 | 0.43251 |
| A_19_P00811924 | lnc-GXYLT1-2 | 0.43282 |
| A_23_P208085 | MBP | 0.43296 |
| A_23_P154784 | BPIFB1 | 0.43365 |
| A_21_P0007931 | LINC00332 | 0.43397 |
| A_23_P137665 | CHI3L1 | 0.43407 |
| A_21_P0014697 | LOC101060542 | 0.43423 |
| A_23_P365218 | GPR110 | 0.43464 |
| A_21_P0007759 | lnc-MYL2-2 | 0.43496 |
| A_23_P2645 | SDS | 0.43504 |
| A_21_P0001574 | lnc-WARS2-2 | 0.43531 |
| A_21_P0005754 | lnc-UTP23-1 | 0.43565 |
| A_33_P3352283 | C1orf195 | 0.43595 |
| A_33_P3692766 | FLJ46066 | 0.43596 |
| A_24_P303420 | ADCY10P1 | 0.43689 |
| A_23_P325606 | ESX1 | 0.43743 |
| A_33_P3236676 | C9orf152 | 0.43802 |
| A_21_P0007141 | lnc-OR51B6-1 | 0.43885 |
| A_33_P3403658 | lnc-FBN1-3 | 0.43948 |
| A_23_P90980 | NEU2 | 0.43988 |
| A_33_P3408949 | LOXHD1 | 0.44010 |
| A_33_P3366161 | ABAT | 0.44101 |
| A_23_P24493 | MMP8 | 0.44151 |
| A_33_P3255459 | LOC100133461 | 0.44260 |
| A_21_P0013002 | XLOC_l2_012163 | 0.44399 |
| A_21_P0001001 | lnc-KCNC4-1 | 0.44450 |
| A_24_P208567 | IL18R1 | 0.44473 |
| A_33_P3216133 | ZMAT4 | 0.44490 |
| A_32_P128258 | SIGLEC17P | 0.44516 |
| A_21_P0000794 | LINC01220 | 0.44542 |
| A_21_P0005165 | lnc-MRPS18A-2 | 0.44559 |
| A_21_P0009653 | DKFZp434J0226 | 0.44638 |
| A_21_P0008852 | lnc-ITGA11-2 | 0.44661 |
| A_33_P3353662 | C9orf66 | 0.44679 |
| A_24_P49199 | GLDN | 0.44727 |
| A_21_P0004466 | lnc-LCP2-2 | 0.44729 |
| A_33_P3354723 | DEDD | 0.44753 |
| A_23_P391443 | PPM1H | 0.44810 |
| A_21_P0014346 | LOC101929767 | 0.44817 |
| A_23_P26062 | TMEM202 | 0.44867 |
| A_23_P133842 | HIST1H1T | 0.44877 |
| A_19_P00317760 | lnc-NR5A2-1 | 0.44937 |
| A_33_P3316318 | CEP120 | 0.44969 |
| A_21_P0010352 | LOC102724900 | 0.45043 |
| A_21_P0010255 | lnc-PDE9A-1 | 0.45161 |
| A_21_P0009358 | lnc-SLC39A11-1 | 0.45164 |
| A_21_P0006062 | lnc-PPP1R26-1 | 0.45183 |
| A_19_P00321053 | LINC01021 | 0.45241 |
| A_21_P0011088 | XLOC_l2_003293 | 0.45268 |
| A_21_P0004982 | lnc-EXOC2-1 | 0.45326 |
| A_19_P00317451 | XIST | 0.45348 |
| A_21_P0014889 | LOC100507431 | 0.45351 |
| A_24_P34611 | SIX3 | 0.45382 |
| A_19_P00317910 | LINC01503 | 0.45399 |
| A_23_P308042 | C6orf195 | 0.45411 |
| A_33_P3373046 | ZNF772 | 0.45418 |
| A_21_P0011008 | XLOC_l2_002651 | 0.45449 |
| A_21_P0000779 | SPTY2D1-AS1 | 0.45486 |
| A_21_P0012907 | XLOC_l2_012035 | 0.45532 |
| A_21_P0009088 | lnc-C16orf95-2 | 0.45539 |
| A_33_P3322230 | FLJ31945 | 0.45556 |
| A_21_P0012738 | LOC100507487 | 0.45640 |
| A_32_P16053 | MIR663AHG | 0.45678 |
| A_33_P3267059 | SLC9A1 | 0.45704 |
| A_21_P0012660 | XLOC_l2_011095 | 0.45757 |
| A_23_P135007 | OR2S2 | 0.45776 |
| A_21_P0003233 | LOC101929223 | 0.45782 |
| A_21_P0003780 | lnc-GYPA-1 | 0.45789 |
| A_24_P234838 | PCDH1 | 0.45808 |
| A_33_P3281730 | FKBP9 | 0.45873 |
| A_21_P0014651 | LOC100129203 | 0.45949 |
| A_33_P3413810 | LOC101928038 | 0.45962 |
| A_23_P149613 | FMO1 | 0.46029 |
| A_33_P3315390 | MPPED2 | 0.46140 |
| A_33_P3403595 | LOC100130976 | 0.46154 |
| A_23_P37359 | CIDEB | 0.46191 |
| A_21_P0009143 | LOC101928614 | 0.46230 |
| A_21_P0004651 | lnc-DAAM2-3 | 0.46251 |
| A_21_P0006489 | lnc-MID1IP1-3 | 0.46314 |
| A_33_P3308232 | FAM224A | 0.46406 |
| A_21_P0007602 | LOC102725323 | 0.46506 |
| A_21_P0002989 | lnc-CADM2-4 | 0.46541 |
| A_21_P0005780 | lnc-ZNF623-1 | 0.46542 |
| A_33_P3251065 | KLK13 | 0.46569 |
| A_23_P112859 | CST1 | 0.46580 |
| A_33_P3249746 | CYP3A5 | 0.46601 |
| A_33_P3322999 | C10orf105 | 0.46621 |
| A_24_P104407 | SYNM | 0.46662 |
| A_23_P315772 | KCND1 | 0.46681 |
| A_21_P0006967 | lnc-C10orf31-7 | 0.46682 |
| A_21_P0013249 | XLOC_l2_013485 | 0.46688 |
| A_21_P0013907 | TTTY10 | 0.46735 |
| A_21_P0007080 | lnc-EMX2-5 | 0.46745 |
| A_23_P169437 | LCN2 | 0.46783 |
| A_33_P3294277 | CYP4F3 | 0.46792 |
| A_21_P0009742 | lnc-PGLYRP2-1 | 0.46809 |
| A_33_P3358511 | IMPG1 | 0.46822 |
| A_23_P386442 | KIAA0087 | 0.46838 |
| A_21_P0012475 | XLOC_l2_010489 | 0.46850 |
| A_21_P0003428 | LOC101928081 | 0.46870 |
| A_21_P0010627 | XLOC_l2_000961 | 0.46888 |
| A_21_P0011523 | XLOC_l2_005692 | 0.46991 |
| A_21_P0000009 | LMNTD1 | 0.46996 |
| A_21_P0010551 | ANKRD20A9P | 0.47008 |
| A_21_P0006916 | lnc-HABP2-1 | 0.47009 |
| A_33_P3249589 | BCL11A | 0.47058 |
| A_21_P0009071 | LINC01227 | 0.47091 |
| A_21_P0006623 | GATA3-AS1 | 0.47102 |
| A_23_P167468 | PRLR | 0.47116 |
| A_24_P415680 | TNNI3K | 0.47128 |
| A_21_P0007666 | lnc-TMEM132C-6 | 0.47137 |
| A_33_P3251582 | LRRC16A | 0.47142 |
| A_21_P0003477 | lnc-BTC-3 | 0.47260 |
| A_21_P0003933 | LOC100506688 | 0.47329 |
| A_21_P0001445 | LOC339529 | 0.47359 |
| A_21_P0008439 | lnc-FERMT2-1 | 0.47381 |
| A_33_P3354214 | lnc-TNFRSF14-2 | 0.47392 |
| A_23_P402279 | VN1R2 | 0.47429 |
| A_33_P3394272 | LINC00473 | 0.47442 |
| A_33_P3362232 | lnc-CLCN3-3 | 0.47454 |
| A_24_P411121 | TNFRSF18 | 0.47475 |
| A_33_P3321303 | ETV3 | 0.47480 |
| A_33_P3401556 | CTLA4 | 0.47489 |
| A_21_P0003837 | lnc-LPHN3-2 | 0.47516 |
| A_33_P3315325 | ANKRD46 | 0.47546 |
| A_23_P60079 | ANGPT2 | 0.47548 |
| A_21_P0009809 | LINC00652 | 0.47556 |
| A_21_P0007663 | lnc-TMEM132C-6 | 0.47588 |
| A_21_P0010516 | XLOC_l2_000080 | 0.47612 |
| A_33_P3221019 | ZAN | 0.47626 |
| A_33_P3417751 | C1orf61 | 0.47634 |
| A_33_P3377609 | SPAM1 | 0.47647 |
| A_33_P3318530 | LOC441204 | 0.47656 |
| A_33_P3317412 | REXO1L2P | 0.47704 |
| A_21_P0001263 | lnc-VPS13D-1 | 0.47709 |
| A_23_P500000 | SCEL | 0.47763 |
| A_21_P0008726 | lnc-FSIP1-5 | 0.47775 |
| A_24_P417664 | CC2D2B | 0.47803 |
| A_21_P0004442 | lnc-C5orf63-2 | 0.47804 |
| A_21_P0013947 | CGB7 | 0.47814 |
| A_24_P156748 | SLC30A2 | 0.47826 |
| A_19_P00322942 | MIR143HG | 0.47851 |
| A_21_P0001681 | lnc-PRKAA2-2 | 0.47875 |
| A_21_P0001799 | LOC102724675 | 0.47902 |
| A_21_P0013702 | LOC101927575 | 0.47907 |
| A_21_P0001191 | LOC101928565 | 0.47913 |
| A_33_P3226192 | NR2E3 | 0.47953 |
| A_21_P0013028 | XLOC_l2_012605 | 0.48025 |
| A_21_P0005017 | lnc-TRIM27-15 | 0.48050 |
| A_21_P0008213 | lnc-COL4A1-2 | 0.48068 |
| A_33_P3411744 | EGOT | 0.48113 |
| A_33_P3336715 | GABRB2 | 0.48137 |
| A_21_P0002305 | lnc-GALNT5-1 | 0.48139 |
| A_21_P0013790 | XLOC_l2_015596 | 0.48177 |
| A_33_P3471466 | LRRC74A | 0.48218 |
| A_24_P6370 | C1orf110 | 0.48240 |
| A_21_P0010800 | LOC101927762 | 0.48245 |
| A_21_P0005888 | lnc-EXT1-4 | 0.48248 |
| A_23_P147665 | OLFML1 | 0.48278 |
| A_21_P0013531 | lnc-FBXO25-5 | 0.48281 |
| A_21_P0005392 | lnc-STAG3L4-1 | 0.48293 |
| A_33_P3216570 | MUC5AC | 0.48317 |
| A_33_P3285565 | CLDN3 | 0.48322 |
| A_33_P3217437 | GBP6 | 0.48324 |
| A_21_P0009207 | lnc-C17orf97-8 | 0.48352 |
| A_24_P2361 | CLCNKA | 0.48392 |
| A_21_P0006976 | LOC101929117 | 0.48392 |
| A_23_P58407 | UGT2B15 | 0.48412 |
| A_23_P147465 | PARK2 | 0.48416 |
| A_21_P0005311 | lnc-ZNF680-3 | 0.48419 |
| A_21_P0003755 | lnc-PITX2-2 | 0.48435 |
| A_33_P3270104 | FAM26D | 0.48439 |
| A_21_P0001570 | lnc-NGF-1 | 0.48443 |
| A_21_P0010625 | XLOC_l2_000945 | 0.48466 |
| A_23_P251412 | SCGN | 0.48482 |
| A_21_P0004975 | lnc-SMOC2-8 | 0.48483 |
| A_21_P0002494 | lnc-SLC35F5-2 | 0.48531 |
| A_24_P347378 | ALOX5AP | 0.48562 |
| A_21_P0006385 | lnc-C9orf156-2 | 0.48563 |
| A_23_P140797 | CDH8 | 0.48619 |
| A_21_P0003168 | LINC01471 | 0.48632 |
| A_23_P128362 | MYBPC1 | 0.48633 |
| A_21_P0013576 | XLOC_l2_014827 | 0.48645 |
| A_21_P0003164 | lnc-KLF15-3 | 0.48656 |
| A_21_P0011763 | XLOC_l2_007135 | 0.48660 |
| A_23_P155755 | CXCL6 | 0.48671 |
| A_33_P3281606 | KCNT1 | 0.48693 |
| A_33_P3394243 | C1orf101 | 0.48694 |
| A_21_P0008397 | lnc-EIF5-1 | 0.48695 |
| A_21_P0014580 | LOC100507195 | 0.48697 |
| A_23_P98147 | CPN1 | 0.48758 |
| A_23_P405885 | DPPA2 | 0.48786 |
| A_23_P364613 | HOGA1 | 0.48798 |
| A_21_P0005014 | lnc-HIST1H1A-1 | 0.48805 |
| A_32_P480330 | EYS | 0.48808 |
| A_33_P3282261 | XLOC_l2_000001 | 0.48811 |
| A_33_P3395804 | CFAP61 | 0.48811 |
| A_21_P0002594 | lnc-CRIPT-1 | 0.48826 |
| A_21_P0002780 | TM4SF1-AS1 | 0.48878 |
| A_21_P0009778 | LOC102723931 | 0.48878 |
| A_23_P312837 | GLIS3-AS1 | 0.48879 |
| A_33_P3392807 | TAF1B | 0.48914 |
| A_21_P0006975 | lnc-DNAJC1-1 | 0.48930 |
| A_32_P148538 | LPPR4 | 0.48940 |
| A_33_P3402313 | SLC9A4 | 0.48959 |
| A_33_P3420048 | CSRNP3 | 0.48963 |
| A_33_P3798268 | LINC00606 | 0.48976 |
| A_33_P3249125 | UNC45B | 0.48993 |
| A_21_P0010112 | lnc-FOXA2-3 | 0.49030 |
| A_21_P0007863 | LOC101930457 | 0.49063 |
| A_21_P0001946 | lnc-ITGA4-1 | 0.49130 |
| A_33_P3351316 | FAM198A | 0.49130 |
| A_21_P0002372 | lnc-SH3BP4-2 | 0.49147 |
| A_21_P0004452 | LOC102723929 | 0.49165 |
| A_21_P0008208 | lnc-EFNB2-5 | 0.49197 |
| A_33_P3227360 | DLX1 | 0.49250 |
| A_21_P0001611 | lnc-FMOD-2 | 0.49260 |
| A_21_P0013214 | XLOC_l2_013442 | 0.49333 |
| A_33_P3332215 | MUC1 | 0.49375 |
| A_33_P3349384 | LOC102724074 | 0.49378 |
| A_21_P0012900 | XLOC_l2_011987 | 0.49380 |
| A_33_P3351175 | WNK2 | 0.49390 |
| A_23_P432448 | ZNF205-AS1 | 0.49428 |
| A_21_P0008141 | lnc-HSPH1-6 | 0.49446 |
| A_21_P0006369 | LOC100288842 | 0.49459 |
| A_21_P0006614 | LOC101928298 | 0.49472 |
| A_33_P3293883 | lnc-SERINC5-1 | 0.49511 |
| A_21_P0004184 | lnc-PRELID2-1 | 0.49544 |
| A_33_P3270346 | KIR2DL5A | 0.49605 |
| A_21_P0007562 | lnc-HOXC4-4 | 0.49634 |
| A_21_P0008521 | lnc-CHGA-1 | 0.49639 |
| A_21_P0005698 | lnc-CHCHD7-2 | 0.49645 |
| A_21_P0012137 | LOC339593 | 0.49647 |
| A_21_P0009659 | lnc-SAFB-1 | 0.49659 |
| A_33_P3384202 | OR8D4 | 0.49674 |
| A_21_P0012916 | XLOC_l2_012135 | 0.49699 |
| A_33_P3284473 | lnc-ZNF454-1 | 0.49717 |
| A_21_P0005226 | lnc-ANLN-4 | 0.49719 |
| A_21_P0002186 | DNAJC27-AS1 | 0.49723 |
| A_24_P165205 | MORN1 | 0.49735 |
| A_21_P0010823 | XLOC_l2_001569 | 0.49762 |
| A_21_P0009991 | lnc-COL20A1-3 | 0.49783 |
| A_21_P0004843 | lnc-CD83-3 | 0.49800 |
| A_32_P232214 | TRABD2B | 0.49804 |
| A_24_P310256 | LGI4 | 0.49833 |
| A_23_P390545 | PCDHGA8 | 0.49857 |
| A_21_P0005367 | lnc-C7orf41-1 | 0.49860 |
| A_33_P3231319 | XLOC_l2_013192 | 0.49874 |
| A_23_P423309 | PCDH12 | 0.49881 |
| A_23_P117464 | TPPP2 | 0.49882 |
| A_33_P3226385 | lnc-EFR3B-2 | 0.49894 |
| A_21_P0003775 | lnc-SLC7A11-1 | 0.49906 |
| A_23_P79769 | BIRC7 | 0.49908 |
| A_33_P3367196 | CNTNAP2 | 0.49937 |
| A_21_P0008181 | lnc-KCTD12-1 | 0.49944 |
| A_33_P3334275 | lnc-LIF-1 | 0.49960 |
| A_33_P3211198 | NCMAP | 0.49970 |
| A_33_P3351279 | GPR37L1 | 0.49980 |

*Fold change shows the expression level for H2052 treated by anti-S100A11 antibody relative to parental H2052.

(C) Up-regulated genes in H2452 treated by anti-S100A11 antibody compared to parental H2452.

| **ProbeName** | **GeneSymbol** | **Fold change** |
| --- | --- | --- |
| A_21_P0007313 | lnc-CDKN1C-1 | 5.005 |
| A_33_P3394853 | LOC100132215 | 3.931 |
| A_33_P3348872 | PRDM16 | 3.880 |
| A_33_P3347281 | HNMT | 3.738 |
| A_24_P942441 | NRXN1 | 3.726 |
| A_21_P0002142 | SPATA3-AS1 | 3.691 |
| A_21_P0012800 | LINC00616 | 3.673 |
| A_23_P126363 | ADAM30 | 3.643 |
| A_21_P0007160 | LOC101928443 | 3.620 |
| A_21_P0007613 | lnc-C12orf48-3 | 3.580 |
| A_21_P0010579 | XLOC_l2_000643 | 3.564 |
| A_21_P0002251 | lnc-ATOH8-2 | 3.499 |
| A_21_P0013211 | XLOC_l2_013437 | 3.491 |
| A_23_P51580 | HSD3B2 | 3.479 |
| A_21_P0006811 | lnc-PFKP-9 | 3.461 |
| A_21_P0004526 | lnc-PRR16-1 | 3.447 |
| A_23_P29965 | SMR3B | 3.426 |
| A_33_P3280561 | KRTAP16-1 | 3.421 |
| A_21_P0007944 | LINC00383 | 3.393 |
| A_21_P0002991 | lnc-CADM2-3 | 3.352 |
| A_21_P0005885 | lnc-KCNV1-2 | 3.340 |
| A_21_P0006628 | LOC101927419 | 3.328 |
| A_23_P129133 | OCA2 | 3.287 |
| A_21_P0010509 | LINC01134 | 3.283 |
| A_21_P0011749 | XLOC_l2_007059 | 3.244 |
| A_23_P345799 | FAM129C | 3.232 |
| A_21_P0012176 | XLOC_l2_009096 | 3.226 |
| A_21_P0004494 | lnc-CDH6-2 | 3.213 |
| A_21_P0010365 | LOC101927526 | 3.180 |
| A_21_P0005964 | lnc-ZMAT4-3 | 3.173 |
| A_33_P3306679 | PLEKHG7 | 3.164 |
| A_23_P86975 | CARD18 | 3.164 |
| A_21_P0006780 | lnc-ANKRD1-1 | 3.137 |
| A_21_P0011633 | KRT14 | 3.134 |
| A_23_P42931 | CLEC2L | 3.129 |
| A_21_P0010418 | LOC101927344 | 3.123 |
| A_21_P0004649 | lnc-DNAH8-1 | 3.108 |
| A_21_P0002513 | lnc-RND3-4 | 3.108 |
| A_21_P0011331 | CSPG4 | 3.097 |
| A_33_P3404974 | OPN5 | 3.089 |
| A_23_P143774 | MOV10L1 | 3.045 |
| A_21_P0000486 | SNORD103A | 3.030 |
| A_33_P3246593 | NUGGC | 3.009 |
| A_24_P16833 | FAT3 | 3.003 |
| A_21_P0002950 | lnc-ZNF621-1 | 3.001 |
| A_21_P0006244 | LOC102724193 | 2.994 |
| A_21_P0001750 | lnc-UCHL5-1 | 2.979 |
| A_21_P0014868 | LOC100508046 | 2.966 |
| A_21_P0009048 | lnc-TK2-5 | 2.966 |
| A_21_P0006688 | LINC00858 | 2.956 |
| A_33_P3358923 | BTLA | 2.946 |
| A_21_P0007332 | LOC102723403 | 2.943 |
| A_23_P421379 | IGF2 | 2.925 |
| A_21_P0005764 | lnc-PHF20L1-2 | 2.924 |
| A_33_P3340260 | OTOG | 2.919 |
| A_21_P0011703 | XLOC_l2_006789 | 2.915 |
| A_33_P3423959 | DBX2 | 2.911 |
| A_32_P141682 | EVPLL | 2.909 |
| A_33_P3327608 | EML6 | 2.902 |
| A_33_P3368905 | LINC01138 | 2.901 |
| A_33_P3403831 | XLOC_l2_015399 | 2.900 |
| A_23_P29394 | ATP13A4 | 2.886 |
| A_32_P206899 | DNAH2 | 2.876 |
| A_21_P0011953 | PGM5P3-AS1 | 2.873 |
| A_23_P71855 | C5 | 2.873 |
| A_21_P0005998 | PTPRD-AS2 | 2.869 |
| A_32_P385667 | MAGEB10 | 2.856 |
| A_24_P211420 | SPEF1 | 2.856 |
| A_21_P0012091 | FAR2P2 | 2.852 |
| A_23_P346390 | CXorf36 | 2.845 |
| A_23_P145159 | DNAH8 | 2.840 |
| A_23_P128323 | SCNN1A | 2.834 |
| A_21_P0001523 | lnc-TTLL7-5 | 2.834 |
| A_23_P17316 | NKAIN4 | 2.822 |
| A_21_P0000822 | LOC286189 | 2.792 |
| A_21_P0009378 | LINC00670 | 2.776 |
| A_33_P3316485 | LOC100129603 | 2.769 |
| A_23_P42386 | CGA | 2.767 |
| A_24_P408736 | GALNT5 | 2.765 |
| A_21_P0004547 | lnc-HNRNPAB-2 | 2.764 |
| A_23_P163737 | OR2C1 | 2.756 |
| A_23_P140475 | NOX5 | 2.747 |
| A_24_P231302 | PGLYRP4 | 2.746 |
| A_21_P0001367 | lnc-WDR3-2 | 2.737 |
| A_21_P0003315 | LINC00955 | 2.736 |
| A_21_P0007012 | lnc-ANKRD1-2 | 2.729 |
| A_21_P0008212 | lnc-IRS2-1 | 2.725 |
| A_21_P0002764 | lnc-GAP43-5 | 2.719 |
| A_33_P3341259 | LEAP2 | 2.714 |
| A_21_P0011871 | LOC100996693 | 2.695 |
| A_21_P0011606 | XLOC_l2_006166 | 2.687 |
| A_24_P247902 | PCLO | 2.686 |
| A_32_P206735 | PRO1082 | 2.676 |
| A_23_P153797 | NCAN | 2.665 |
| A_21_P0007662 | lnc-TMEM132C-7 | 2.662 |
| A_21_P0010477 | lnc-SYN3-1 | 2.657 |
| A_21_P0008162 | lnc-RCBTB2-1 | 2.657 |
| A_21_P0006113 | lnc-C9orf104-2 | 2.654 |
| A_33_P3352906 | KCNIP4-IT1 | 2.636 |
| A_21_P0005301 | lnc-H2AFV-1 | 2.635 |
| A_21_P0002954 | LOC100506319 | 2.634 |
| A_33_P3416867 | lnc-PRPF18-5 | 2.632 |
| A_21_P0011188 | LINC00448 | 2.629 |
| A_24_P120251 | TM4SF18 | 2.606 |
| A_21_P0005931 | lnc-PPP3CC-1 | 2.606 |
| A_21_P0004201 | lnc-C5orf38-1 | 2.603 |
| A_23_P430411 | ITGB2 | 2.601 |
| A_33_P3417081 | ABCA17P | 2.597 |
| A_33_P3328782 | DKFZP434L187 | 2.585 |
| A_24_P342829 | SLC16A14 | 2.573 |
| A_23_P252541 | RAB7B | 2.566 |
| A_21_P0005465 | lnc-AC091801.1.1-5 | 2.565 |
| A_21_P0011830 | XLOC_l2_007452 | 2.564 |
| A_21_P0004777 | LOC101928326 | 2.561 |
| A_21_P0000764 | LINC01364 | 2.557 |
| A_21_P0004591 | MLLT4-AS1 | 2.556 |
| A_24_P400324 | THSD7A | 2.553 |
| A_24_P276873 | ERMN | 2.548 |
| A_23_P109488 | PIK3IP1 | 2.547 |
| A_21_P0004220 | LOC102724943 | 2.546 |
| A_23_P202810 | OVOL1 | 2.541 |
| A_21_P0004973 | lnc-SMOC2-5 | 2.534 |
| A_21_P0013827 | XLOC_l2_015789 | 2.533 |
| A_21_P0006119 | LOC102723803 | 2.533 |
| A_21_P0009426 | LOC101927229 | 2.527 |
| A_23_P381172 | MRAP | 2.521 |
| A_24_P208081 | PLA2G2F | 2.511 |
| A_24_P15586 | THAP5 | 2.510 |
| A_21_P0006424 | lnc-FAAH2-2 | 2.508 |
| A_33_P3400286 | lnc-KAT7-4 | 2.497 |
| A_23_P337642 | ATP2B3 | 2.495 |
| A_21_P0008547 | lnc-RP11-204N11.1.1-3 | 2.491 |
| A_33_P3298460 | GCG | 2.472 |
| A_21_P0010299 | lnc-ICOSLG-3 | 2.467 |
| A_21_P0012892 | XLOC_l2_011954 | 2.464 |
| A_21_P0001057 | LOC91548 | 2.462 |
| A_21_P0005192 | lnc-GTF2I-1 | 2.457 |
| A_21_P0001161 | LINC01349 | 2.451 |
| A_21_P0005382 | lnc-POM121L12-2 | 2.448 |
| A_24_P330822 | HNF1B | 2.443 |
| A_33_P3373134 | SZT2 | 2.442 |
| A_23_P66854 | KRT20 | 2.435 |
| A_33_P3641456 | LOC642422 | 2.435 |
| A_21_P0007903 | lnc-SLC15A4-2 | 2.431 |
| A_33_P3237664 | HDAC7 | 2.430 |
| A_33_P3570218 | EHHADH-AS1 | 2.430 |
| A_23_P349398 | ZAR1 | 2.430 |
| A_23_P12533 | ANKRD30A | 2.427 |
| A_21_P0012131 | MIR646HG | 2.420 |
| A_23_P67661 | COX7A1 | 2.419 |
| A_33_P3304691 | KRTAP5-1 | 2.407 |
| A_21_P0013810 | XLOC_l2_015700 | 2.404 |
| A_33_P3329433 | TTN | 2.402 |
| A_23_P42353 | ETV7 | 2.398 |
| A_21_P0005556 | lnc-EPDR1-1 | 2.393 |
| A_21_P0013851 | ARMCX4 | 2.383 |
| A_21_P0006354 | lnc-FAM75D1-4 | 2.382 |
| A_23_P137484 | L1TD1 | 2.382 |
| A_21_P0010614 | XLOC_l2_000910 | 2.377 |
| A_23_P420359 | ADAMTS20 | 2.372 |
| A_24_P822692 | CCER2 | 2.371 |
| A_33_P3233378 | LOC101060157 | 2.366 |
| A_33_P3304883 | LOC100131372 | 2.365 |
| A_33_P3351745 | PVRIG | 2.365 |
| A_33_P3236122 | lnc-C1orf31-1 | 2.363 |
| A_21_P0007832 | MUC19 | 2.362 |
| A_21_P0002581 | lnc-SLC4A1AP-1 | 2.357 |
| A_21_P0014669 | ZNF518A | 2.354 |
| A_24_P257478 | COL25A1 | 2.351 |
| A_32_P92489 | PKD1L2 | 2.347 |
| A_21_P0005046 | lnc-HACE1-4 | 2.341 |
| A_21_P0012931 | XLOC_l2_012323 | 2.341 |
| A_21_P0005146 | lnc-FAM120B-6 | 2.339 |
| A_23_P128281 | KLRC3 | 2.338 |
| A_21_P0002997 | lnc-C3orf38-2 | 2.331 |
| A_33_P3259587 | SCAND2P | 2.329 |
| A_21_P0004623 | lnc-AL035696.1-3 | 2.326 |
| A_21_P0007707 | lnc-C1QL4-2 | 2.324 |
| A_21_P0009054 | lnc-PMFBP1-2 | 2.324 |
| A_21_P0006168 | lnc-C9orf170-2 | 2.318 |
| A_21_P0002320 | lnc-HNRNPA3-4 | 2.316 |
| A_23_P34700 | TNNT2 | 2.316 |
| A_33_P3413394 | ZACN | 2.314 |
| A_21_P0007240 | lnc-USP35-1 | 2.305 |
| A_21_P0011608 | XLOC_l2_006173 | 2.304 |
| A_21_P0012221 | ANKRD20A11P | 2.302 |
| A_33_P3408514 | SCNN1D | 2.291 |
| A_24_P94402 | MYCN | 2.290 |
| A_21_P0011338 | LINC00933 | 2.289 |
| A_23_P17844 | PVALB | 2.284 |
| A_21_P0009657 | LYPD5 | 2.283 |
| A_23_P344555 | NEDD9 | 2.283 |
| A_21_P0001729 | lnc-DMRTA2-1 | 2.281 |
| A_33_P3420635 | OR4D6 | 2.280 |
| A_33_P3872301 | SNAR-C3 | 2.278 |
| A_33_P3364433 | ACVR1B | 2.276 |
| A_21_P0005201 | LOC101928211 | 2.275 |
| A_33_P3220113 | ZNF816-ZNF321P | 2.274 |
| A_24_P365721 | SLC6A14 | 2.274 |
| A_23_P388331 | BAGE | 2.273 |
| A_19_P00801945 | LOC441204 | 2.273 |
| A_33_P3379881 | FMN1 | 2.273 |
| A_33_P3741022 | LINC00511 | 2.272 |
| A_33_P3318606 | SHC2 | 2.266 |
| A_19_P00809895 | LINC01314 | 2.265 |
| A_24_P66605 | SLITRK2 | 2.263 |
| A_21_P0005234 | LINC01445 | 2.260 |
| A_33_P3397603 | LOC100130278 | 2.256 |
| A_33_P3250165 | ERMN | 2.254 |
| A_24_P150466 | SMOC1 | 2.252 |
| A_21_P0007738 | lnc-C12orf12-1 | 2.242 |
| A_21_P0007847 | lnc-NEDD1-5 | 2.241 |
| A_33_P3279847 | RAET1E | 2.239 |
| A_33_P3352632 | LOC728147 | 2.239 |
| A_24_P124624 | OLR1 | 2.234 |
| A_23_P116430 | USH1C | 2.232 |
| A_23_P383679 | HYDIN | 2.232 |
| A_21_P0002397 | LOC101929861 | 2.231 |
| A_21_P0005748 | lnc-TTC35-3 | 2.231 |
| A_33_P3230841 | DUPD1 | 2.230 |
| A_21_P0011776 | LOC100420587 | 2.230 |
| A_19_P00319398 | ERVMER34-1 | 2.229 |
| A_21_P0008326 | LOC102723503 | 2.227 |
| A_24_P398147 | NEBL | 2.226 |
| A_21_P0002799 | LINC01330 | 2.226 |
| A_33_P3285815 | WFDC6 | 2.226 |
| A_21_P0013804 | XLOC_l2_015661 | 2.225 |
| A_21_P0002579 | lnc-KLHL29-2 | 2.224 |
| A_21_P0000535 | LINC00173 | 2.224 |
| A_23_P368794 | TCERG1L | 2.223 |
| A_21_P0002036 | LOC100505774 | 2.221 |
| A_21_P0005259 | LOC100506682 | 2.217 |
| A_24_P401870 | C9orf139 | 2.214 |
| A_21_P0001924 | lnc-ACVR2A-1 | 2.212 |
| A_21_P0009985 | lnc-C20orf197-1 | 2.212 |
| A_23_P157879 | FCN1 | 2.208 |
| A_23_P169437 | LCN2 | 2.208 |
| A_33_P3292769 | NFAM1 | 2.202 |
| A_33_P3217609 | TBC1D22A | 2.199 |
| A_23_P43107 | DCSTAMP | 2.197 |
| A_21_P0006470 | lnc-MAGEA12-1 | 2.197 |
| A_33_P3363680 | XLOC_l2_000018 | 2.196 |
| A_23_P381505 | VWDE | 2.196 |
| A_24_P925635 | SEPT7P2 | 2.196 |
| A_21_P0007642 | lnc-SRRM4-1 | 2.194 |
| A_24_P246841 | SLC25A27 | 2.194 |
| A_33_P3241244 | LOC100131822 | 2.191 |
| A_23_P381489 | LINC00313 | 2.189 |
| A_33_P3353547 | ZBTB8B | 2.181 |
| A_23_P155900 | NPFFR2 | 2.181 |
| A_33_P3291524 | ZNF534 | 2.174 |
| A_32_P405973 | ADCY2 | 2.169 |
| A_24_P62783 | FABP3 | 2.169 |
| A_33_P3354723 | DEDD | 2.169 |
| A_21_P0007960 | lnc-FARP1-1 | 2.167 |
| A_21_P0007440 | LOC102724209 | 2.166 |
| A_23_P211417 | RFPL1 | 2.165 |
| A_21_P0004905 | lnc-TPBG-1 | 2.163 |
| A_21_P0014846 | SRGAP3-AS3 | 2.160 |
| A_33_P3246133 | HPCA | 2.160 |
| A_21_P0009213 | lnc-MIS12-1 | 2.157 |
| A_21_P0007072 | lnc-PGAM1-1 | 2.157 |
| A_21_P0001726 | lnc-SLC6A9-3 | 2.157 |
| A_23_P35055 | NPHS2 | 2.156 |
| A_24_P657226 | SLC9B1 | 2.156 |
| A_33_P3385161 | EFCAB9 | 2.154 |
| A_21_P0010312 | lnc-WRB-2 | 2.153 |
| A_23_P130281 | OR1A2 | 2.151 |
| A_21_P0008057 | lnc-TNFSF11-1 | 2.151 |
| A_21_P0010692 | MST1P2 | 2.149 |
| A_33_P3218089 | CLDN24 | 2.149 |
| A_33_P3229918 | PTCRA | 2.147 |
| A_23_P430558 | CHRND | 2.146 |
| A_33_P3489675 | ARHGAP8 | 2.144 |
| A_21_P0001233 | lnc-LBR-1 | 2.143 |
| A_23_P108082 | CREB3L3 | 2.142 |
| A_21_P0001036 | LINC01036 | 2.142 |
| A_23_P83098 | ALDH1A1 | 2.141 |
| A_21_P0008272 | LOC100506999 | 2.141 |
| A_21_P0003577 | lnc-CPEB2-7 | 2.141 |
| A_21_P0009801 | LOC102723379 | 2.141 |
| A_23_P85534 | GPR52 | 2.141 |
| A_33_P3422043 | PRSS48 | 2.139 |
| A_19_P00322997 | LOC101928682 | 2.139 |
| A_32_P146113 | ARMC12 | 2.138 |
| A_33_P3261947 | SNTG1 | 2.134 |
| A_21_P0007887 | LOC643770 | 2.133 |
| A_23_P78867 | RYR1 | 2.133 |
| A_33_P3366175 | LOC100130298 | 2.133 |
| A_33_P3328559 | TBC1D10C | 2.133 |
| A_21_P0010244 | lnc-ETS2-4 | 2.132 |
| A_33_P3403658 | lnc-FBN1-3 | 2.131 |
| A_23_P53884 | MAB21L1 | 2.130 |
| A_33_P3245679 | LOC100129940 | 2.130 |
| A_21_P0008483 | LINC01550 | 2.129 |
| A_33_P3306103 | CALCRL | 2.128 |
| A_21_P0011882 | MROH2A | 2.127 |
| A_21_P0002877 | lnc-TMCC1-1 | 2.126 |
| A_21_P0003780 | lnc-GYPA-1 | 2.125 |
| A_19_P00803334 | lnc-GALNTL4-2 | 2.124 |
| A_23_P88177 | SERPINA12 | 2.124 |
| A_33_P3264667 | CYP26C1 | 2.123 |
| A_21_P0009162 | CCDC144NL-AS1 | 2.123 |
| A_24_P367645 | MAP7D2 | 2.118 |
| A_21_P0003001 | lnc-OR5H15-1 | 2.117 |
| A_21_P0004855 | lnc-NRSN1-1 | 2.116 |
| A_24_P326398 | CRB2 | 2.115 |
| A_21_P0002137 | LOC102724675 | 2.114 |
| A_21_P0001796 | lnc-FAM168B-1 | 2.111 |
| A_21_P0007808 | lnc-RIMBP2-2 | 2.110 |
| A_21_P0002683 | lnc-DLX2-1 | 2.109 |
| A_33_P3382100 | IGFN1 | 2.107 |
| A_33_P3246935 | CSMD2 | 2.104 |
| A_24_P926507 | SLC14A1 | 2.104 |
| A_23_P375419 | TTC16 | 2.102 |
| A_33_P3401477 | lnc-WNT8A-2 | 2.095 |
| A_21_P0006589 | LINC00278 | 2.091 |
| A_21_P0013455 | ZNF767P | 2.090 |
| A_21_P0009796 | lnc-ZNF71-3 | 2.088 |
| A_32_P527371 | SGSM1 | 2.088 |
| A_33_P3255459 | LOC100133461 | 2.087 |
| A_21_P0008149 | lnc-C13orf44-1 | 2.086 |
| A_23_P334246 | APLF | 2.084 |
| A_33_P3362267 | DGCR10 | 2.083 |
| A_21_P0008826 | lnc-UNC13C-2 | 2.079 |
| A_21_P0006246 | lnc-CACNA1B-1 | 2.077 |
| A_33_P3423984 | PALLD | 2.076 |
| A_24_P13285 | PPP1R1A | 2.075 |
| A_33_P3397716 | lnc-GGCT-1 | 2.075 |
| A_33_P3214925 | MIB2 | 2.074 |
| A_33_P3374076 | SEZ6 | 2.074 |
| A_33_P3423954 | CBX2 | 2.073 |
| A_23_P60009 | ANXA13 | 2.073 |
| A_21_P0007179 | lnc-INSC-2 | 2.073 |
| A_21_P0006139 | lnc-PRSS3-1 | 2.072 |
| A_33_P3354823 | BTBD19 | 2.072 |
| A_21_P0008749 | lnc-UBAP1L-1 | 2.071 |
| A_33_P3230189 | SLITRK6 | 2.071 |
| A_32_P197825 | PLGLB1 | 2.070 |
| A_23_P324813 | BCL6B | 2.069 |
| A_21_P0011322 | STARD9 | 2.068 |
| A_32_P515431 | LOC100132057 | 2.067 |
| A_21_P0009497 | lnc-SOCS6-4 | 2.067 |
| A_23_P67453 | TNNI3 | 2.067 |
| A_33_P3280147 | FLJ45079 | 2.066 |
| A_33_P3369979 | OR2L2 | 2.066 |
| A_23_P129367 | DRC7 | 2.064 |
| A_19_P00320722 | SRP14-AS1 | 2.064 |
| A_21_P0006754 | LOC101929465 | 2.064 |
| A_23_P46829 | FGF8 | 2.064 |
| A_21_P0013876 | XLOC_l2_015879 | 2.061 |
| A_21_P0006146 | LOC101927042 | 2.061 |
| A_21_P0009617 | LINC00908 | 2.056 |
| A_21_P0013119 | XLOC_l2_013233 | 2.055 |
| A_21_P0001650 | lnc-PLD5-2 | 2.055 |
| A_21_P0002233 | lnc-MEIS1-3 | 2.055 |
| A_21_P0004432 | lnc-SEMA6A-5 | 2.052 |
| A_33_P3398513 | LOC728819 | 2.052 |
| A_33_P3278392 | NFU1 | 2.050 |
| A_21_P0001464 | LOC100506022 | 2.050 |
| A_21_P0005188 | HOXA11-AS | 2.050 |
| A_24_P162226 | RIMBP2 | 2.050 |
| A_21_P0011920 | XLOC_l2_008031 | 2.049 |
| A_21_P0007241 | lnc-USP35-1 | 2.047 |
| A_33_P3384058 | WNT9A | 2.045 |
| A_24_P302685 | ARHGEF4 | 2.044 |
| A_33_P3290582 | LINC01255 | 2.044 |
| A_23_P134729 | RBM12B-AS1 | 2.043 |
| A_24_P73535 | C14orf119 | 2.042 |
| A_33_P3334773 | MYOD1 | 2.041 |
| A_23_P92909 | SPINK6 | 2.039 |
| A_21_P0008491 | LINC00524 | 2.038 |
| A_21_P0003693 | lnc-F11-3 | 2.038 |
| A_32_P461386 | CCDC36 | 2.037 |
| A_19_P00320343 | LOC100507403 | 2.036 |
| A_21_P0010612 | TRABD2B | 2.034 |
| A_21_P0010139 | lnc-AF127577.1-4 | 2.033 |
| A_21_P0012269 | XLOC_l2_009358 | 2.032 |
| A_21_P0006219 | lnc-RP11-295D22.1.1-6 | 2.031 |
| A_33_P3220813 | TXLNB | 2.031 |
| A_21_P0013142 | ERVH-3 | 2.030 |
| A_33_P3347697 | NOVA1 | 2.030 |
| A_21_P0013524 | OTUD6B-AS1 | 2.030 |
| A_24_P303420 | ADCY10P1 | 2.029 |
| A_21_P0011153 | XLOC_l2_003803 | 2.029 |
| A_21_P0002514 | lnc-RPRM-4 | 2.027 |
| A_24_P296587 | DLX3 | 2.027 |
| A_21_P0002479 | lnc-TGFBRAP1-11 | 2.026 |
| A_33_P3311373 | LOC100506747 | 2.026 |
| A_21_P0002560 | lnc-AC131097.4.1-3 | 2.026 |
| A_21_P0004720 | lnc-C6orf146-1 | 2.025 |
| A_21_P0007211 | lnc-SERPING1-1 | 2.024 |
| A_21_P0013200 | XLOC_l2_013416 | 2.024 |
| A_33_P3372004 | IGSF6 | 2.024 |
| A_21_P0008799 | lnc-PGPEP1L-1 | 2.022 |
| A_21_P0010718 | lnc-RP3-377D14.1.1-3 | 2.022 |
| A_33_P3257891 | GSTA3 | 2.017 |
| A_21_P0003880 | lnc-FRG1-4 | 2.016 |
| A_21_P0014361 | lnc-C17orf75-3 | 2.014 |
| A_23_P372478 | SERPINA9 | 2.013 |
| A_21_P0011865 | XLOC_l2_007595 | 2.013 |
| A_33_P3385371 | NAP1L6 | 2.012 |
| A_21_P0010326 | lnc-ICOSLG-6 | 2.012 |
| A_23_P252882 | CSN3 | 2.011 |
| A_33_P3404281 | LOC51145 | 2.010 |
| A_21_P0003049 | lnc-GMPS-1 | 2.008 |
| A_33_P3351681 | CCDC67 | 2.006 |
| A_33_P3282740 | LINC00869 | 2.006 |
| A_33_P3250830 | C1orf110 | 2.006 |
| A_21_P0002968 | lnc-SLC25A26-1 | 2.004 |
| A_33_P3302657 | TLE4 | 2.004 |
| A_19_P00320229 | FLJ46906 | 2.002 |
| A_21_P0014059 | PIGC | 2.002 |
| A_21_P0003348 | LOC101927363 | 2.002 |
| A_23_P84736 | CTNNA2 | 2.001 |
| A_24_P541831 | LINC00264 | 2.001 |
| A_21_P0010183 | LINC00320 | 2.000 |
| A_23_P83683 | MORC1 | 2.000 |

*Fold change shows the expression level for H2452 treated by anti-S100A11 antibody relative to parental H2452.

(D) Down-regulated genes in H2452 treated by anti-S100A11 antibody compared to parental H2452.

| **ProbeName** | **GeneSymbol** | **Fold change** |
| --- | --- | --- |
| A_19_P00809548 | LINC00265 | 0.19379 |
| A_33_P3258206 | OR6N2 | 0.20875 |
| A_23_P417951 | TSPYL5 | 0.20991 |
| A_33_P3315944 | FIGLA | 0.21411 |
| A_21_P0009403 | lnc-LGALS9B-2 | 0.21867 |
| A_21_P0013022 | LOC101929309 | 0.22088 |
| A_32_P188825 | TDRD12 | 0.22143 |
| A_23_P168357 | CPA1 | 0.22231 |
| A_21_P0005501 | lnc-SBDS-1 | 0.22281 |
| A_32_P149546 | CROCC | 0.22319 |
| A_19_P00802872 | XIST | 0.22367 |
| A_21_P0002026 | LOC101928196 | 0.22494 |
| A_33_P3236656 | SPACA4 | 0.22543 |
| A_21_P0010122 | lnc-ZFP64-1 | 0.22606 |
| A_33_P3218980 | ENTPD1 | 0.22608 |
| A_21_P0004619 | KIF25-AS1 | 0.22679 |
| A_21_P0004597 | lnc-GPX5-1 | 0.22698 |
| A_21_P0004515 | LOC100505878 | 0.22964 |
| A_21_P0008725 | LOC102724253 | 0.23142 |
| A_21_P0008941 | lnc-CHD9-7 | 0.23183 |
| A_21_P0013115 | XLOC_l2_013192 | 0.23303 |
| A_21_P0005315 | lnc-MAGI2-1 | 0.23349 |
| A_24_P206317 | HDAC9 | 0.23607 |
| A_21_P0005874 | lnc-C8orf37-1 | 0.23800 |
| A_33_P3300027 | lnc-ACOT12-2 | 0.24068 |
| A_33_P3368991 | MPZ | 0.24194 |
| A_21_P0001357 | lnc-AMY1C-2 | 0.24305 |
| A_21_P0000458 | SNORD115-28 | 0.24315 |
| A_24_P246196 | CLEC4M | 0.24380 |
| A_24_P144601 | POU5F1 | 0.24445 |
| A_32_P34046 | HFM1 | 0.24482 |
| A_32_P332551 | FAM216B | 0.24662 |
| A_33_P3242069 | LOC729159 | 0.24736 |
| A_21_P0009800 | lnc-AC008686.1-1 | 0.24752 |
| A_21_P0013585 | LOC102724462 | 0.24794 |
| A_33_P3262660 | HSFY1P1 | 0.24848 |
| A_33_P3367037 | lnc-SDR42E1-1 | 0.24930 |
| A_21_P0002249 | lnc-DNAH6-2 | 0.25024 |
| A_33_P3230364 | OR52E2 | 0.25025 |
| A_21_P0008928 | lnc-LONP2-2 | 0.25087 |
| A_21_P0002525 | lnc-PDE1A-1 | 0.25159 |
| A_33_P3273777 | GJB7 | 0.25292 |
| A_21_P0010107 | lnc-GPCPD1-1 | 0.25323 |
| A_32_P132860 | UNQ6494 | 0.25327 |
| A_33_P3539684 | LOC153910 | 0.25359 |
| A_21_P0007827 | LOC101928705 | 0.25481 |
| A_21_P0008779 | CRTC3-AS1 | 0.25570 |
| A_21_P0013754 | XLOC_l2_015453 | 0.25588 |
| A_24_P280664 | GBP7 | 0.25644 |
| A_21_P0002518 | lnc-STK39-1 | 0.25668 |
| A_32_P320247 | NEUROD4 | 0.25703 |
| A_32_P111639 | CHST9 | 0.25713 |
| A_33_P3280346 | LOC100130857 | 0.25803 |
| A_23_P45592 | TTTY8 | 0.25862 |
| A_33_P3423626 | LOC151121 | 0.25865 |
| A_21_P0001621 | lnc-C1orf227-1 | 0.26038 |
| A_23_P356163 | WDR49 | 0.26039 |
| A_19_P00809030 | LOC101929378 | 0.26078 |
| A_23_P153155 | GALR1 | 0.26160 |
| A_21_P0002617 | lnc-SLC4A10-4 | 0.26171 |
| A_21_P0014242 | lnc-DENND1A-3 | 0.26185 |
| A_21_P0000633 | IGFBP7-AS1 | 0.26404 |
| A_23_P312383 | C5orf64 | 0.26413 |
| A_33_P3221748 | RUNX3 | 0.26448 |
| A_21_P0001499 | lnc-STIL-3 | 0.26452 |
| A_21_P0008494 | lnc-HSP90AA1-3 | 0.26467 |
| A_21_P0008666 | lnc-SH3GL3-1 | 0.26501 |
| A_21_P0011245 | LRRC9 | 0.26519 |
| A_33_P3346801 | BMF | 0.26617 |
| A_21_P0010497 | HOTTIP | 0.26679 |
| A_21_P0007834 | LOC101927038 | 0.26694 |
| A_21_P0002636 | lnc-FAM150B-1 | 0.26807 |
| A_32_P210872 | HEPN1 | 0.26817 |
| A_21_P0010392 | lnc-AC007663.1-5 | 0.26911 |
| A_23_P380990 | CLEC4F | 0.26934 |
| A_23_P152906 | ALOX12 | 0.26962 |
| A_24_P235988 | CLEC7A | 0.26965 |
| A_21_P0013063 | LOC101928253 | 0.26990 |
| A_33_P3276678 | VSX1 | 0.27014 |
| A_33_P3415653 | LOC100128668 | 0.27057 |
| A_19_P00321953 | LINC01118 | 0.27060 |
| A_24_P11900 | MYH15 | 0.27122 |
| A_24_P237936 | TCF23 | 0.27279 |
| A_23_P81683 | GPRC6A | 0.27336 |
| A_24_P97374 | EOMES | 0.27369 |
| A_23_P302060 | IFNE | 0.27531 |
| A_23_P89132 | ZP2 | 0.27624 |
| A_33_P3273669 | NRXN1 | 0.27683 |
| A_21_P0000455 | SNORD116-28 | 0.27730 |
| A_23_P82651 | NPTX2 | 0.27738 |
| A_21_P0009359 | lnc-SLC39A11-1 | 0.27781 |
| A_21_P0001662 | lnc-AJAP1-3 | 0.27805 |
| A_33_P3374343 | FZD10-AS1 | 0.27808 |
| A_21_P0004021 | lnc-COX7C-1 | 0.27913 |
| A_33_P3314511 | SNRPD3 | 0.27927 |
| A_21_P0009538 | lnc-DSC3-2 | 0.28017 |
| A_23_P415541 | GPR26 | 0.28126 |
| A_33_P3365082 | OR2G6 | 0.28153 |
| A_33_P3287907 | ZBED3-AS1 | 0.28172 |
| A_21_P0002605 | LOC102724744 | 0.28220 |
| A_21_P0014449 | LOC100506532 | 0.28260 |
| A_24_P516246 | FLJ12825 | 0.28485 |
| A_33_P3377529 | HOXA4 | 0.28513 |
| A_21_P0001899 | GACAT1 | 0.28651 |
| A_33_P3380702 | OR5L1 | 0.28749 |
| A_21_P0001437 | lnc-KIAA1383-2 | 0.28892 |
| A_21_P0006077 | LOC101927305 | 0.28894 |
| A_33_P3325708 | LOC101927801 | 0.29056 |
| A_21_P0010534 | lnc-CCDC146-1 | 0.29097 |
| A_19_P00322119 | LOC102800447 | 0.29164 |
| A_21_P0010018 | LINC01384 | 0.29272 |
| A_33_P3572454 | CPEB1-AS1 | 0.29341 |
| A_33_P3424062 | KCNF1 | 0.29343 |
| A_21_P0003198 | lnc-CRYGS-1 | 0.29364 |
| A_21_P0005712 | lnc-BHLHE22-3 | 0.29367 |
| A_21_P0006836 | lnc-MCM10-1 | 0.29434 |
| A_23_P748 | IRF6 | 0.29491 |
| A_21_P0000843 | LOC100507250 | 0.29722 |
| A_33_P3262742 | DAPL1 | 0.29776 |
| A_21_P0010224 | lnc-JAM2-8 | 0.29790 |
| A_33_P3255135 | LOC101929485 | 0.29865 |
| A_23_P91636 | POM121L9P | 0.30073 |
| A_21_P0006245 | LOC102724193 | 0.30105 |
| A_21_P0006080 | C9orf135-AS1 | 0.30145 |
| A_21_P0005401 | lnc-ASB4-2 | 0.30388 |
| A_23_P75179 | CFAP46 | 0.30467 |
| A_21_P0004916 | lnc-PRDM1-2 | 0.30543 |
| A_21_P0004369 | lnc-CDH9-2 | 0.30624 |
| A_21_P0005809 | lnc-INTS9-1 | 0.30698 |
| A_21_P0008229 | lnc-ZMYM2-1 | 0.30720 |
| A_21_P0004893 | lnc-PHF3-4 | 0.30785 |
| A_33_P3364607 | LOC100130051 | 0.30813 |
| A_33_P3386150 | NRADDP | 0.30819 |
| A_23_P393777 | PTGDR | 0.30857 |
| A_21_P0008182 | LINC01069 | 0.30905 |
| A_32_P203404 | FAM69C | 0.30962 |
| A_24_P930111 | SLC4A10 | 0.30987 |
| A_33_P3357738 | lnc-C11orf88-1 | 0.31013 |
| A_23_P39067 | SPIB | 0.31129 |
| A_23_P502081 | MAGEC3 | 0.31165 |
| A_21_P0014427 | lnc-CHADL-1 | 0.31173 |
| A_33_P3385706 | OR52I1 | 0.31212 |
| A_23_P253317 | GPR171 | 0.31213 |
| A_21_P0014488 | LOC101928658 | 0.31244 |
| A_21_P0007863 | LOC101930457 | 0.31330 |
| A_21_P0012402 | LINC01210 | 0.31362 |
| A_21_P0004475 | lnc-PROP1-3 | 0.31495 |
| A_33_P3261463 | LINC00982 | 0.31618 |
| A_33_P3266823 | VWA5B2 | 0.31838 |
| A_24_P59735 | SIAH3 | 0.31897 |
| A_21_P0006486 | lnc-CXorf22-1 | 0.31916 |
| A_33_P3254136 | PKHD1L1 | 0.31942 |
| A_23_P34424 | KCNQ4 | 0.31951 |
| A_24_P409230 | OR10A2 | 0.32107 |
| A_21_P0004371 | lnc-DROSHA-7 | 0.32156 |
| A_21_P0007658 | LOC101927491 | 0.32193 |
| A_21_P0003076 | lnc-DNAJB11-1 | 0.32205 |
| A_21_P0013458 | LINC01287 | 0.32264 |
| A_33_P3294821 | OTOP1 | 0.32357 |
| A_21_P0010558 | PDE4DIP | 0.32377 |
| A_23_P208182 | SIGLEC10 | 0.32407 |
| A_21_P0010996 | XLOC_l2_002469 | 0.32467 |
| A_21_P0001649 | lnc-PLD5-1 | 0.32490 |
| A_19_P00321178 | LOC101927136 | 0.32538 |
| A_24_P188447 | ELAVL4 | 0.32606 |
| A_21_P0008742 | LOC101928499 | 0.32611 |
| A_33_P3340808 | FLJ46026 | 0.32612 |
| A_33_P3249696 | DLGAP1-AS5 | 0.32677 |
| A_21_P0010787 | LOC102723761 | 0.32677 |
| A_21_P0013435 | LINC00174 | 0.32696 |
| A_23_P7503 | TIMD4 | 0.32790 |
| A_33_P3267185 | ATP2B1 | 0.32837 |
| A_21_P0013409 | LOC100134040 | 0.32898 |
| A_19_P00807752 | MCF2L | 0.32967 |
| A_21_P0003861 | LOC100506122 | 0.32976 |
| A_21_P0012578 | XLOC_l2_010602 | 0.32993 |
| A_21_P0005009 | lnc-MBOAT1-3 | 0.32994 |
| A_19_P00318726 | LINC00880 | 0.33019 |
| A_23_P500741 | CBFA2T3 | 0.33345 |
| A_23_P258525 | MC3R | 0.33351 |
| A_24_P65121 | TTC23 | 0.33429 |
| A_33_P3394366 | CLEC19A | 0.33457 |
| A_21_P0004205 | lnc-C5orf38-1 | 0.33512 |
| A_21_P0002182 | LINC00954 | 0.33543 |
| A_21_P0003183 | lnc-HLTF-2 | 0.33582 |
| A_23_P30976 | GRM1 | 0.33706 |
| A_23_P148255 | MAGEA2B | 0.33725 |
| A_23_P301855 | LSAMP | 0.33776 |
| A_24_P788373 | LOC100132661 | 0.34018 |
| A_33_P3389917 | MTR | 0.34123 |
| A_33_P3389306 | lnc-AGPS-2 | 0.34128 |
| A_33_P3287690 | CNOT2 | 0.34144 |
| A_21_P0012005 | LOC101927661 | 0.34159 |
| A_21_P0008774 | lnc-DET1-2 | 0.34249 |
| A_23_P1473 | PRF1 | 0.34262 |
| A_21_P0008441 | lnc-CNIH-1 | 0.34278 |
| A_21_P0006062 | lnc-PPP1R26-1 | 0.34373 |
| A_23_P99442 | FLT3 | 0.34378 |
| A_21_P0007181 | lnc-INSC-3 | 0.34520 |
| A_21_P0001966 | lnc-ATIC-6 | 0.34527 |
| A_21_P0014499 | SACS-AS1 | 0.34531 |
| A_33_P3317589 | GFRA4 | 0.34592 |
| A_23_P31444 | ERVW-1 | 0.34733 |
| A_21_P0005734 | lnc-RIPK2-1 | 0.34739 |
| A_23_P62446 | HSFY2 | 0.34777 |
| A_33_P3284132 | C10orf90 | 0.34941 |
| A_33_P3677814 | NPSA | 0.35065 |
| A_21_P0010436 | lnc-LARGE-2 | 0.35256 |
| A_33_P3240693 | THSD4 | 0.35290 |
| A_23_P131588 | BMP10 | 0.35298 |
| A_21_P0011647 | XLOC_l2_006256 | 0.35315 |
| A_21_P0014913 | LINC00458 | 0.35342 |
| A_33_P3291939 | CNGA3 | 0.35418 |
| A_23_P127824 | TNNT3 | 0.35441 |
| A_21_P0009620 | C18orf61 | 0.35441 |
| A_21_P0007752 | lnc-APPL2-2 | 0.35459 |
| A_21_P0011597 | XLOC_l2_006152 | 0.35510 |
| A_32_P70203 | FAM41C | 0.35528 |
| A_21_P0000648 | LINC00851 | 0.35592 |
| A_21_P0012515 | LINC01206 | 0.35597 |
| A_21_P0005155 | lnc-DTNBP1-2 | 0.35617 |
| A_21_P0000768 | LINC01095 | 0.35646 |
| A_21_P0012466 | XLOC_l2_010405 | 0.35701 |
| A_21_P0009869 | lnc-SLCO4A1-1 | 0.35738 |
| A_23_P38603 | KRTAP4-4 | 0.35750 |
| A_23_P257129 | PAEP | 0.35806 |
| A_23_P56787 | CNTNAP5 | 0.35806 |
| A_23_P147665 | OLFML1 | 0.35878 |
| A_33_P3210228 | ZCCHC6 | 0.35889 |
| A_21_P0006344 | lnc-DMRTA1-7 | 0.36160 |
| A_21_P0008985 | lnc-IRF8-2 | 0.36216 |
| A_33_P3396404 | CLLU1OS | 0.36256 |
| A_21_P0002300 | lnc-FMNL2-1 | 0.36305 |
| A_21_P0004496 | lnc-CDH6-2 | 0.36307 |
| A_21_P0010400 | lnc-ADRBK2-1 | 0.36355 |
| A_33_P3286352 | TMEM254-AS1 | 0.36500 |
| A_21_P0004699 | LOC102724053 | 0.36500 |
| A_21_P0000025 | NOS3 | 0.36508 |
| A_33_P3389693 | lnc-GABPA-4 | 0.36576 |
| A_23_P138262 | PADI4 | 0.36646 |
| A_21_P0001408 | lnc-CD55-1 | 0.36956 |
| A_23_P2322 | TSPAN19 | 0.37036 |
| A_21_P0001133 | lnc-FAM151A-3 | 0.37165 |
| A_21_P0011167 | XLOC_l2_003877 | 0.37238 |
| A_33_P3418005 | CCDC160 | 0.37474 |
| A_21_P0008139 | LOC102723392 | 0.37541 |
| A_21_P0005839 | lnc-CA8-1 | 0.37593 |
| A_21_P0011155 | XLOC_l2_003820 | 0.37597 |
| A_33_P3393200 | SRRM4 | 0.37644 |
| A_21_P0002268 | lnc-C2orf40-5 | 0.37736 |
| A_23_P378427 | STARD6 | 0.37748 |
| A_24_P126484 | PBLD | 0.37755 |
| A_21_P0006590 | LINC00278 | 0.37792 |
| A_21_P0009388 | lnc-TMEM98-1 | 0.37847 |
| A_21_P0002572 | LOC101929643 | 0.37910 |
| A_23_P382240 | TMEM26 | 0.37955 |
| A_33_P3222451 | AKT1S1 | 0.38103 |
| A_23_P424900 | PIFO | 0.38125 |
| A_21_P0013295 | XLOC_l2_013783 | 0.38188 |
| A_33_P3367924 | PABPN1L | 0.38206 |
| A_33_P3265309 | CEACAM4 | 0.38245 |
| A_33_P3391915 | BTBD17 | 0.38302 |
| A_33_P3386099 | ELK1 | 0.38328 |
| A_21_P0008593 | lnc-C15orf2-5 | 0.38331 |
| A_19_P00318443 | lnc-MAGI2-1 | 0.38375 |
| A_21_P0008814 | lnc-C15orf2-2 | 0.38432 |
| A_24_P334248 | PLCH1 | 0.38564 |
| A_21_P0001283 | lnc-AL591845.1-1 | 0.38619 |
| A_21_P0014490 | LOC728095 | 0.38621 |
| A_23_P119042 | NKG7 | 0.38664 |
| A_24_P417664 | CC2D2B | 0.38805 |
| A_23_P865 | FRRS1 | 0.38860 |
| A_23_P151232 | TMEM132C | 0.38869 |
| A_21_P0006130 | lnc-DMRTA1-7 | 0.38957 |
| A_23_P302634 | OTOGL | 0.38998 |
| A_33_P3308045 | EIF4E2 | 0.39009 |
| A_21_P0003058 | LINC01330 | 0.39014 |
| A_33_P3829391 | LOC641510 | 0.39027 |
| A_21_P0002264 | lnc-MRPS9-1 | 0.39084 |
| A_21_P0000858 | LOC100506207 | 0.39108 |
| A_24_P254506 | PAGE4 | 0.39146 |
| A_24_P6626 | TMEM132B | 0.39179 |
| A_21_P0007668 | lnc-FZD10-3 | 0.39193 |
| A_24_P286569 | LANCL2 | 0.39252 |
| A_21_P0009092 | DNASE1 | 0.39283 |
| A_23_P212968 | UGT2B11 | 0.39375 |
| A_33_P3292254 | FAM205BP | 0.39378 |
| A_33_P3374318 | lnc-MFSD9-4 | 0.39419 |
| A_32_P211418 | PIH1D3 | 0.39533 |
| A_21_P0009988 | lnc-LSM14B-1 | 0.39655 |
| A_21_P0008145 | lnc-LHFP-2 | 0.39659 |
| A_23_P212241 | CHL1 | 0.39735 |
| A_33_P3224735 | ZC3H12B | 0.39782 |
| A_21_P0003029 | lnc-KBTBD12-1 | 0.39841 |
| A_21_P0005139 | lnc-UTRN-1 | 0.39857 |
| A_23_P34183 | XKRY2 | 0.40047 |
| A_21_P0008088 | lnc-NDFIP2-3 | 0.40059 |
| A_21_P0012401 | XLOC_l2_009889 | 0.40097 |
| A_23_P350808 | UNC80 | 0.40121 |
| A_21_P0010896 | LINC00864 | 0.40124 |
| A_23_P951 | CFHR2 | 0.40135 |
| A_33_P3416376 | C19orf81 | 0.40173 |
| A_24_P321125 | LRRC18 | 0.40227 |
| A_24_P933908 | GPNMB | 0.40447 |
| A_21_P0004884 | lnc-RUNX2-1 | 0.40470 |
| A_21_P0009563 | lnc-LMAN1-2 | 0.40500 |
| A_33_P3315982 | SCGB2B3P | 0.40524 |
| A_21_P0001664 | LOC100505887 | 0.40582 |
| A_24_P309095 | RELN | 0.40618 |
| A_33_P3389336 | LOC101929918 | 0.40725 |
| A_21_P0010550 | XLOC_l2_000384 | 0.40792 |
| A_21_P0008218 | lnc-TUBGCP3-7 | 0.40846 |
| A_21_P0011519 | XLOC_l2_005691 | 0.40870 |
| A_33_P3857239 | KRT42P | 0.40961 |
| A_23_P5778 | RAB17 | 0.40977 |
| A_23_P30603 | DDO | 0.41118 |
| A_33_P3317618 | SYN2 | 0.41156 |
| A_33_P3233749 | LOC100128751 | 0.41208 |
| A_21_P0010397 | lnc-RGL4-4 | 0.41208 |
| A_21_P0000928 | LOC115110 | 0.41322 |
| A_21_P0010203 | LINC01423 | 0.41328 |
| A_21_P0010824 | XLOC_l2_001573 | 0.41364 |
| A_21_P0001466 | lnc-MTOR-1 | 0.41470 |
| A_23_P386912 | UGT2B4 | 0.41504 |
| A_24_P314515 | HNF1A-AS1 | 0.41550 |
| A_23_P500400 | ABCA6 | 0.41557 |
| A_21_P0014459 | lnc-C2orf49-2 | 0.41634 |
| A_19_P00319588 | LOC101929562 | 0.41652 |
| A_21_P0007052 | LINC00202-2 | 0.41664 |
| A_33_P3387493 | FTH1P18 | 0.41743 |
| A_33_P3220738 | ASTN1 | 0.41756 |
| A_21_P0014786 | LOC100507663 | 0.41766 |
| A_33_P3309175 | PLEKHG2 | 0.41780 |
| A_21_P0003761 | lnc-RP11-455G16.1.1-1 | 0.41783 |
| A_23_P405878 | C12orf54 | 0.41789 |
| A_24_P310256 | LGI4 | 0.41871 |
| A_23_P310701 | MAP10 | 0.41900 |
| A_33_P3245858 | LOC100131581 | 0.41927 |
| A_33_P3225397 | LOC102724030 | 0.41932 |
| A_21_P0010268 | lnc-AF165138.7.1-2 | 0.41975 |
| A_24_P169402 | DNAH12 | 0.42018 |
| A_33_P3343316 | SH3BGRL2 | 0.42039 |
| A_23_P156025 | IRX2 | 0.42060 |
| A_33_P3434927 | MAGI2-IT1 | 0.42116 |
| A_19_P00321761 | IWS1 | 0.42121 |
| A_23_P90944 | SCN7A | 0.42137 |
| A_23_P383590 | LINC01140 | 0.42160 |
| A_23_P21943 | SAGE1 | 0.42164 |
| A_23_P34852 | CTRC | 0.42223 |
| A_21_P0007940 | lnc-PCDH17-1 | 0.42295 |
| A_21_P0004270 | lnc-ATG10-1 | 0.42299 |
| A_24_P910688 | C19orf67 | 0.42307 |
| A_23_P13442 | MICAL2 | 0.42380 |
| A_19_P00322409 | STXBP5-AS1 | 0.42392 |
| A_21_P0012423 | XLOC_l2_010056 | 0.42428 |
| A_33_P3846114 | LINC01281 | 0.42519 |
| A_33_P3388646 | ABLIM1 | 0.42574 |
| A_33_P3409090 | CNTN1 | 0.42592 |
| A_23_P94186 | LYPD2 | 0.42705 |
| A_21_P0007372 | lnc-FCHSD2-3 | 0.42723 |
| A_23_P431923 | SSX8 | 0.42810 |
| A_23_P173 | CASQ1 | 0.42887 |
| A_21_P0004854 | lnc-NRSN1-2 | 0.42890 |
| A_23_P7835 | EPHA7 | 0.42908 |
| A_21_P0008094 | LINC00377 | 0.42909 |
| A_21_P0008417 | lnc-PRKD1-2 | 0.42945 |
| A_21_P0001191 | LOC101928565 | 0.42989 |
| A_33_P3255290 | JAKMIP2 | 0.43012 |
| A_21_P0012585 | XLOC_l2_010679 | 0.43035 |
| A_21_P0005531 | lnc-AKR1B1-1 | 0.43037 |
| A_23_P388220 | RALYL | 0.43086 |
| A_21_P0012532 | GRM7-AS3 | 0.43112 |
| A_33_P3295278 | C1orf185 | 0.43197 |
| A_33_P3291445 | PIGK | 0.43306 |
| A_21_P0006283 | LOC101927450 | 0.43311 |
| A_21_P0006325 | lnc-C9orf50-5 | 0.43313 |
| A_24_P65722 | LILRB4 | 0.43378 |
| A_21_P0007021 | lnc-VAX1-1 | 0.43400 |
| A_21_P0002278 | lnc-GLI2-4 | 0.43446 |
| A_33_P3211666 | IL18R1 | 0.43463 |
| A_21_P0013996 | TXLNGY | 0.43533 |
| A_21_P0002261 | lnc-IL1R2-1 | 0.43545 |
| A_23_P41241 | TGM4 | 0.43550 |
| A_33_P3271684 | TMEM236 | 0.43569 |
| A_21_P0013467 | FAM66C | 0.43596 |
| A_33_P3301884 | LINC01257 | 0.43603 |
| A_21_P0012862 | XLOC_l2_011854 | 0.43634 |
| A_23_P141624 | KRTAP1-1 | 0.43667 |
| A_33_P3402968 | GAB4 | 0.43700 |
| A_21_P0004506 | lnc-SREK1-1 | 0.43745 |
| A_24_P256512 | STH | 0.43746 |
| A_21_P0006967 | lnc-C10orf31-7 | 0.43770 |
| A_21_P0001396 | lnc-NPL-1 | 0.43800 |
| A_23_P79217 | LCT | 0.43816 |
| A_33_P3311974 | PROX2 | 0.43909 |
| A_21_P0005136 | lnc-TNFAIP3-1 | 0.43923 |
| A_21_P0013522 | LOC101929709 | 0.43946 |
| A_33_P3231319 | XLOC_l2_013192 | 0.43989 |
| A_33_P3374378 | GTDC1 | 0.44020 |
| A_21_P0005710 | lnc-NKAIN3-6 | 0.44036 |
| A_21_P0000526 | ZSCAN18 | 0.44045 |
| A_21_P0003972 | lnc-FBXL7-3 | 0.44049 |
| A_21_P0005410 | lnc-ZKSCAN1-1 | 0.44140 |
| A_23_P256084 | ARSE | 0.44208 |
| A_21_P0003848 | LOC101929654 | 0.44218 |
| A_33_P3361896 | LOC100127994 | 0.44223 |
| A_33_P3405680 | ARHGEF3-AS1 | 0.44277 |
| A_21_P0007202 | HSD17B12 | 0.44315 |
| A_21_P0002763 | LINC00883 | 0.44316 |
| A_33_P3269570 | CD160 | 0.44369 |
| A_21_P0001829 | lnc-DDX1-1 | 0.44393 |
| A_21_P0001489 | lnc-TRIM62-1 | 0.44483 |
| A_23_P427023 | GIMAP1 | 0.44517 |
| A_21_P0009947 | lnc-SSTR4-4 | 0.44538 |
| A_33_P3406047 | NLRP4 | 0.44641 |
| A_21_P0002441 | lnc-C2orf63-1 | 0.44674 |
| A_33_P3395581 | TRIM53AP | 0.44753 |
| A_21_P0008761 | lnc-ADAMTS7-1 | 0.44774 |
| A_21_P0006444 | lnc-MAP7D2-1 | 0.44791 |
| A_21_P0008258 | LINC00376 | 0.44793 |
| A_21_P0009364 | lnc-CD300C-2 | 0.44799 |
| A_21_P0003810 | lnc-CLDN24-3 | 0.44815 |
| A_23_P117896 | TGM7 | 0.44919 |
| A_21_P0008048 | lnc-NBEA-3 | 0.45014 |
| A_19_P00318183 | LINC01197 | 0.45086 |
| A_21_P0008882 | LOC100507534 | 0.45129 |
| A_21_P0014362 | LOC102724715 | 0.45137 |
| A_21_P0009954 | lnc-BPIFA2-1 | 0.45141 |
| A_21_P0004550 | lnc-SLC12A7-1 | 0.45152 |
| A_23_P323196 | MDS2 | 0.45197 |
| A_33_P3236833 | GPR12 | 0.45245 |
| A_21_P0009992 | lnc-COL20A1-3 | 0.45353 |
| A_23_P84154 | ARHGAP15 | 0.45411 |
| A_33_P3283592 | C5orf60 | 0.45446 |
| A_21_P0008506 | lnc-FOXG1-5 | 0.45450 |
| A_33_P3211558 | ERBB4 | 0.45495 |
| A_23_P252023 | SSX2 | 0.45497 |
| A_21_P0000767 | SLIT1-AS1 | 0.45546 |
| A_32_P206479 | ZNF831 | 0.45577 |
| A_21_P0008092 | lnc-NDFIP2-8 | 0.45587 |
| A_24_P213120 | TRIM64 | 0.45591 |
| A_33_P3362331 | DEFB130 | 0.45593 |
| A_19_P00804359 | LINC01488 | 0.45623 |
| A_33_P3213665 | LOC728084 | 0.45649 |
| A_33_P3420020 | PPIP5K1 | 0.45668 |
| A_33_P3419557 | OR5K3 | 0.45689 |
| A_33_P3243023 | ADRA1D | 0.45708 |
| A_32_P184394 | TFEC | 0.45717 |
| A_21_P0005564 | lnc-ZNF736-2 | 0.45768 |
| A_21_P0003180 | lnc-MSL2-1 | 0.45795 |
| A_21_P0001591 | lnc-MPZ-1 | 0.45810 |
| A_23_P78201 | KRT35 | 0.45819 |
| A_21_P0000845 | MIPEPP3 | 0.45905 |
| A_21_P0011442 | XLOC_l2_005245 | 0.45952 |
| A_23_P340019 | NLRC3 | 0.45987 |
| A_23_P204087 | OAS2 | 0.46049 |
| A_23_P81131 | CORIN | 0.46095 |
| A_33_P3299309 | RPL28 | 0.46118 |
| A_23_P302595 | LINC00518 | 0.46137 |
| A_33_P3229417 | NRG3 | 0.46146 |
| A_21_P0000853 | ELOVL2-AS1 | 0.46249 |
| A_21_P0010434 | lnc-MN1-2 | 0.46339 |
| A_33_P3785051 | EFCAB10 | 0.46344 |
| A_32_P9986 | lnc-PHF10-1 | 0.46353 |
| A_33_P3222424 | CSF3 | 0.46416 |
| A_21_P0009405 | LOC102723471 | 0.46471 |
| A_33_P3364038 | IRGM | 0.46480 |
| A_24_P935794 | FAM150A | 0.46527 |
| A_24_P315346 | POM121L8P | 0.46528 |
| A_33_P3249364 | TMTC1 | 0.46551 |
| A_24_P120109 | DHX57 | 0.46554 |
| A_19_P00317087 | LOC101929450 | 0.46569 |
| A_23_P145529 | PKIB | 0.46570 |
| A_33_P3356877 | OR13C3 | 0.46583 |
| A_19_P00315493 | LOC145474 | 0.46621 |
| A_23_P42897 | MGAM | 0.46647 |
| A_21_P0001513 | lnc-JUN-6 | 0.46652 |
| A_21_P0011982 | LINC01473 | 0.46666 |
| A_24_P327084 | PRSS33 | 0.46680 |
| A_33_P3411628 | CDKN2A | 0.46710 |
| A_33_P3265526 | NAA16 | 0.46717 |
| A_23_P67932 | CXCR1 | 0.46735 |
| A_33_P3257728 | C1RL | 0.46758 |
| A_33_P3362826 | ANKRD31 | 0.46783 |
| A_33_P3280759 | LOC400867 | 0.46798 |
| A_32_P142779 | SPPL2C | 0.46853 |
| A_33_P3281552 | RPGR | 0.46853 |
| A_21_P0008661 | lnc-FAH-1 | 0.46927 |
| A_21_P0009276 | lnc-SEPT9-1 | 0.47002 |
| A_33_P3411338 | ENPEP | 0.47035 |
| A_33_P3310164 | DGKB | 0.47055 |
| A_21_P0009808 | lnc-AC012313.1-1 | 0.47060 |
| A_24_P297551 | FAM19A2 | 0.47077 |
| A_33_P3288824 | H2AFB3 | 0.47131 |
| A_21_P0005893 | lnc-EXT1-1 | 0.47134 |
| A_21_P0009933 | lnc-C20orf187-2 | 0.47135 |
| A_21_P0000695 | LOC731424 | 0.47141 |
| A_21_P0009899 | lnc-PXMP4-1 | 0.47170 |
| A_33_P3304304 | STAG2 | 0.47428 |
| A_21_P0012438 | TRANK1 | 0.47450 |
| A_21_P0000823 | PAXBP1-AS1 | 0.47452 |
| A_21_P0007772 | lnc-TAOK3-3 | 0.47468 |
| A_24_P57047 | DLL3 | 0.47468 |
| A_33_P3389852 | BCAS3 | 0.47501 |
| A_32_P234145 | SHC4 | 0.47547 |
| A_21_P0001976 | lnc-ITM2C-1 | 0.47578 |
| A_21_P0007292 | lnc-RP11-50B3.2.1-2 | 0.47583 |
| A_33_P3314401 | CLDN16 | 0.47606 |
| A_23_P69329 | HYAL1 | 0.47644 |
| A_21_P0004996 | lnc-RPP40-1 | 0.47657 |
| A_21_P0002541 | lnc-TNP1-2 | 0.47676 |
| A_23_P156390 | JAKMIP2 | 0.47689 |
| A_33_P3263287 | LOC100129111 | 0.47745 |
| A_33_P3240437 | LOC642335 | 0.47748 |
| A_33_P3287862 | LOC101060524 | 0.47769 |
| A_33_P3318530 | LOC441204 | 0.47814 |
| A_21_P0007375 | lnc-LRRC32-5 | 0.47837 |
| A_19_P00316164 | LOC101928858 | 0.47863 |
| A_23_P36187 | SYT8 | 0.47868 |
| A_33_P3304678 | LINC00654 | 0.47931 |
| A_33_P3388453 | NANOS2 | 0.47965 |
| A_23_P214300 | GSTA2 | 0.47981 |
| A_21_P0013958 | PDCD6 | 0.47987 |
| A_23_P308632 | C19orf26 | 0.47997 |
| A_33_P3278571 | MAGIX | 0.47999 |
| A_23_P60837 | PDE3A | 0.48032 |
| A_33_P3269208 | STX19 | 0.48062 |
| A_23_P212608 | CLSTN2 | 0.48063 |
| A_33_P3274599 | FAM138E | 0.48078 |
| A_21_P0004986 | lnc-GMDS-4 | 0.48086 |
| A_23_P23869 | LHX4 | 0.48112 |
| A_24_P375205 | MKL2 | 0.48167 |
| A_21_P0002869 | LINC00635 | 0.48233 |
| A_21_P0010968 | WT1-AS | 0.48253 |
| A_24_P401174 | KIAA0556 | 0.48262 |
| A_33_P3236628 | BCKDHA | 0.48349 |
| A_33_P3419113 | PRY2 | 0.48356 |
| A_33_P3405399 | ODF2L | 0.48369 |
| A_33_P3876192 | IGLV1-44 | 0.48376 |
| A_23_P34345 | VCAM1 | 0.48396 |
| A_21_P0003613 | lnc-POLR2B-2 | 0.48400 |
| A_33_P3392867 | PPEF2 | 0.48429 |
| A_32_P54037 | CCDC38 | 0.48435 |
| A_33_P3420496 | SNORA53 | 0.48462 |
| A_33_P3389802 | LOC101928235 | 0.48463 |
| A_21_P0013159 | LINC01277 | 0.48518 |
| A_23_P120629 | ASIP | 0.48526 |
| A_33_P3234025 | KAZN | 0.48543 |
| A_23_P346673 | GPRC5C | 0.48550 |
| A_33_P3293411 | lnc-MLL3-1 | 0.48557 |
| A_33_P3303319 | LPPR5 | 0.48574 |
| A_21_P0001277 | lnc-PTPRU-2 | 0.48583 |
| A_23_P13294 | OR1S2 | 0.48586 |
| A_21_P0014872 | LOC100505923 | 0.48590 |
| A_33_P3272493 | CD209 | 0.48595 |
| A_24_P935682 | LOC100131541 | 0.48604 |
| A_21_P0011205 | LINC01309 | 0.48617 |
| A_21_P0006476 | lnc-AKAP17A-1 | 0.48625 |
| A_21_P0009773 | lnc-BBC3-1 | 0.48632 |
| A_33_P3228862 | LRRC69 | 0.48642 |
| A_19_P00805942 | SLC7A14 | 0.48666 |
| A_21_P0006622 | GATA3-AS1 | 0.48741 |
| A_21_P0009611 | LOC101927481 | 0.48762 |
| A_21_P0012738 | LOC100507487 | 0.48792 |
| A_23_P6422 | RFPL3 | 0.48860 |
| A_21_P0011018 | LOC101928620 | 0.48904 |
| A_21_P0001552 | lnc-ALG14-3 | 0.48934 |
| A_21_P0006308 | lnc-KLF4-2 | 0.48947 |
| A_33_P3355208 | CROT | 0.48949 |
| A_33_P3214948 | SPOCK2 | 0.48963 |
| A_32_P176594 | KIAA1614 | 0.49013 |
| A_19_P00322152 | LINC00607 | 0.49032 |
| A_23_P256470 | NPY | 0.49032 |
| A_21_P0006225 | lnc-GAPVD1-2 | 0.49095 |
| A_21_P0011007 | TRIM49B | 0.49146 |
| A_33_P3360249 | HCRTR1 | 0.49148 |
| A_33_P3406651 | ZSCAN5B | 0.49155 |
| A_33_P3424217 | HLA-DQB1 | 0.49159 |
| A_23_P17456 | SIRPB1 | 0.49180 |
| A_21_P0010179 | lnc-TMPRSS15-1 | 0.49185 |
| A_19_P00803775 | lnc-AC136604.1-1 | 0.49189 |
| A_32_P466877 | LSMEM2 | 0.49191 |
| A_33_P3303729 | XLOC_l2_013415 | 0.49268 |
| A_33_P3334015 | NATD1 | 0.49274 |
| A_21_P0005454 | lnc-WDR60-6 | 0.49279 |
| A_24_P204819 | SPRY3 | 0.49293 |
| A_21_P0009746 | lnc-ZNF724P-2 | 0.49312 |
| A_33_P3415113 | CLCN6 | 0.49390 |
| A_21_P0012625 | LOC101927849 | 0.49410 |
| A_24_P357535 | OR7A10 | 0.49431 |
| A_33_P3250944 | C1orf189 | 0.49441 |
| A_23_P8820 | FABP4 | 0.49444 |
| A_23_P31945 | IL33 | 0.49445 |
| A_23_P165042 | LOC100129935 | 0.49466 |
| A_33_P3354429 | TRIM71 | 0.49487 |
| A_21_P0006171 | LOC100506834 | 0.49488 |
| A_21_P0008988 | lnc-FOXL1-3 | 0.49516 |
| A_33_P3239914 | LOC100130741 | 0.49527 |
| A_21_P0014035 | MUC19 | 0.49607 |
| A_23_P217277 | SLITRK2 | 0.49704 |
| A_23_P357966 | PCMTD1 | 0.49728 |
| A_21_P0001270 | lnc-CDC42-1 | 0.49761 |
| A_33_P3307008 | CCDC168 | 0.49761 |
| A_21_P0011699 | XLOC_l2_006745 | 0.49780 |
| A_21_P0003101 | VGLL4 | 0.49798 |
| A_24_P187351 | CNOT4 | 0.49842 |
| A_21_P0012025 | LINC01412 | 0.49844 |
| A_19_P00316349 | LOC101928502 | 0.49858 |
| A_33_P3306619 | KRTAP25-1 | 0.49865 |
| A_21_P0006173 | LOC100506834 | 0.49877 |
| A_23_P170534 | FUT7 | 0.49896 |
| A_21_P0005031 | lnc-KHDRBS2-1 | 0.49932 |
| A_33_P3291748 | DCDC5 | 0.49942 |
| A_24_P170774 | LRCH1 | 0.49965 |
| A_23_P23996 | MAT1A | 0.49966 |
| A_21_P0012122 | LINC01370 | 0.49976 |
| A_21_P0010645 | XLOC_l2_001076 | 0.49985 |
| A_21_P0004308 | lnc-NDFIP1-1 | 0.49994 |

*Fold change shows the expression level for H2452 treated by anti-S100A11 antibody relative to parental H2452.
